# Phylogenetic Comparison of *F-Box* (*FBX*) Gene Superfamily within the Plant Kingdom Reveals Divergent Evolutionary Histories Indicative of Genomic Drift

**DOI:** 10.1371/journal.pone.0016219

**Published:** 2011-01-28

**Authors:** Zhihua Hua, Cheng Zou, Shin-Han Shiu, Richard D. Vierstra

**Affiliations:** 1 Department of Genetics, University of Wisconsin, Madison, Wisconsin, United States of America; 2 Department of Plant Biology, Michigan State University, East Lansing, Michigan, United States of America; The Salk Institute, United States of America

## Abstract

The emergence of multigene families has been hypothesized as a major contributor to the evolution of complex traits and speciation. To help understand how such multigene families arose and diverged during plant evolution, we examined the phylogenetic relationships of *F-Box* (*FBX*) genes, one of the largest and most polymorphic superfamilies known in the plant kingdom. FBX proteins comprise the target recognition subunit of SCF-type ubiquitin-protein ligases, where they individually recruit specific substrates for ubiquitylation. Through the extensive analysis of 10,811 *FBX* loci from 18 plant species, ranging from the alga *Chlamydomonas reinhardtii* to numerous monocots and eudicots, we discovered strikingly diverse evolutionary histories. The number of *FBX* loci varies widely and appears independent of the growth habit and life cycle of land plants, with a little as 198 predicted for *Carica papaya* to as many as 1350 predicted for *Arabidopsis lyrata*. This number differs substantially even among closely related species, with evidence for extensive gains/losses. Despite this extraordinary inter-species variation, one subset of *FBX* genes was conserved among most species examined. Together with evidence of strong purifying selection and expression, the ligases synthesized from these conserved loci likely direct essential ubiquitylation events. Another subset was much more lineage specific, showed more relaxed purifying selection, and was enriched in loci with little or no evidence of expression, suggesting that they either control more limited, species-specific processes or arose from genomic drift and thus may provide reservoirs for evolutionary innovation. Numerous *FBX* loci were also predicted to be pseudogenes with their numbers tightly correlated with the total number of *FBX* genes in each species. Taken together, it appears that the *FBX* superfamily has independently undergone substantial birth/death in many plant lineages, with its size and rapid evolution potentially reflecting a central role for ubiquitylation in driving plant fitness.

## Introduction

The emergence and expansion of gene families and subsequent functional divergence among family members have long been hypothesized to be the principal driving forces in the adaptation of organisms to different environments [1]. Following their initial appearance, gene paralogs can subfunctionalize to extend and diversify their biological roles or can neo-functionalize to generate new traits. Family members can also acquire detrimental mutations that marginalize their activities or become inactivated, leading to the creation of pseudogenes. Some families, such as those encoding transcription factors, tend to have high rates of duplication (birth) but low rates of pseudogenization and loss (death) [2], [3]. At the other extreme, families with high birth and death rates have been found, including those encoding in animals the G protein-coupled chemosensory receptors, which detect food, ordants and pheromones [4], the immunoglobulins which form the primary immune defense system of vertebrates [5], and the nucleotide-binding site–leucine-rich repeat (NBS-LRR) receptors that help plants defend against pathogens, which are themselves under intense selection pressure to avoid detection [6]. Plants in particular appear to have exploited multiple gene families to adapt numerous developmental and physiological processes. These families arose, expanded, and diversified via whole genome duplications (polyploidy), which are common in many plant lineages, more restricted segmental duplications, highly specific tandem duplications and transposon-mediated events, and even exon shuffling among individual genes [7], [8], [9], [10], [11]. For example, it has been estimated that 65% of the *Arabidopsis thaliana* genes have closely related paralogs, with 17% of them generated from recent tandem duplications [7].

To help understand how divergent plant gene families evolved, we studied the expansion, evolutionary selection, and functional correlations of the *F-Box* (*FBX*) superfamily, which represents one of the largest and most diverse gene families in the plant kingdom [10], [12], [13], [14], [15]. FBX proteins comprise the target recognition subunit of SCF-type ubiquitin (Ub)-protein ligases (or E3s). They are defined by a signature ∼40-amino-acid FBX domain (FBXD) at their N-termini. The FBXD forms a compact trihelical structure that promotes docking with a SKP1 protein bridge, which helps associate the FBX protein with the rest of the ligase complex containing the Cullin-1 scaffold and RBX1 proteins [16], [17]. C-terminal to the FBXD is a variable recruitment module that binds to and delivers appropriate substrates for ubiquitylation. Some well-characterized recruitment modules include leucine-rich (LRR), kelch, WD-40 and tetratricopeptide repeats (TPR), Tubby, armadillo and lectin-related, which provide interaction surfaces for specific substrates [12], [15]. Often the ubiquitylated target is subsequently degraded by the 26S proteasome but non-proteolytic consequences have also been described [18].

*A. thaliana* alone was reported to contain approximately 700 *FBX* genes [12], [15], [19], [20], with this extraordinary number providing one of the first indications as to the importance of SCF E3s in the control of plant protein abundance. By comparison, only 20, 27, and 69 FBX proteins are encoded by the yeast (*Saccharomyces cerevisiae*), *Drosophila melanogaster*, and human genomes, respectively [21]. Such a pervasive role has also been supported by genetic studies linking specific plant FBX proteins to numerous processes, including hormone perception and signaling, stress protection, chromatin remodeling, homeosis, circadian rhythms, self incompatibility, and the defense against pathogens [17], [18], [22]. A connection of plant FBX proteins and other E3 components to the adaptive evolution of plant innate immunity has also been proposed [14], [23].

The sheer number of *FBX* genes in plants combined with evidence for extensive domain shuffling and polymorphisms and an atomic scale appreciation of their corresponding protein activities (*e.g.*, [24], [25]) makes them excellent candidates to understand how plant gene families arose and diversified. Gagne *et al.* [12] provide the first detailed phylogenetic description of *FBX* genes using *A. thaliana* as the example. In addition to revealing the magnitude of the plant superfamily and the inclusion of numerous target-recruitment modules, they also detected frequent duplications of specific subsets along with potential domain shuffling between FBXD subtypes and the various recruitment modules. Subsequent phylogenetic analyses with *Oryza sativa* (rice), *Populus trichocarpa* (poplar), *Vitis vinifera* (grape), and *Carica papaya* (papaya) also identified large complements of *FBX* genes, but found substantial between species variation, with 680, 320, 159, and 139 loci estimated in each species, respectively [10], [15], [19]. High *FBX* gene numbers in herbaceous *O. sativa* and *A. thaliana* compared to the perennials *P. trichocarpa* and *V. vinifera* raised the possibility that the large *FBX* gene superfamilies in annuals reflected selection pressures to conform their ephemeral life cycles to a single growing season [19]. In addition to a set common to most of these species which likely directs conserved ubiquitylation reactions [15], [19], Xu *et al.* [15] also detected a more rapidly evolving set in *A. thaliana* which appears to have arisen by unusually frequent adjustments of exon/intron boundaries and frameshift mutations. Unfortunately, these limited phylogenetic studies: (i) failed to account for the likely presence of pseudogenes which may be common to the *FBX* superfamily [3], [26]; (ii) included very few plant species; (iii) did not infer orthologous relationships necessary to assess patterns of *FBX* gene gains and losses; and (iv) often failed to exhaustively search for all potential *FBX* loci, leaving their final gene numbers and conclusions uncertain.

Here, we studied the evolutionary history of plant *FBX* genes by identifying members of this superfamily in 18 plant species, ranging from the unicellular green alga (*Chlamydomonas reinhardtii*) and the seedless plants *Physcomitrella patens* (moss) and *Selaginella moellendorffii* (lycopod) to 15 monocot and eudicot species (both annual and perennial). Our data confirm that the number of *FBX* loci varies widely among plant species but shows little correlation with their genome size, growth habit, or annual/perennial life cycles. Moreover, the number of *FBX* loci can vary substantially even among closely related species, indicating that significant lineage/species-specific gene gains/losses occurred. A subset of highly expressed FBX proteins was detected in nearly all terrestrial species examined, suggesting that they perform functions essential to life on land. In addition, we discovered a substantial and likely non-functional collection of highly polymorphic, lineage/species-specific *FBX* genes and pseudogenes, strongly suggesting that genomic drift contributed to plant FBX protein diversification.

## Results

### Comprehensive Identification of *FBX* Genes Reveals Dramatically Varied Gene Numbers in the Plant Kingdom

**Figure 1:**
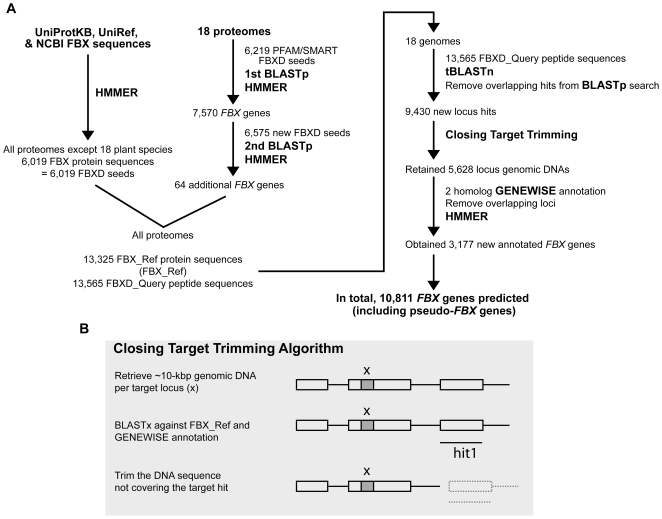
Pipeline for the comprehensive annotation of *FBX* genes in 18 plant species. (A) Procedures of *FBX* gene identification from 18 plant genomes. The initial FBXD query collection from PFAM and SMART was used to find the FBX proteins by BLASTp searches and HMMER3 predictions of each plant proteome and then incorporated with the FBX proteins collected from the other plant and non-plant proteomes to create two comprehensive sequence collections, FBX_Query and FBX_Ref. FBX_Query was used to search each genome for *FBXD* regions by tBLASTn and the non-annotated regions surrounding the *FBXD* were re-annotated by the Closing Target Trimming (CTT) algorithm and the similarity-based annotation program GENEWISE. To avoid the bias, at least two reference sequences from FBX_Ref collection were used to predict the transcript model of each non-annotated potential *FBX* locus. Only when both sequences predicted the same transcript model was the coding sequence generated with the best GENEWISE score accepted as the final annotation. (B) Schematic description of the CTT algorithm. The “x” indicates a target *FBXD* region identified from tBLASTn search. “Hit1” indicates the top hit from a BLASTx search using the ∼10-kbp genomic DNA sequence as query against FBX_Ref. The process was iterated for up to 6 times for each non-annotated potential *FBX* locus.

To better understand the organization and evolutionary history of the FBX superfamily in the plant kingdom, we initiated a comprehensive examination of the corresponding genes from all 18 complete or nearly complete plant genomic sequences available as of January 10, 2010 in Phytozome V5 (http://www.phytozome.net/). This database included a wide range of species, thus allowing us to analyze the ∼450 million year evolutionary ascent of multicellular land plants as they evolved presumably from single-cell aquatic progenitors (Table S1). To exhaustively query these genomes, we built a comprehensive *FBX* gene annotation pipeline and compiled two extensive collections of FBX protein sequences from both plant and non-plant sources (Figure 1). The first collection contained 13,565 FBXD peptide sequences (FBXD_Query, File S1), which was used to initially identify potential *FBX* genes in each genome. The second collection, containing 13,325 full-length FBX protein sequences (FBX_Ref, File S2), was then employed to develop transcript models and to predict coding sequences in previously non-annotated genes (we designated a gene non-annotated if it was not present previously in the corresponding proteome database). The FBXD_Query collection was used to exhaustively search the 18 unmasked plant genomes for *FBXD* regions by tBLASTn using an E-value cut off ≤1, Table S2) [27]. Preliminary studies with the *A. thaliana* superfamily found that this E-value was necessary to include all known *FBX* loci characterized either genetically or by interactions studies (see below and Tables S3 and S4). In addition to finding most, if not all, previously annotated *FBXD*-encoding loci [10], [12], [14], [15], [19], [20], we discovered 9,430 new regions, each of which may be part of a previously non-annotated *FBX* locus (Figure 1A, Table S2).

Initial studies showed that the coding region (plus intervening introns) of most annotated plant *FBX* genes is less then 5 kbp (95.3%), with a few between 5 kbp and 10 kbp (3.8%), and almost none over 10 kbp (1%) (Figure S1). Because our initial approach would only identify regions with high sequence similarity but not the full-length coding sequence, we retrieved a 10-kbp window surrounding each new *FBXD* region to obtain a more complete *FBX* gene model, using the sequence similarity-based predictions of GENEWISE with the FBX_Ref collection serving as the scaffolds. Because plant *FBX* genes frequently appear in tandem (*e.g*., [12]), some of these 10-kpb genomic windows likely include multiple *FBX* loci. To overcome this complication, we developed the Closing Target Trimming (CTT) algorithm to computationally trim the transcriptional unit of each *FBX* gene in the window and separate it from adjacent *FBX* loci (Figure 1B). For final annotation of each transcriptional unit (protein-coding gene or pseudogene), we used the two or three top-scored (bit-score value) protein reference sequences in the GENEWISE prediction, obtained by first querying the FBX_Ref collection using BLASTx (E-value ≤1e-5) [27]. From preliminary analysis of 47 well-characterized *A. thaliana FBX* genes (Table S3), we found that two or more references were required for unbiased annotations. Thus, only when at least two reference sequences predicted the same transcript model (protein coding versus pseudogene) did we conclude that a good model was generated.

To further confirm that our new annotation strategy with CTT would correctly predict transcript models, we re-annotated each *A. thaliana* and *O. sativa FBX* locus predicted from the original proteomes from its 10-kbp flanking genomic DNA sequence, which was retrieved as a putative non-annotated *FBX* loci described above. The result showed that 89.3% (634/710) and 93.9% (750/799) of the originally annotated *FBX* loci from *A. thaliana* and *O. sativa*, respectively, were correctly re-annotated. These new annotations closely matched the original protein-coding genes (sequence identity ∼99% for both *A. thaliana* and *O. sativa*). In addition, we discovered a large collection of *FBX* pseudogenes (sequence identity ∼95% (*A. thaliana*) and ∼94% (*O. sativa*)), which were originally annotated as protein-coding loci. In most cases, the coding sequence models of these pseudogenes were shorter than predicted previously due to the presence of frameshifts or premature stop codons (Figure S2, File S3 and S4).

After CTT and sequence similarity-based annotations combined with HMMER3 predictions (E-value <1) using the PFAM database [28] (Version 24.0, released in October 2009, 11,912 protein families), we discovered a collection of 3,177 new *FBX* loci. When combined with 7,634 loci identified from the previously annotated proteomes, a comprehensive collection of 10,811 *FBX* genes was generated that encompassed all 18 plant species (Figure 1, Tables 1 and S2, Files S5 and S6). To support the depth of the collection, we searched it for all *A. thaliana* FBX proteins previously demonstrated to interact with their SKP1 protein bridges (designated ASK1-19), or defined genetically to participate in the Ub-26S proteasome system (UPS); all 86 were found (Tables S3 and S4z).

**Table 1:** Predictions of *FBX* gene numbers in the 18 plant species.

Species^1^	Genome size(Mb)	TotalFBX(genes)	LTSP		STSP		*FBX* Pseudogenes	
			genes	% of total	genes	% of total	genes	% of total
*Al*	206.7	1350	219	16.2	761	56.4	370	27.4
*At*	119.7	897	191	21.3	507	56.5	199	22.2
*Bd*	271.9	998	177	17.7	509	51.0	312	31.3
*Cp*	331.3	198	124	62.6	35	17.7	39	19.7
*Cr*	112.3	88	9	10.2	74	84.1	5	5.7
*Cs*	203.1	207	152	73.4	46	22.2	9	4.3
*Gm*	973.3	702	332	47.3	208	29.6	162	23.1
*Me*	416.7	323	230	71.2	75	23.2	18	5.6
*Mg*	321.7	903	459	50.8	292	32.3	152	16.8
*Mt*	307.5	1148	391	34.1	517	45.0	240	20.9
*Os*	372.3	971	247	25.4	517	53.2	207	21.3
*Pp*	480	258	117	45.3	124	48.1	17	6.6
*Pt*	417.1	425	280	65.9	107	25.2	38	8.9
*Rc*	350.6	250	158	63.2	57	22.8	35	14
*Sb*	738.5	817	202	24.7	472	57.8	143	17.5
*Sm*	212.8	544	136	25.0	324	59.6	84	15.4
*Vv*	497.5	315	202	64.1	76	24.1	37	11.7
*Zm*	2061	417	175	42.0	173	41.5	69	16.5

1 Species abbreviations: *Al, Arabidopsis lyrata; At, Arabidopsis thaliana; Bd, Brachypodium distachyon; Cp, Carica papaya; Cr, Chlamydomonas reinhardtii; Cs, Cucumis sativus; Gm, Glycine max; Me, Manihot esculenta; Mt, Medicago truncatula; Mg, Mimulus guttatus; Os, Oryza sativa; Pp, Physcomitrella patens; Pt, Populus trichocarpa; Rc, Ricinus communis; Sm, Selaginella moellendorffii; Sb, Sorghum bicolor; Vv, Vitis vinifera; Zm, Zea mays.*

**Figure 2:**
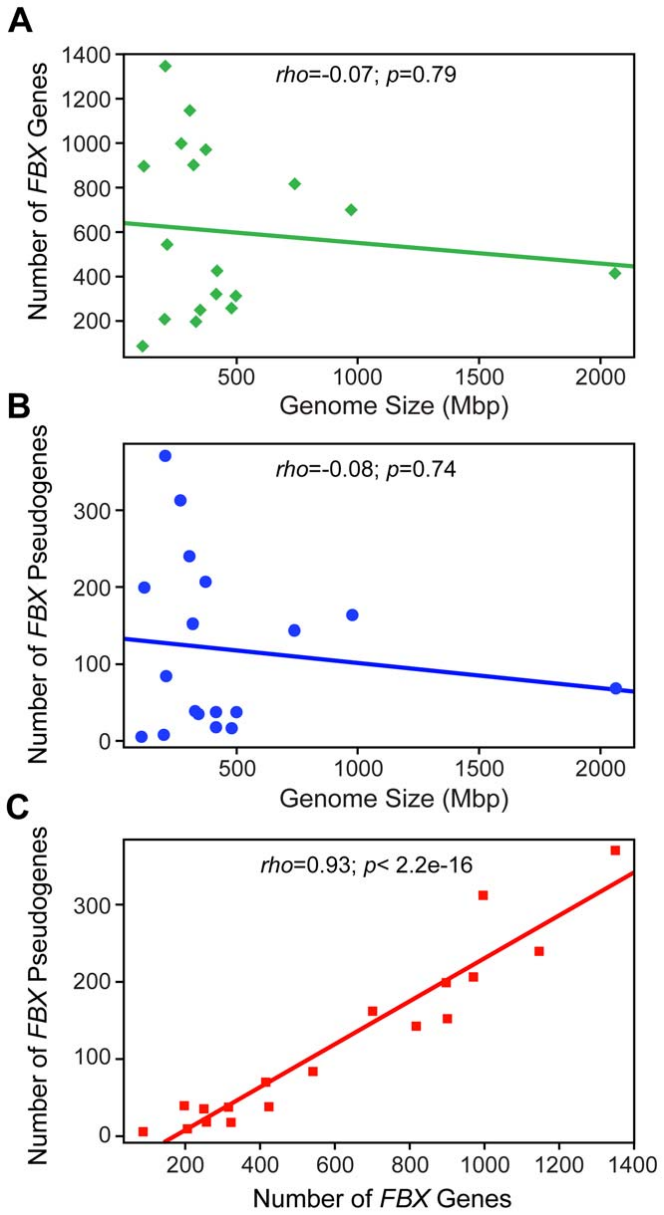
Relationship between the number of total *FBX* genes, *FBX* pseudogenes and genome size for 18 plant species. (A,B) The number of total *FBX* genes (A) and the number of *FBX* pseudogenes (B) were plotted against the genome size of each species. (C) The number of *FBX* pseudogenes was plotted against the number of total *FBX* genes in each species. The Spearman rank correlation coefficients (*rho*), the corresponding *p* values, and the linear model-fitted trend lines are shown.

Similar to earlier more-limited studies [10], [15], [19], we found widely different numbers of total *FBX* genes among the 18 plant species (Table 1). Surprisingly, the gene numbers did not correlate well with the developmental complexity, growth habit, or even evolutionary distance between species. The number of *FBX* loci also showed no correlation with genome size with some of the smaller plant genomes having larger numbers (*e.g.*, *A. lyrata* (Figure 2A)). Clearly, the unicellular alga *C. reinhardtii* has the lowest number of *FBX* loci (88), implying that expansion of the superfamily was connected in part to the evolution of multi-cellularity and/or terrestrial life. However, *S. moellendorffii*, a seedless vascular plant, contains substantially more *FBX* genes than the seed plants, *C. papaya*, *Cucumis sativus* (cucumber), and *Ricinus communis* (castor bean) (544 versus 198/207/255), suggesting that increasing *FBX* gene numbers are not related to land plant evolution nor the appearance of more sophisticated seed plants. Similarly, two annual herbaceous plants, *C. sativus* and *R. communis*, contain lower numbers of *FBX* genes than the perennial woody plant *P. trichocarpa* (207/250 versus 425), which does not support the hypothesis that annual species contain more *FBX* loci than perennial species [19]. Perennial herbaceous plants could have less (903 in *Mimulus guttatus* or monkey flower) or more (1350 in *A. lyrata*) *FBX* genes than annual herbaceous plants (971 in *O. sativa* and 998 in *Brachypodium distachyon* or purple false brome). Even closely related species such as *Zea mays* (corn)/*Sorghum bicolor* (split ∼12 million-years ago (Mya) [29]), *Glycine max* (soybean)/*Medicago truncatula* (barrel medic) (split ∼50 Mya [30]), and *A. thaliana/A. lyrata* (split ∼5 Mya [31]) have widely different gene numbers (417 versus 817, 702 versus 1148, and 897 versus 1350, respectively), indicating that *FBX* gene birth/death has been rapid.

### Evidence for Rapid Lineage/Species-Specific Birth/Death of *FBX* Genes

**Figure 3:**
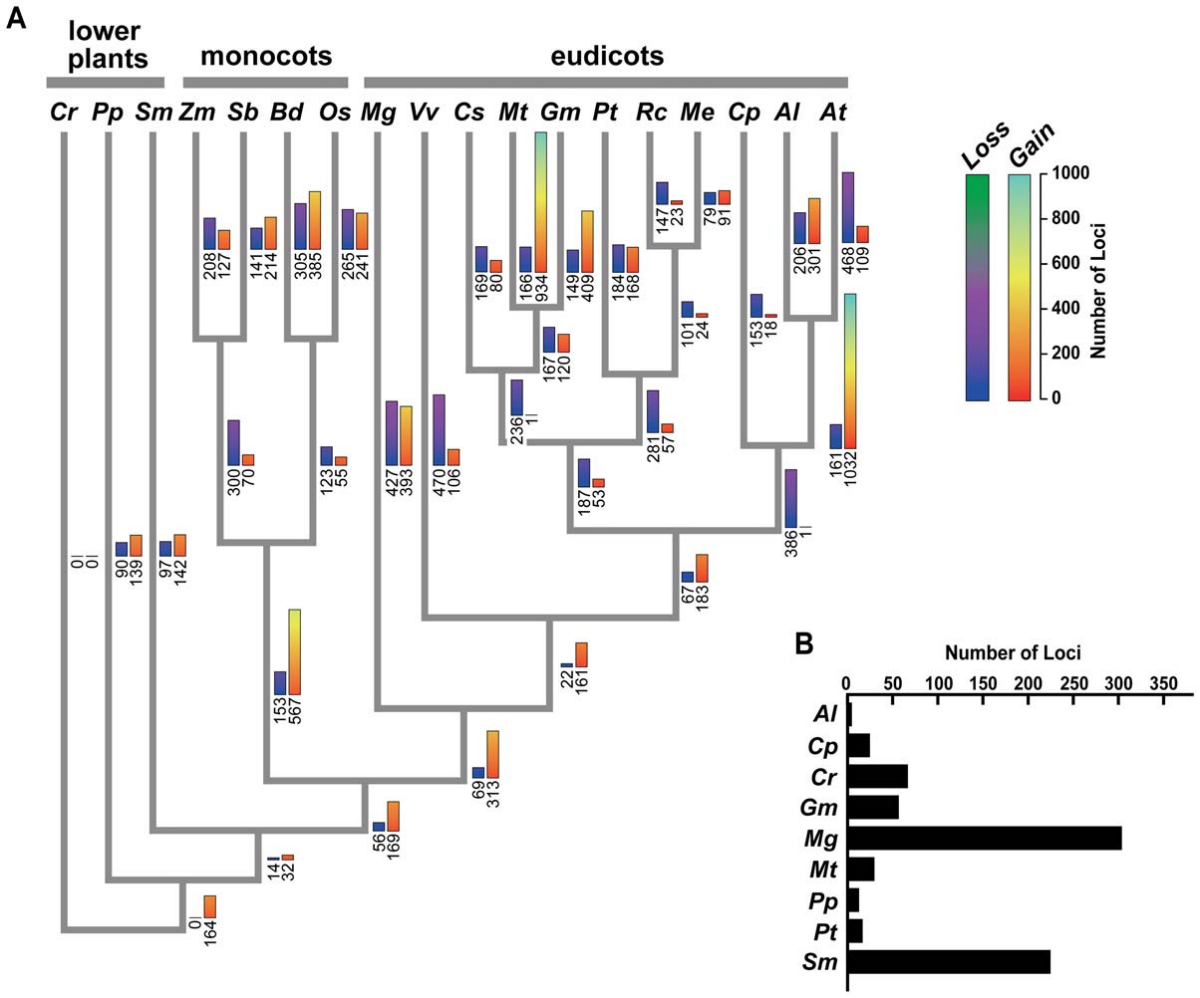
The gain/loss analysis of *FBX* genes during the evolution of 18 plant species. (A) The gain and loss of *FBX* genes in each species and species split nodes. The phylogeny of each species and the taxonomic group designations were adopted from the Angiosperm Phylogeny Website (http://www.mobot.org/MOBOT/research/APweb/). The heatmap color bars at each branch/node represent the predicted number of genes gained (right) or lost (left), respectively. The actual numbers of gains/losses are indicated below each bar. The full names of the species along with their abbreviations are as listed in Table 1. (B) Species-specific generation of *FBX* genes in each species.

To determine if both birth and death occurred in the *FBX* superfamily among the 18 plant species, we built phylogenetic trees based on the FBX protein sequences. Because of the size and complexity of the entire 10,811-member collection, we first grouped the sequences into 351 clusters using MCL [32] from the all-against-all BLASTp search result (E-value <1e-5) [27]. Here, full-length protein sequences were used instead of just the ∼40-amino-acid FBXD to improve accuracy. Forty-seven FBX clusters assembled with 734 total sequences contained proteins from only a single species. These unique sequences likely reflect birth of new *FBX* genes in that species and were removed from the phylogenetic analysis (Figure 3B). Maximum likelihood trees were generated with each of the remaining clusters and the estimates for *FBX* gene birth and death along each branch of the 18 species phylogeny were inferred based on reconciliation of the gene and species trees (see Materials and Methods).

Based on the tree-reconciled results, we detected several independent and dramatic *FBX* gene birth/death events during plant evolution. We saw a net positive gain of *FBX* loci during the steady expansion of the superfamily early in land plant evolution (Figure 3A). In multiple other branches, there were high gene birth rates accompanied by low death rates. Examples include the appearance of the *Arabidopsis* genus (161 losses and 1032 gains), and the possible speciation of *M. truncatula* (166 losses and 934 gains) (Figure 3A). However, net gain was not the predominant pattern in the recent history (<450 Mya) of *FBX* gene evolution. In nine of the 15 lineages leading to extant flowering plant species (*A. thaliana*, *C. papaya*, *C. sativus*, *M. guttatus*, *O. sativa*, *P. trichocarpa*, *R. communis*, *V. vinifera*, and *Z. mays*), there was significantly more gene death relative to gene birth. These patterns together with the finding that *FBX* gene birth and death in general do not correlated with life history traits or evolutionary distance of the species, indicate that the process(es) which caused the dramatic size differences among *FBX* gene superfamilies are highly lineage specific.

### Expansion of *FBX* Genes Correlates with a High Frequency of Gene Inactivation

The large variations in total *FBX* gene numbers and dramatic birth/death in the 18 plant species implied that some loci might be inactive, in part by accumulating deleterious coding region mutations [4]. In accord, we found, while predicting the transcript models of the previously non-annotated *FBX* genes, a substantial number of putative pseudogenes containing frameshifts or premature stop codons. To find the full complement, we retrieved the full-length genomic DNA sequence for each previously annotated *FBX* gene and reexamined by GENEWISE its transcript model. Here, two or three top-scored non-self FBX protein sequences were used as references instead of its own protein sequence to avoid bias. When the two lists were combined, we found surprisingly varied numbers of likely *FBX* pseudogenes in the 18 plant species (Table 1). For example, both *A. lyrata* and *B. distachyon* contain over 300 predicted pseudogenes while *C. sativus*, *Manihot esculenta* (cassava), and *P. patens* contain less than 20. The total numbers of pseudogenes in the 18 plant species correlated poorly with the genome size of the plant but had a strikingly tight correlation with the total number of *FBX* loci (Figure 2B,C). The latter connection implies that the *FBX* superfamily has undergone substantial death in species with high numbers of *FBX* loci.

### Varied Distributions of FBX Protein Subfamilies within the Plant Kingdom

Prior studies with *A. thaliana*, *P. trichocarpa* and *O. sativa* suggested that the *FBX* superfamily has not changed uniformly in plants but with a lineage-specific emphases on particular types of C-terminal recruitment modules (*e.g.*, LRR, kelch repeat, and FBX-associated (FBA)) [15]. To provide further support for such uneven gains/losses, we determined the number of predicted FBX proteins containing the various recruitment modules in each species, using HMMER3 to detect known domains from the PFAM database. If two or more modules were predicted, the FBX protein was sorted according to the combination. Based on the above criterion, we classified the FBX protein collection from the 18 species into 3,269 groups, which either contained no recognizable modules (3,589 total), modules/combinations found rarely (3,678 total), or modules/combinations found significantly more frequently (≥10 representatives, 3,544 total) (Fischer's exact test, *p*<0.05). The latter group encompassed 32 different modules/combinations, including the FBA, kelch repeat, DUF295, FBD, LRR and Tubby+DUF3527 modules (Table S5). Interestingly, the predicted *FBX* pseudogenes were selectively enriched in the group without any predicted recruitment modules (Fischer's exact test, *p*<0.05), suggesting that many of the corresponding proteins lost their ability to bind targets even if expressed (Table S6). Such loss is consistent with the fact that pseudogenes are no longer under selection pressure, with many becoming fragmented as compared to functional genes [3]. In contrast, the remaining *FBX* loci without the hallmarks of pseudogenization were enriched in coding regions for known recruitment modules (Fischer's exact test, *p*<0.05), including 10 well-characterized interaction domains that could participate in target recognition (Table S6).

**Figure 4:**
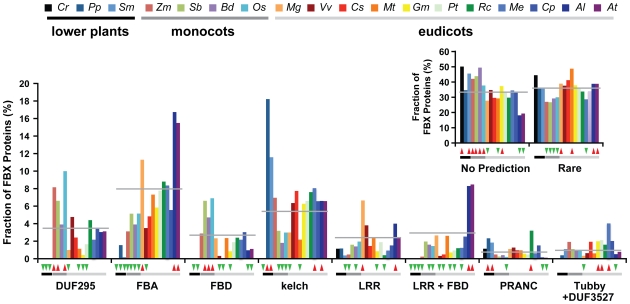
The enrichment/depletion of various C-terminal substrate-recognition modules in the collection of FBX proteins from each species. The red and green-inverted triangles indicate the significant enrichment and depletion of FBX proteins (*p*<0.05, Fisher's exact test), respectively, for each species as compared with the average number (indicated by a horizontal grey line) of FBX proteins containing the same module from all 18 plant species.

When we compared the *FBX* locus numbers in the no recognizable, infrequent and frequent categories among the 18 plant species, we identified numerous species that are selectively enriched/depleted in specific recruitment modules/combinations (Figure 4 and Table S7). As examples, *C. reinhardtii* and the seedless plants *P. patens* and *S. moellendorffii* do not encode FBX proteins with obvious DUF295, FBD, or LRR-FBD modules even though FBX proteins with these modules are common in seed plants. Conversely, *P. patens* and *S. moellendorffii* are enriched in FBX proteins with kelch repeat or PRANC modules. *A. thaliana* and *A. lyrata* are enriched in FBX proteins with LRR+FBD and the FBA modules, whereas most monocots are depleted in these subtypes. Overall, module enrichment/depletion did not agree with the evolutionary tree of the represented plants, in line with the seemingly heterogeneous birth/death rates of the entire *FBX* gene superfamily.

### Evidence for Three Subgroups of *FBX* Genes with Distinct Evolutionary Histories

The functional importance of SCF E3s in plants [17], [18] combined with the heterogeneous *FBX* gene number and module distributions implied that multiple subgroups exist. One group may have been retained during plant (or even eukaryote) evolution and thus likely participates in conserved, possibly essential processes, whereas another could have appeared more recently and likely participates in more species-specific events or is inactive. To help identify members of these two groups, we assigned each *FBX* gene (protein coding and pseudogene) in the 18 species to orthologous groups (OGs) using OrthoMcl [33]. Those *FBX* genes which were not assigned to any OG were defined as “orphan”, while the remaining were grouped into 18 bins depending on whether a homolog could be found in its own genome (potential paralogs with absence of obvious orthologs in the other species, bin 1) or in the 17 other plant species in the collection (potential orthologs, bins 2–18).

**Figure 5:**
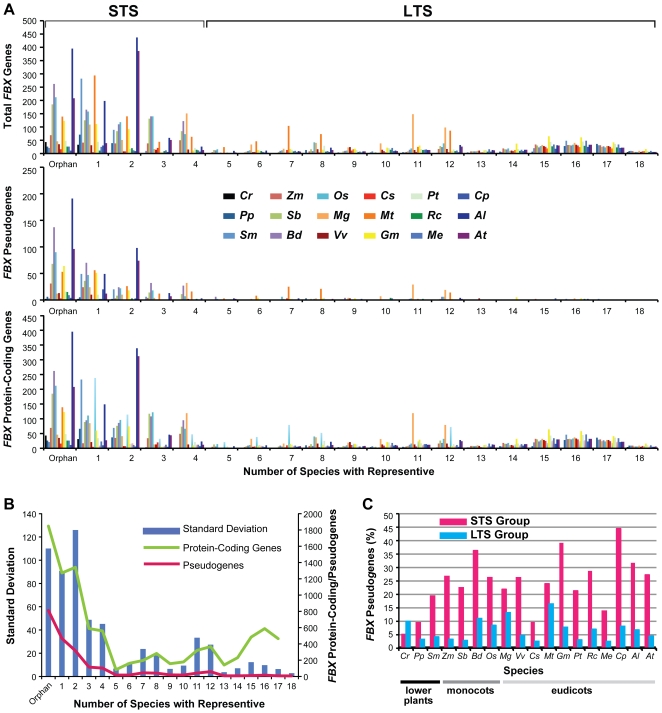
The divergent distribution of *FBX* gene numbers at different conservation levels. (A) The numbers of total *FBX* genes (top panel), *FBX* pseudogenes (middle panel), and *FBX* protein-coding genes (bottom panel) from each species were plotted against the number of species represented in the *FBX* orthologous groups (OGs). “Orphan” indicates the genes without any orthologs. “1–18” denotes the numbers of species in an OG. STS, small taxonomic scale; LTS, large taxonomic scale. (B) The distribution of standard deviations of *FBX* gene numbers, total protein-coding *FBX* gene numbers, and total *FBX* pseudogenes from 17 plant species (*Cr* is not included) at different conservation levels. (C) The percentage of *FBX* pseudogenes with low (STS) or high (LTS) conservation levels in each of the 18 plant species.

As can be seen in Figure 5A,B, two separate OG collections emerged depending on the number of species included. The larger collection included the orphan genes, numerous loci in bin 1 which appear to be species specific, and loci in bins 2–4 which have orthologs in only 1–3 of the 17 other species. We designated this collection as **s**mall **t**axonomic **s**cale (STS) to reflect their limited distributions, with the logical inference that they are either inactive loci or perform functions limited to few plant species. In fact, most of the pseudogenes are in the STS collection (Figure 5C). The confinement of most *FBX* pseudogenes to a single or few species implies that they either were formed relatively recently or represent ancient loci that have diverged by neutral change beyond recognition. Whereas land plants have 18–60% of their *FBX* genes in the STS collection, *C. reinhardtii* has a substantially higher percentage (84% of total). This increase may reflect methodological issues in generating OG relationships with such a divergent plant species, or the possibility that many of the *C. reinhardtii FBX* loci have either algal-specific functions, have functions in common within land plants but have significantly diverged sequences, or have diverged enough to become inactive.

The second smaller OG collection included members in bins 5–18, which we have designated **l**arge **t**axonomic **s**cale (LTS) to reflect their wider distributions among the plants. The LTS collection has fewer predicted pseudogenes as compared to the STS collection and three potentially distinct subsets of protein-coding genes (Figure 5A,B). The largest subset includes members in bins 14–18, which based on their broad distribution, likely encode highly conserved and ancient FBX proteins that perform tasks essential to all plants, or even to eukaryotes in general. In support, a number of well-characterized FBX proteins from *A. thaliana* are in these bins, including TIR1, COI1, SLY1, LKP2, FKF1, ZTL, and EBF1/2 (Table S3). Two more restricted subsets, centered at bins 7–8 and 10–12, include *FBX* genes with partially limited phylogenetic distributions. By checking the OGs from these more restricted bins, we found that the species represented were not limited to eudicots, monocots, or lower plants separately but in various combinations, suggesting that their emergence was not caused by species sampling biases (*e.g.*, 11 eudicots versus 4 monocots). For example, analysis of all 34 OGs centered at bins 10–12, revealed only 5 that comprised a *FBX* gene subgroup which was enriched in eudicots (10 of the 11 species) but absent in the monocots and lower plant representatives. The remaining 29 OGs contained species in various combinations, suggesting they experienced independent lineage-specific losses. The only notable exception to the LTS/STS grouping was *C. reinhardtii*, which was calculated to have more pseudogenes in its LTS collection on a percentage basis than STS. We do not consider this deviation significant given the fact that this alga has few total *FBX* loci (88), and very few pseudogenes (5) and members of the LTS collection (9) (Table 1).

Based on this OG analysis, we divided the *FBX* loci into three broad groups, pseudogenes, and protein-coding genes in the STS (STSP) and LTS (LTSP) groups. We then examined separately their evolutionary selection (purifying or relaxed), mechanism of duplication (*e.g.*, segmental versus tandem duplications), and their expression levels/patterns, which are all potential indicators of functionality and evolutionary history.

### Differential Selection Pressures on the LTSP, STSP and *FBX* Pseudogene Loci

**Figure 6:**
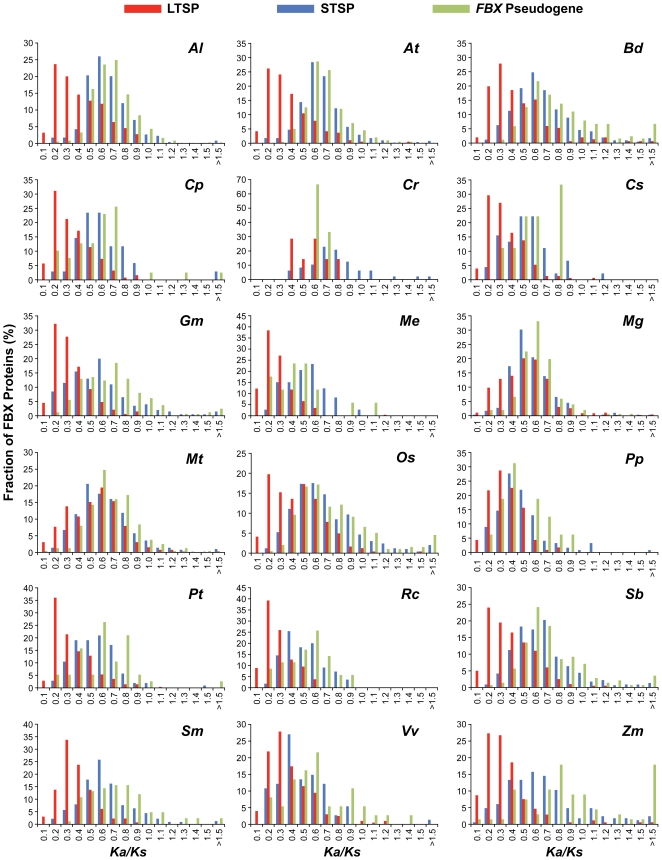
The distributions of *Ka/Ks* values for the LTSP, STSP and *FBX* pseudogenes in each of 18 plant species. The *Ka/Ks* value for each full-length sequence was calculated by comparing it to the MRCA transcript sequence using the method of Goldman and Yang [34].

The ratio of non-synonymous (*Ka*) to synonymous (*Ks*) nucleotide substitution rates allows assessing the nature of gene selection in which *Ka/Ks* values well below 1 are indicative of functional genes under purifying selection, whereas *Ka/Ks* values approaching 1 are indicative of non-functional genes experiencing relaxed selection and pseudogenes undergoing neutral change. Using the *Ka/Ks* estimation method of Goldman and Yang [34], we found that the LTSP group of *FBX* loci is enriched for genes undergoing stronger purifying selection whereas the STSP and pseudogene groups are enriched for loci experiencing more relaxed selection or neutral change. This difference between LTSP and STSP/pseudogenes, as observed in the frequency distributions of the *Ka/Ks* values (Figure 6, Table S8), was obvious for almost all plant species tested with significant purifying selection evident for the LTSP genes in 16 of the 18 species (Wilcoxon rank sum test, *p*<0.001). The differences in frequency distributions were statistically less significant for the remaining two species, *C. reinhardtii* and *P. patens*, even though a slight separation of the *Ka/Ks* values could be seen between the LTSP and STSP/pseudogene groups (Figure 6, Table S8).

**Figure 7:**
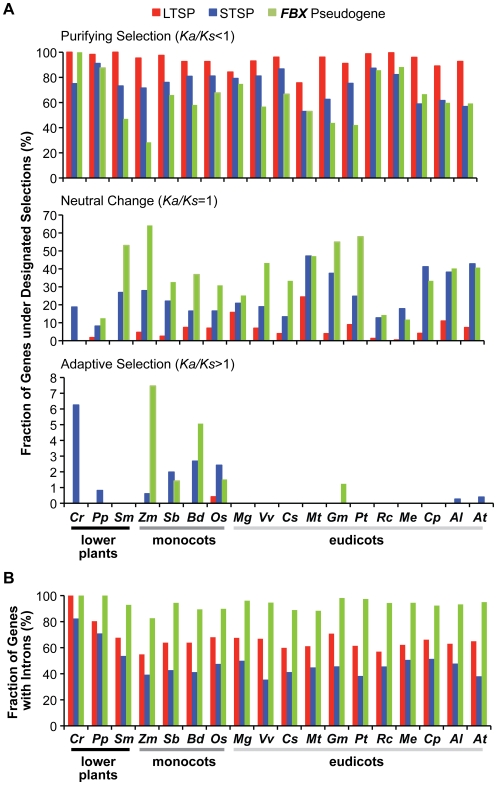
Comparisons of evolutionary selections and intronization among the LTSP, STSP and *FBX* pseudogene loci in each species. (A) The percentage of *FBX* genes under purifying selection (top panel), neutral change (middle panel), and adaptive selection (bottom panel) in each subgroup. (B) The percentage of *FBX* genes containing at least one intron in each subgroup.

The overlapping *Ka/Ks* profiles of STSP and *FBX* pseudogene groups predicted that a substantial number of STSP genes experienced the same neutral change constraints as *FBX* pseudogenes. To further validate this scenario, we applied the *Ka/Ks* ratio test of Nekrutenko *et al.* [35] to compare the LTSP, STSP and *FBX* pseudogene groups for the enrichment of loci with more purifying or more relaxed/neutral *Ka/Ks* values (at 5% significance level), respectively. As seen in Figure 7A, both the STSP and *FBX* pseudogene groups had lower percentages of genes with purifying *Ka/Ks* values but contained much higher percentages of genes with relaxed/neutral *Ka/Ks* values as compared to the LTSP group for almost all 18 species. Some species displayed especially strong differences with few LTSP genes containing neutral values (*e.g.*, *S. moellendorffii*, *R. communis*, and *M. esculenta*). *M. guttatus* was the only exception; here, the percentages of genes with neutral *Ka/Ks* values were near equal for the LTSP and STSP/pseudogene groups (Figure 7A). It is also noteworthy that we detected a small collection of mainly STSP and predicted *FBX* pseudogenes with *Ka/Ks* values exceeding 1.4, which could be indicative of adaptive selection (Figure 6). Most of these loci were from *C. reinhardtii* and the sampled monocot species (*e.g.*, *Z. mays* (Figure 7A)). The reason(s) behind this anomaly are unknown but could imply that some monocot *FBX* loci are under strong positive selection, *i.e.*, accumulating non-synonymous substitutions faster than synonymous substitutions.

As an alternative, we used the method of Nei and Gojobori [36] to calculate *Ka/Ks* values, which applies p-distance for sequence distance calculations. Similar differences in *Ka/Ks* frequency distributions were observed for the LTSP, STSP and *FBX* pseudogenes among the 18 species (Table S9), further supporting the different selection pressures among the three groups. The *Ka/Ks* comparisons discussed above were based on the complete *FBX* gene. To test whether the *FBXDs* and the remainder of the *FBX* coding regions (Δ*FBXDs*) might be under different selection pressures, we also compared the *Ka/Ks* distributions for the *FBXD* and Δ*FBXD* regions separately. Like the full-length *FBX Ka/Ks* distributions, those for the *FBXD* and Δ*FBXD* sequences differed between the LTSP and STSP/pseudogene groups, suggesting that the FBXD and recruitment modules were under similar purifying and relaxed/neutral change pressures, respectively. The differences between the groups were less for the *FBXD* comparisons, likely reflecting the fact that the FBXDs must retain their interactions with a limited set of SKP1 proteins to remain functional (Figures S3, S4, and Table S9). Taken together, it appears that the LTSP genes are under stronger purifying selection while STSP genes and *FBX* pseudogenes are under more relaxed selection. The relatively reduced selection constraints of STSP members are consistent with a lesser functional importance and suggest that many are progressing toward becoming pseudogenes.

### STSP and *FBX* Pseudogenes Used Similar Amplification Mechanisms

Further evidence that STSP and *FBX* pseudogenes are evolutionarily related was provided by examining their amplification patterns and chromosomal locations. Previous studies with *A. thaliana* and *O. sativa* suggested that a large number of *FBX* genes evolved recently by tandem duplications [12], [15], [19]. To examine if this mechanism was used more universally, we determined by DAGchainer [37] the number of LTSP, STSP and *FBX* pseudogene loci among 17 of the plant species which were derived from tandem or segmental duplications (File S7). (*C. papaya* was excluded due to the absence of gene position information at the time of the analysis). Segmental duplications included those events generated via whole genome amplifications (polyploidy) as well as the duplication of smaller chromosomal segments, while tandem duplications were defined as events that generated paralogous genes in proximal genomic regions [11]. The remaining loci were either singletons or duplicates of unclear origins (*e.g.*, retrotransposition).

**Figure 8:**
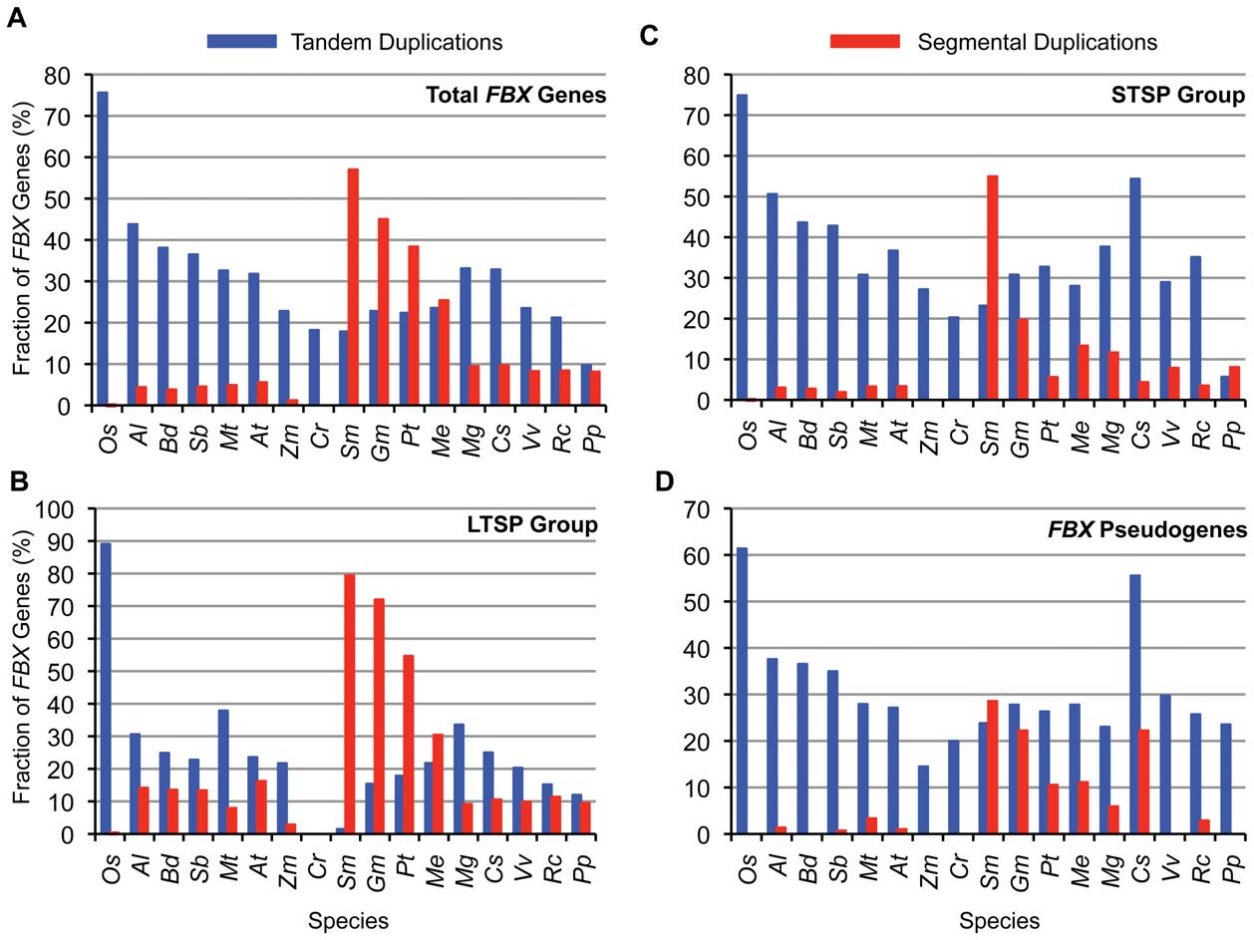
The distributions of tandem and segmental duplicated *FBX* genes in each of 18 plant species. (A) Total *FBX* genes. (B) STSP genes. (C) LTSP genes. (D) *FBX* pseudogenes.

Based on the relative contributions of tandem versus segmental duplications to the *FBX* gene repertoire, we found three different *FBX* gene expansion patterns (Figure 8A). One pattern, evident in *A. thaliana*, *A. lyrata*, *B. distachyon*, *C. reinhardtii*, *M. truncatula*, *O. sativa*, *S. bicolor*, and *Z. mays*, predicted that tandem duplications played the main role while segmental duplication played only a minor role during the *FBX* gene expansion. The most obvious examples are *C. reinhardtii and O. sativa* where none of their entire collection of *FBX* loci arose from segmental duplications while most arose from tandem duplications. The second pattern, evident in *S. moellendorffii*, *G. max*, *P. trichocarpa*, and *M. esculenta*, predicted that both segmental and tandem duplications contributed significantly to *FBX* gene expansion with segmental events playing the dominant role in forming the LTSP group (Figure 8A–C). The third pattern was intermediate of the first two and was exemplified by *C. sativus*, *M. guttatus*, *P. patens*, *R. communis*, and *V. vinifera*. None of these three patterns was confined to a particular plant group (algae, lower plants, eudicots and monocots), implying that the relative importance of duplication mechanisms, like other traits of *FBX* loci, differed among the plant lineages.

When analyzing the duplication patterns of the LTSP, STSP and *FBX* pseudogene groups separately, it became obvious that the STSP and *FBX* pseudogene loci relied more heavily on tandem duplications for their birth as compared to the LTSP group (Figure 8B–D). Species with especially strong bias for tandem versus segmental events include *B. distachyon*, *C. reinhardtii*, *O. sativa*, *P. patens*, *V. vinifera and Z. mays*, which have very few or no STSP/pseudogene loci potentially generated by segmental events (Figure 8D)). Pseudogenes are typically more enriched relative to active genes in the heterochromatic and generally transcriptionally silent regions near centromeres [38]. Using *A. thaliana* as an example, we accordingly found that the distributions of STSP and *FBX* pseudogene members are more clustered and extend closer to the centromeres than are LTSP genes, especially for chromosomes 2, 3 and 5 (Figure S5).

We also examined whether the sizes of the STSP and LTSP groups correlated with genome size by a Spearman rank correlation coefficient analysis of all the 18 plant species. No significant correlations were observed (LTSP, *rho* = 0.3, *p* = 0.3 and STSP, *rho* = −0.1, *p* = 0.7). In contrast to a previous hypothesis that herbaceous annual plants contain more *FBX* genes than woody perennial plants [19], we detected no such connection. Using the two-sided Kolmogorov-Smirnov test, we found that the plants analyzed here (Table S1) did not cluster significantly based on growth habit when either the total *FBX* (*p* = 0.1), LTSP (*p* = 0.6), STSP (*p* = 0.04), or *FBX* pseudogene (*p* = 0.04) groups were examined separately.

The *FBX* pseudogenes could have arisen from gene duplication events followed by inactivation or by retrotransposition using *FBX* mRNAs as templates, which would generate *a priori* pseudogenes without introns or native promoters. Analysis of human pseudogene birth found that both mechanisms were involved with the paucity of obvious introns implying that retrotransposition played the dominant role (*e.g.*, [39]). For the 18 plant species tested here, we found the opposite effect, that the exonic regions of most *FBX* pseudogenes were interrupted by intron-like sequences. In fact, a greater percentage of *FBX* pseudogenes were affected by intron-like sequences and contained higher numbers of these inserts than both LTSP and STSP genes (Figure 7B). These intervening sequences could represent ancestral introns, thus arguing against a role for retrotransposition in *FBX* pseudogene expansion. Alternatively, some intron-containing pseudogenes may still have arisen by retrotransposition with the intron-free coding regions then frequently acquiring intron-like insertions during their initial inactivation and/or continued degeneration. The fact that members of the STSP group, which could represent non-functional loci in the process of pseudogenization, have comparably fewer introns would support the latter scenario.

### A Substantial Number of STSP and *FBX* Pseudogene Loci are Predicted to be Less or Non-Functional

To test whether a substantial number of STSP loci are less or non-functional, we compared the expression patterns of members from LTSP, STSP and *FBX* pseudogene groups in each plant species, using the availability of expressed sequence tags (ESTs) to estimate transcriptional activity. In 15 of the 18 species, significantly higher numbers of expressed *FBX* genes were from the LTSP group (Fischer's exact test, *p*<0.001, Table 2). Conversely, for 14 of the 18 species, loci with no evidence of expression were statistically enriched in the STSP and *FBX* pseudogene groups (*p*<0.001, Table 2), suggesting that many of the loci in these two groups are transcriptionally inactive and thus not likely functional.

**Table 2:** Comparison of *FBX* gene expression among LTSP genes, STSP genes, and *FBX* pseudogenes.

Species^1^	LTSP		STSP		*FBX* Pseudogene		Fisher's exact test		
	W_est	Wo_est	W_est	Wo_est	W_est	Wo_est	LTSP>STSP	LTSP>Pseudo	STSP>Pseudo
*Al*	116	103	49	712	10	360	<2.2E-16*	<2.2E-16*	4.4E-03
*At*	161	30	172	335	42	157	<2.2E-16*	<2.2E-16*	4.7E-04*
*Bd*	140	37	218	291	107	205	<2.2E-16*	<2.2E-16*	9.1E-03
*Cp*	54	70	4	31	2	37	2.5E-04*	1.9E-06*	2.9E-01
*Cr*	1	8	13	61	1	4	8.3E-01	8.9E-01	7.9E-01
*Cs*	17	135	1	45	0	9	4.8E-02	3.6E-01	8.4E-01
*Gm*	273	59	39	169	27	135	<2.2E-16*	<2.2E-16*	3.5E-01
*Me*	76	154	6	69	2	16	4.9E-06*	4.1E-02	8.2E-01
*Mg*	135	324	37	255	11	141	3.6E-08*	1.6E-09*	5.3E-02
*Mt*	134	257	68	449	14	226	3.5E-14*	<2.2E-16*	1.3E-03
*Os*	183	64	204	313	42	165	<2.2E-16*	<2.2E-16*	3.5E-07*
*Pp*	73	44	60	64	7	10	2.0E-02	8.2E-02	3.8E-01
*Pt*	95	185	14	93	2	36	1.8E-05*	7.2E-05*	1.5E-01
*Rc*	69	89	2	55	4	31	1.1E-09*	1.8E-04*	9.7E-01
*Sb*	107	95	123	349	14	129	2.4E-11*	<2.2E-16*	1.2E-05*
*Sm*	63	73	66	258	8	76	3.0E-08*	2.9E-09*	1.3E-02
*Vv*	95	107	15	61	7	30	1.8E-05*	9.7E-04*	5.7E-01
*Zm*	166	9	121	52	28	41	2.8E-10*	<2.2E-16*	2.4E-05*

*p<0.001.

1 See Table 1 for species abbreviations.

As a further test of this hypothesis, we reviewed the literature for available genetic and biochemical data on *FBX* genes in *A. thaliana*, whose genome is the best interrogated in plants, and compared the results generated for the LTSP, STSP and *FBX* pseudogene loci. To date, the functions of 47 *A. thaliana FBX* genes have been characterized; most come from the LTSP group (39 of the 191 members) with few from the STSP group (8 of 507 members) and none from the *FBX* pseudogene group (0 of 199 members) (Tables 2 and S3). Because functional studies might be biased by researcher's interests, we also examined the collection of *A. thaliana* FBX proteins basically chosen at random for biochemical studies, including representatives tested for SKP1/ASK interaction (*e.g*., [12], [20]) (Table S4). Of the 72 FBX proteins confirmed to associate with SKP1/ASK proteins, 45 were from the LTSP group, 16 were from the STSP group, and none were from the *FBX* pseudogene group, again a significant enrichment for LTSP members (Fisher's exact test, *p*<2.2e-16). The substantial enrichment of expressed and functional *FBX* loci in the LTSP group further supports the notion that they are more functionally important than members of STSP and *FBX* pseudogene groups.

### Lineage/Species-Specifically Expanded *FBX* Genes are not Significantly Linked to Stress/Pathogen Responses in *A. thaliana*

Previous work proposed that a subfamily of *A. thaliana FBX* genes (mostly from STSP and *FBX* pseudogene groups base on our analysis (Figure 5)) evolved by positive selection (*Ka/Ks* >1), with variations in a collection of amino acids in the recruitment module potentially playing an important role in innate immunity and stress responses [14]. However, our data imply that most STSP and *FBX* pseudogene members are under relaxed selection or neutral change and not under positive selection (Figures 6, 7, S3, and S4; Tables S8 and S9).

**Figure 9:**
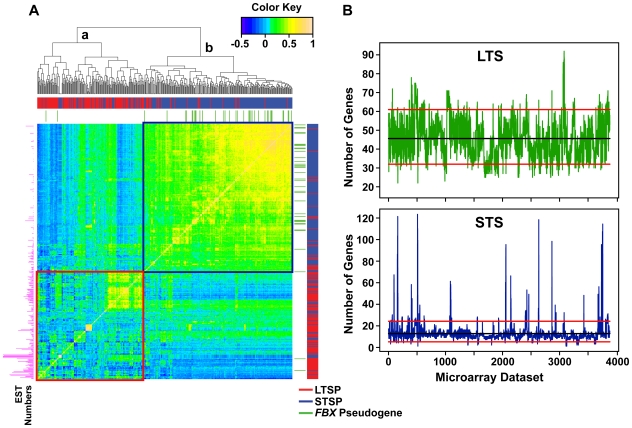
Expression correlation test and functional predictions of *A. thaliana FBX* genes. (A) The expression correlations of 395 *FBX* genes. The Pearson's correlation coefficients of ∼4,000 microarray datasets for each *FBX* gene were calculated pairwise. The dendogram at the top of the panel shows the hierarchical clustering of 395 *FBX* genes based on the dissimilarities of Pearson's correlation coefficients. The bar codes indicated the distributions of LTSP *FBX* genes (red color), STSP *FBX* genes (blue color), and *FBX* pseudogenes (green color). The horizontal histogram (magenta color) on the left shows the EST numbers for each *FBX* gene. The heatmap color key, indicating the Pearson's correlation coefficient values, is shown on the right top corner of the panel. The correlations of cluster-a genes are highlighted with a red box and the correlations of cluster-b genes are highlighted with a blue box. (B) Enrichment assay of expressed LTS (top panel) and STS (bottom panel) *FBX* genes in each of the 3,868 different microarray datasets. The black line in each panel indicates the mean numbers of expressed *FBX* genes. Experiments above the top or below the bottom red line in each panel represent the datasets which significantly increased or decreased overall *FBX* gene expression frequency, respectively (Fisher's exact test, *p*<0.05). The statistically significant enrichment of expressed *FBX* genes in a microarray dataset, which could infer the function(s) of *FBX* genes, and the experimental condition examined by the microarray for both expressed LTS and STS *FBX* genes are summarized in Table S10.

To explore a possible connection between *A. thaliana* STSP and *FBX* pseudogenes and the stress/pathogen responses, we examined the expression correlations of all *A. thaliana FBX* genes in the full compilation of expression microarray datasets available at NASCArrays. These experiments integrated 396 of the 897 potential *FBX* loci, 146 from LTSP group, 213 from STSP group, and 37 from the *FBX* pseudogene group. Interestingly, pairwise correlations revealed two large clusters with related expression profiles (Figure 9A). One displayed divergent expression patterns and was strikingly enriched for LTSP members (cluster a, average Pearson's correlation coefficient  = 0.1), in agreement with their predicted or known roles in numerous unrelated processes in plants (Figure 9B and Table S10). In contrast, a second cluster displayed highly correlated co-expression and was significantly enriched in STSP and *FBX* pseudogene members (cluster b, average Pearson's correlation coefficient  = 0.4). To eliminate the possibility that the tight correlation in cluster b was caused by low expression of its members, we further compared the expression correlations between the cluster b *FBX* genes and 28 non-plant internal controls (median microarray value ≤ median microarray value of cluster b *FBX* genes). If random weak signals above noise generated the tight correlations, the internal controls would have been intermingled with cluster b *FBX* genes. Instead, clear differential correlation patterns between these two group genes were resolved (Figure S6), suggesting that the transcriptional patterns of cluster b *FBX* genes might be equally unregulated or regulated similarly.

When comparing the expression enrichment of LTSP versus STSP members in *A. thaliana*, the expression patterns of STSP members did not specifically correlate with exposure to almost all stresses tested (heat, cold, oxidative, nitrogen/carbon/potassium starvation, salinity, heavy metals, high light, wounding, *etc*,), programmed cell death, or with invasion by a number of pathogens (Figure 9B and Table S10). In contrast, the best specific correlations of STSP *FBX* loci to gene expression were for microarray examinations of embryo and pollen development and the response to phosphate starvation (Table 3). Collectively, the expression studies failed to detect a connection between STSP and *FBX* pseudogenes and plant stress/pathogen defense.

**Table 3:** The only three microarray datasets showing the higher enrichment of expressed STS *FBX* genes than that of LTS *FBX* genes from 3,868 microarray datasets (*p*<0.05).

Group	Slide_name	Number of genes	*p*-value	Experiment ID	Category
LTS	Lindsey_1-1_globular-apical_Rep1_ATH1	|||||||||||||||||||||| 45/104	2.7E-02	55	development_embryo
STS	Lindsey_1-1_globular-apical_Rep1_ATH1	||||||||||||||||||||||||| 50/193			
LTS	Broadley_1-2_A2-Bo-P-phosphate-starved_Rep1_ATH1	||||||||||||||||||||||||| 51/98	4.5E-02	121	nutrient _phosphate
STS	Broadley_1-2_A2-Bo-P-phosphate-starved_Rep1_ATH1	|||||||||||||||||||||||||||||| 61/182			
LTS	Honys_BCP1_SLD	|||||||||||||||||||||| 44/105	4.7E-02	48	development _pollen
STS	Honys_BCP1_SLD	||||||||||||||||||||||||| 51/192			

*p*-value was calculated by comparing pairwise the expressed gene numbers of LTS and STS genes using Fisher's exact test. The numerator shows the expressed gene numbers and the denominator shows the non-expressed gene numbers in each group. The bar codes display the numbers of expressed genes. The title for each experiment is shown in Table S10.

## Discussion

Plant genomes have undergone a wide array of molecular events that have enlarged or contracted their sizes, which in turn have diversified the size and complexity of various multi-gene families. Despite extensive gene duplication events, the great majority of plant gene families contain only a handful of members [11]. At the other extreme, a small number of gene families have expanded considerably to now include several hundreds to even >1,000 members [7], [12], [40]. Among the significantly expanded families, the *FBX* superfamily has arguably experienced the most dramatic changes in which a large number of lineage/species-specific gains and losses have occurred as land plants evolved from single-cell aquatic species to advanced angiosperms. These dramatic changes in *FBX* gene family content even occurred among closely related species. As illustrations, *A. thaliana* and *A. lyrata* which diverged only 5 Mya, differ by 453 *FBX* loci, with *A. thaliana* acquiring 109 loci and losing 468 loci as compared to their MRCA. *G. max* and *M. truncatula*, which split ∼50 Mya differ by 446 *FBX* genes with *M. truncatula* acquiring 934 loci and losing 166 loci since the divergence of these two species. Similarly high rates of *FBX* gene birth and death were also observed by Thomas [14] in the nematodes *Caenorhabditis elegans*, *C. briggsae* and *C. remanei*, which were estimated to have diverged between ∼20–50 [41] and 100 Mya [42], suggesting that such rapid gene gains and losses may be a common feature of this superfamily.

Key to our approach was the development of a pipeline to comprehensively search plant genomes for all *FBX* loci, using a deep list of FBXD sequences as the initial queries, which was followed up by a second search with a large collection of previously annotated *FBX* gene sequences. This strategy identified numerous *FBX* loci that were not yet annotated, which concomitantly expanded the *FBX* gene superfamilies substantially over those previously reported. For example, the *A. thaliana FBX* superfamily increased from 694 [12] to 897 loci, the *O. sativa* superfamily increased from 779 [15] to 971 loci, and the *P. trichocarpa* superfamily increased from 337 [15] to 425 loci, as compared to the previous most complete annotations. The depth of the collection was supported by subsequent analysis of the *A. thaliana* superfamily. Here, we found that all previously described FBX proteins, either studied genetically or by interaction assays with SKP1, were in our complete list (Tables S3 and S4), as opposed to prior studies where some notable representatives were missing (*e.g*., COI1 [19]).

Our analyses revealed that several search parameters can significantly affect *FBX* gene predictions. First, the detection of FBXD sequences using HMMER is sensitive to the E-value cutoff (Tables S3 and S4). Our observations that all SKP1-interacting FBX proteins from *A. thaliana* contain an FBXD with an E-value cutoff ≤1 (Table S4), while more stringent E-value cutoffs (≤0.01) exclude some well-known FBX proteins (*e.g.*, *A. thaliana* AFB4/5 [43], MAX2/ORE9 [44], [45], and COI1 [24], [46]), demonstrate that an E-value cutoff near 1 may be necessary to avoid false predictions but still generate a comprehensive collection. The use of a HMMER E-value cutoff of ≤0.01 by Yang *et al.* [19] may explain why they reported fewer *FBX* genes in *A thaliana*, *O. sativa* and *P. trichocarpa* (660, 680 and 320, respectively) and missed functionally important FBX proteins in *A. thaliana* as compared to our study. Conversely, Xu *et al.* [15] used an E-value cutoff ≤10, which could have falsely identified some loci.

Second, we found that the genomic coding region of most *FBX* genes is larger than 1 kbp (Figure S1). Consequently, using just 1-kbp genomic regions as done previously [14], likely predict poor transcript models with high frequency. Here, we retrieved a ∼10-kbp region for each locus, based on the likely upper size limit of most *FBX* loci, to help ensure that the most complete transcript sequences would be annotated by sequence similarity-based GENEWISE predictions. Third, because many *FBX* genes arose by tandem duplications, the building of both transcript models and coding sequence annotations by GENEWISE can be challenged by adjacent *FBX* gene sequences, which will induce GENEWISE to overlook the neighboring *FBX* loci. The CTT algorithm developed in this work helped overcome this hurdle by allowing us to find and separate these tandem *FBX* genes by iterative trimming of a 10-kbp window. Our discovery of a significant number of previously non-annotated *FBX* loci by CTT suggests that it could help the comprehensive annotation of other superfamilies enriched in tandem duplicates.

Domain analysis of the FBX protein collection detected a wide array of C-terminal recruitment modules linked to the N-terminal FBXD. Asymmetric partitioning of the various modules among the different plant lineages was surprisingly variable (*e.g.*, *C. reinhardtii* and the lower plants *P. patens* and *S. moellendorffii* lack FBX proteins with the DUF295 domain and the LRR-FBD modules). Such differential enrichments/depletions could reflect the targeting of lineage/species-specific substrates or convergent evolution in which the various plant lineages adopted different substrate recruitment modules in FBX proteins to recognize the same substrate. Examples of the former could include exogenous proteins derived from pathogens or endogenous proteins required for self-recognition during pollination, which has been shown in *Solanaceae*, *Plantaginaceae*, and *Rosaceae* species to involve polymorphic collections of FBX proteins [47], [48]. As an aside, possible convergent evolution could challenge attempts to predict target/E3 interactions based on the paired arrangements known in other species.

Based on the evolutionary history and species distributions, we divided the *FBX* superfamily into three groups, LTSP, STSP and *FBX* pseudogene. It is likely that the LTSP and the STSP/pseudogene collections correspond to the more “stable/conserved” and more “unstable/divergent” clusters described by Thomas [14] and Xu *et al.* [15], respectively, from more limited analyses of plant *FBX* genes. The wide distribution of LSTP group members implies that they direct essential ubiquitylation processes in plants. In accord, the LTSP group has experienced the strongest purifying selection among the three groups, shows the highest and most diverse expression patterns, and includes most *A. thaliana FBX* loci previously demonstrated to control key processes in plant biology (Tables S3 and S4). One collection of LTSP genes in particular was found in most, if not all, land plants (OG bins 14–18 Figure 5) with relatively similar numbers present in each of the 17 land plant species examined (ranging from 81 to 211 members, median  = 105±32). We predict that these FBX proteins assemble SCF E3 complexes critical to a core set of ancient ubiquitylation events. In this core set, only a handful has demonstrated functions (*e.g.*, TIR1/ABF1-5, COI1, EBF1/2, FBL17, SLY1, and ZTL (Table S3)). Consequently, focused reverse genetic analyses on these widely distributed, but as yet uncharacterized, LTSP members will likely provide important insights into other processes common in land plants that rely on ubiquitylation as a key component. Using *A. thaliana* and *O. sativa* as illustrations, this would concentrate genetic efforts to 104 and 107 LTSP loci, instead of the 897 and 971 total *FBX* loci present in each species, respectively.

The expansion of the *FBX* superfamily was significantly correlated with a substantial gain in the number of putative pseudogenes as identified by the presence of frameshifts and premature stop codons, and lack of expression. For example, we predict that *A. lyrata*, *B. distachyon*, and *M. truncatula*, which have some of the largest *FBX* gene superfamilies, have at least 370, 312 and 240 *FBX* pseudogenes, whereas *C. communis*, *P. patens*, and *M. esculenta*, which have some of the smallest *FBX* gene superfamilies, have as few as 9, 17 and 18, respectively. Although the number of *FBX* pseudogenes we identified is likely an underestimate, either because many *FBX* pseudogenes may be too divergent for FBXD detection or appear by sequence comparisons to be intact, our findings are consistent with the global analysis of pseudogenes in *A. thaliana* and *O. sativa* [3]. A previous *FBX* gene phylogeny studies did not account for pseudogenes [15], but noted that a significant number of annotated *A. thaliana FBX* genes contain mutations, which affected the C-terminal ends of the encoded proteins. While they hypothesized that the frameshift defects altered the recruitment modules to potentially increase target diversity for the resulting SCF complex, our findings that many of these frame-shifted genes likely evolved neutrally and are not expressed suggest that they are pseudogenes. Furthermore, the significant enrichment in intron-like insertions and the absence of known target recruitment modules at the C-termini of the encoded proteins predict that few of these *FBX* loci direct the assembly of functional SCF E3s complexes even if translated.

In addition to *FBX*, a number of other plant gene families are significantly overpopulated with pseudogenes (*e.g.*, receptor-like kinases, cytochrome P450 reductases, NBS-LRR, and cellulose synthase [3]). Several pseudogene-rich families have connections to environmental responses with the implication that their rapid turnover is driven by the selection pressure imposed by the environmental conditions. However, several features set the *FBX* superfamily apart from these other pseudogene-rich families, suggesting that the *FBX* superfamily is not under the same evolutionary constraints. First, except for the receptor-like kinases [40], the *FBX* superfamily is much larger. Second, the birth and death rates of *FBX* loci are much more variable than essentially all other plant families including receptor-like kinases [11], [40]. And third, whereas members of many pseudogene-rich families tend to be transcriptionally up-regulated during environmental stress, the *FBX* superfamily is overall not responsive to either biotic or abiotic challenges [49].

Although members of the STSP group do not have obvious premature stop codons or frameshifts, they share several features in common with *FBX* pseudogenes. Like *FBX* pseudogenes, members of the STSP group have experienced more relaxed purifying selection pressures and have weaker and more correlated expression patterns, with the group mostly devoid of *FBX* loci with demonstrated functional importance, implying that many are still transcriptionally active but in the process of pseudogenization. While a prior study suggested that a subgroup of *A. thaliana FBX* genes, which appear related to our STSP group, are important for pathogen defense potentially by degrading pathogen proteins entering the plant host via bacterial type III secretion systems [14], our correlation analysis of STSP expression patterns do not support such a role. However, it could be argued that low-level constitutive expression is what one might expect for *FBX* genes involved in defense, given that the host can predict neither the time nor the place of the infection.

Although more limited in scope, a surprisingly similar evolutionary pattern was predicted for the SKP1-binding partners of FBX proteins in *A. thaliana* and *O. sativa*. In both species, a subset of slowly evolving *SKP1* loci with strong expression and confirmed biological importance were identified along with a more diverse, faster evolving subset with weak or limited expression [50], [51]; attributes remarkably similar to the LTSP and STSP *FBX* gene groups identified here. Such connections suggest that the plant *SKP1* family diversified in concert with the *FBX* superfamily possibly to maintain the necessary expression patterns and protein-protein interactions. Similarly, analysis of the BTB family of substrate recruitment modules in *A*, *thaliana*, *O sativa*, and a few other plant species detected a subfamily of MATH-BTB proteins that underwent substantial expansion in the monocot lineages with evidence for diversifying selection and pseudogenization, suggesting that this E3 family is also under unusual selection pressures [23]. Taken together, these phylogenetic studies suggest that diversification Ub ligases in particular, and the UPS in general, may be strongly encouraged in plants.

Contrary to expectations, we found little correlation in *FBX* gene numbers to the growth habit (herbaceous versus woody), life cycle (annual versus perennial), or evolutionary history of land plants. In fact, when plotted against the lineage tree of the 18 plant species examined, dramatic differences in the numbers of gene gains and losses were found in all lineages, suggesting the most members of the *FBX* superfamily were affected by rapid and multiple lineage-specific birth/death processes even between closely related species. The evolutionary patterns of *FBX* genes have striking similarities to the chemosensory receptor genes in vertebrates and insects, which also vary extensively in the numbers of functional genes and pseudogenes both within and between related species [4], [52], [53]. From these and other observations, Nei [54] proposed an evolution model called genomic drift to explain these seemly random changes in gene numbers. This genomic drift hypothesis postulates that, similar to genetic drift at the population level, neutral evolution generates at random widely different gene family sizes among species. While variations in sequence and copy number for drifting genes within a family may be largely inconsequential within populations thus enabling gene loss (death), some loci will become fixed if they help acclimate the individuals to the new niches and habitats [4].

Xu *et al.* [15] first speculated that genomic drift might play an important role in plant *FBX* gene evolution. Our more comprehensive analyses provide additional support. Included are the extraordinarily high and seemingly random birth/death rates of *FBX* genes among plant species, and the identification of numerous loci (STSP and *FBX* pseudogene groups) which are under more relaxed selection/evolution and appear to be transcriptionally inactive; all signatures of genomic drift [4], [54]. The recent sequencing of numerous interbreeding *A. thaliana* ecotypes suggests that such genomic drift of *FBX* loci can also be seen at the population level [13]. Along with the *NBS-LRR* gene family involved in pathogen defense, the *FBX* superfamily appears to be one of the most highly polymorphic gene families among the ecotypes. Under the assumption that the *FBX* superfamily (as possibly its *SKP1* partners) is affected by genomic drift, we speculate that a substantial pool of *FBX* loci are not functional but can act as seeds to generate new FBX protein functions. The more relaxed selection or neutral change of this diverse collection of seeds could randomly generate new SCF E3s with different expression patterns, altered binding affinities for existing targets, or with new target specificities. Although it is likely that most of these *FBX* loci eventually become pseudogenes, some could be selected for and retained to improve plant fitness to an ever changing environment through trial and error. One possible example of this success is the STSP pair ETP1/2 from *A. thaliana*. These FBX proteins participate in ethylene perception by helping remove the central regulator EIN2 [55]. While EIN2 is widely distributed among land plants, obvious ETP1/2 orthologs can only be found in *A. lyrata* among the 17 other plant species examined here, suggesting that ETP1/2 arose as lineage-specific modulators of ethylene signaling.

This pattern of genomic drift evolution has two important implications for the *FBX* gene superfamily. First, it suggests that that adaptive evolution of *FBX* genes occurs very frequently, more so than most gene families in plants. How the underpinning rapid birth/death mechanism(s) are selectively applied to *FBX* loci are unclear. Second, such an adaptive response may be relatively transient given the high birth and death rates in this family where most members do not persist over a long time. Nonetheless, many *FBX* loci have clearly survived over the course of plant evolution, especially members of the LTSP group. So, while genomic drift may have contributed to the initial emergence of new LTSP loci in general, strong selection pressures would promote their long-term retention. Contemporary examples of this selective retention could include the family of TIR1/ABF FBX proteins in *A. thaliana* and other plants which can detect natural and synthetic auxins and their AUX/IAA protein targets with different affinities [56], and the *A. thaliana* EBF1 and 2 FBX proteins whose differential expression patterns provide a mechanism to temporally regulate the abundance of the EIN3/EIL1 transcription factors during ethylene signaling [57]. If genomic drift is in action, its effect would dramatically reduce the final count of truly functional *FBX* genes in plants, but on the contrary identify an important reservoir exploited by the UPS to improve plant fitness.

## Materials and Methods

### Creation of the FBXD_Query and FBX_Ref Collections

The initial FBXD query collection of 6219 non-redundant FBXD peptide sequences was obtained from PFAM (http://PFAM.sanger.ac.uk/) and SMART (http://smart.embl-heidelberg.de/) (as of October, 2009) from both plant and non-plant sources and included the 694 potential *A. thaliana* FBXDs first described by Gagne *et al.* [12]. Redundant entries were removed by the CD-HIT program [58]. The initial FBXD query collection was used to screen each of the 18 plant proteomes for previously-annotated proteins bearing FBXD sequences by BLASTp (E-value ≤1) [27]. Additional plant and non-plant FBX protein sequences were retrieved from the protein RefSeqs of NCBI (http://www.ncbi.nlm.nih.gov/refseq), and the UniProtKB and UniRef databases (http://www.uniprot.org). The presence of a FBXD in each protein along with the identification of new FBXD sequences were determined by searching the PFAM database with HMMER3 (http://www.hmmer.org/) using an E-value ≤1 cutoff.

Newly predicted FBXD sequences, which were not part of the PFAM and SMART collections, were then used to search each of the 18 proteomes one more time. In total, 6575 and 0 new FBXD sequences and 7,570 and 64 plant *FBX* genes were found in the first and second searches, respectively (Figure 1A, Table S1). When combined with all FBXD/FBX protein sequence lists obtained above, we generated two non-redundant sequence collections, called FBXD_Query and FBX_Ref (File S1 and S2).

### Similarity-Based Re-annotation of *FBX* Genes

Via the re-annotation pipeline summarized in Figure 1, we searched each of the 18 unmasked plant genomes for all FBXD regions using the FBXD_Query database as the query. To predict the correct full-length coding sequence of each previously non-annotated *FBX* locus in each proteome database, a 10-kbp genomic sequence sandwiching the FBXD region was first retrieved and the adjacent sequences not predicted to be transcriptionally connected to the potential *FBX* gene coding sequence were removed by an iterative application of the Closing Target Trimming algorithm (CTT) (see below). Each trimmed genomic DNA sequence was then used as a query to search FBX_Ref to find homologous FBX protein sequences. The top two (if only two sequences similar to FBXD were found) or top three (if more than two sequences were found) scored (bit-score values) homologous protein sequences were used as references to back search the genomic DNA sequence to predict the transcript model and the correct coding sequence using GENEWISE. The coding sequence predicted with the highest GENEWISE score was taken as the best prediction of the *FBX* locus. To identify the FBXD region in previously or newly annotated FBX protein sequences, we used HMMER3 to search each protein sequence against PFAM database (E-value <1). Introns within the coding region were detected using BLAT [59] to align the genomic sequence and coding sequence of each *FBX* gene.

### Closing Target Trimming Algorithm (CTT)

To remove coding regions from adjacent genes in the 10-kbp window containing previously non-annotated *FBX* loci, we applied the CTT algorithm (Figure 1B). In brief, each ∼10-kb genomic DNA sequence was used to search FBX_Ref by BLASTx to identify similar protein sequences (E-value <1e-5) [27]. The top-scored (bit score value) protein sequence was used to search for coding regions with GENEWISE (score ≥50) in the ∼10-kb genomic sequence. If the coding region did not contain the target *FBXD* sequence, identified by the positions of the two regions in the ∼10-kb genomic, that part of genomic DNA sequence was trimmed. Sequential BLASTx/GENEWISE/trimming was iterated up to six times until a single potential *FBX* coding region with the best sequence match covered the target *FBXD* region first identified by tBLASTn. If a potential *FBX* coding region was not found after six rounds, the locus was omitted from further analysis.

### Inferring Birth/Death Events of *FBX* Genes

Results from an all-against-all BLASTp search (E-value <1e-5) of the full-length FBX protein sequences grouped the 10,811 FBX protein sequences into 351 clusters using MCL (granularity I = 4) [32]. The *FBX* genes in 47 of the clusters were from only one species; they were counted as gene gains in that species. For the remaining 304 clusters, multiple protein sequence alignments were generated with MUSCLE version 3.5 [60] (maximum iterations  = 16). Two large clusters (>1,000 members) were further divided into smaller subclusters based on neighbor-joining trees [61]. If the species number in a cluster/subcluster was less than 4 but larger than 1, the *FBX* genes in that cluster/subcluster were excluded for further calculation because the minimum number of species for phylogenetic reconstruction required ≥4 species. For the remaining clusters/subclusters, maximum likelihood trees were generated by RAxML (version 7.04) [62] using the PROTGAMMAJTT substitution model and default settings. Gene duplication and loss events were inferred by reconciling the gene tree for each cluster/subcluster with the species tree using Notung (version 2.5) [63]).

### *FBX* Pseudogene Predictions

The genomic DNA sequence of each plant *FBX* locus was retrieved from the corresponding genomic database and used to search the entire FBX protein reference collection (FBX_Ref) for potential homologs by BLASTx (E-value < 1e-5). The top two (if only two non-self hits were obtained) or top three (if more than two non-self hits were obtained) non-self hits were used as references to re-annotate the transcript model of the *FBX* locus by GENEWISE. If two reference sequences predicted the presence of either frameshifts or premature stop codons, the locus was classified as a pseudogene. Preliminary studies showed that the application of two references sequences helped reduce false classification of functional *FBX* genes as pseudogenes.

### *FBX* Gene Expression and Functional Predictions of *A. thaliana FBX* Genes

EST databases from each of the 18 plant species were retrieved from NCBI and used to search for representatives of the corresponding species *FBX* gene collection (last retrieved February 17, 2010). Relative expression was estimated by counting the total number of ESTs for each *FBX* locus. An EST was considered a true reflection of transcript expression if: (i) it had >95% identity to the query *FBX* coding region, (ii) at least 75% of the EST or the query sequence was aligned, and if (iii) at least 50 nucleotides of the EST was included in the alignment. Microarray expression data for individual *A. thaliana FBX* genes (as of January 12, 2010) were retrieved from the Nottingham Arabidopsis Stock Centre's microarray database (NASCArrays) (http://affymetrix.arabidopsis.info/narrays/experimentbrowse.pl), which is Minimum Information About a Microarray Experiment (MIAME) supportive [64]. The pairwise Pearson's correlation coefficient for each gene was calculated based on the ∼4000 microarray experiments and clustered and viewed using heatmap.2 in R. To support the proposed grouping of LTSP, STSP/pseudogene classifications and to predict the general functions of the LTSP and STSP genes, we compared the expression frequencies of each group as calculated by Fisher's exact test based on the number of expressed genes in the microarray relative to the average number of expressed genes. If a statistically significant enrichment of expressed *FBX* genes was observed in a microarray dataset, the biological functions tested by the microarray was retrieved. If two microarrays were used in the same experiment, only the one with the higher number of expressed *FBX* genes was analyzed.

### Calculation of *Ka/Ks* Values

To increase the strength of *Ka/Ks* calculations because many *FBX* genes appear to have been generated by the recent duplications, we used an alternative method to calculate the MRCAs. Here, the protein sequence of each *FBX* gene (Files S5) was used as a query to identify the top two non-self hits in the FBX_Ref dataset (File S2) by BLASTp and the corresponding transcript sequences were retrieved from File S8. The *Ka/Ks* value of the target gene was then calculated by first aligning its protein sequence with the two peptide sequences via T-coffee [65]. From the codon alignment and the guide neighbor-joining tree rooted at the midpoint using Phylip (http://evolution.genetics.washington.edu/phylip.html), the MRCA sequence of the three *FBX* genes was calculated using PAMP (PAML4 package [66]). The *Ka*, *Ks*, and *Ka/Ks* values of the target *FBX* gene was obtained by comparing its coding sequence to that of the MRCA using the pairwise model of CodeML (PAML4 package [66]). Sequences that were either too similar (*Ks*<0.005) or too divergent (*Ks*>3) were excluded. In total, 10,596 of 10,811 (98%) total *FBX* genes generated with this three-way comparison had useable *Ks* values between these extremes (File S9). By the same method, the *Ka*, *Ks*, and *Ka/Ks* values of *FBXD*, and Δ*FBXD* regions were generated.

To test whether an *FBX* gene is under neutral change, we collected the maximum likelihood values ML1 and ML2 from two runs of CodeML with the *Ka/Ks* ratio of the full-length zcoding sequence fixed at 1 and free, respectively. From these values, we calculated the likelihood ratio (LR)  = 2(lnML1-lnML2) as described by Nekrutenko *et al.* [35] (File S9). For LR values less than 3.84 (X_0.05_^2^), the *Ka/Ks* value for the *FBX* gene should approach 1 and thus be indicative of neutral change.

### Detection of Tandemly or Segmentally Duplicated *FBX* Genes

For each of 18 plant species, the entire lists of tandem or segmental duplicated genes (including the new annotated *FBX* genes obtained in this work) were predicted by DAGchainer [37] with the default settings based on sequence similarities and the genomic position information of annotated genes. *FBX* genes affected by these duplication mechanisms were then extracted from this list.

## Supporting Information

Table S1 **The database resources of 18 plant genome annotations used in this work.**
(DOC)

Table S2 **Summary of different** ***FBX*** **gene annotation steps in the 18 plant species.**
(DOC)

Table S3 **Functionally characterized** ***FBX*** **genes from** ***A. thaliana*** **.**
(DOC)

Table S4 **Biochemical evidence in the literature confirming the interaction between** ***A. thaliana*** **FBX proteins and one or more SKP1/ASK proteins.**
(DOC)

Table S5 **The enrichment of various domain (dmn) combinations in the putative substrate recruitment module of FBX proteins.** *p* indicates the enrichment probability of FBX proteins with a specific domain/domain combination as compared to that for the average number of FBX proteins. In total, 3,269 domains/domain combinations (including no prediction group) were identified from 10,811 FBX proteins.
(DOC)

Table S6 **The enrichment comparison of domain (dmn) combinations in the putative substrate recruitment module of the translated protein products from** ***FBX*** **pseudogenes and protein-coding genes.** *p* (left) indicates the domain enrichment probability in the translated protein products from *FBX* pseudogenes. *p* (right) indicates the domain enrichment probability in the translated protein products from protein-coding genes.
(DOC)

Table S7 **Fisher's exact test of the enrichment or depletion of domain (dmn) combinations in the putative substrate recruitment module of FBX proteins in each plant species.** *p* (left) indicates the domain enrichment probability in a species. *p* (right) indicates the domain depletion probability in a species.
(DOC)

Table S8 **Wilcoxon rank sum test (one tailed) of** ***Ka/Ks*** **values of the LTS protein-coding** ***FBX*** **genes (LTSP), STS protein-coding** ***FBX*** **genes (STSP) and** ***FBX*** **pseudogenes (ψ in each plant species.**
(DOC)

Table S9 **Wilcoxon rank sum test (one tailed) of** ***Ka/Ks*** **values from** ***FBXD*** **and Δ** ***FBXD*** **identified within the LTS protein-coding** ***FBX*** **gene (LTSP), STS protein-coding** ***FBX*** **gene (STSP), and** ***FBX*** **pseudogene (ψ groups in each of the 18 plant species.**
(DOC)

Table S10 **Enrichment in various microarray datasets and functional prediction of expressed** ***FBX*** **genes from LTS and STS groups of** ***A. thaliana*** **.**
(DOC)

Figure S1 **Size distributions in nucleotides of the complete set of** ***FBX*** **genes predicted from all 18 plant species (see Table S1 for list and abbreviation for each species).**
(TIF)

Figure S2 **Evaluations of the CTT and the similarity-based** ***FBX*** **gene annotation protocols.** *A. thaliana* (*At*). *O. sativa* (*Os*). “Frame shift” indicates *FBX* genes containing one or more reading-frame shifts. “Premature Stop” indicates *FBX* genes containing premature stop codon(s). “Protein” indicates *FBX* protein-coding genes.
(TIF)

Figure S3 **The distributions of** ***FBXD Ka/Ks*** **values of** ***FBX*** **genes from the LTSP, STSP and** ***FBX*** **pseudogene groups in each of 18 plant species.** The *Ka/Ks* value for each *FBXD* sequence was calculated by comparing *FBXD* sequence to the MRCA transcript sequence using the method of Goldman and Yang [34].
(TIF)

Figure S4 **The distributions of Δ** ***FBXD Ka/Ks*** **values of** ***FBX*** **genes from the LTSP, STSP and** ***FBX*** **pseudogene groups in each of 18 plant species.** The *Ka/Ks* value for each Δ*FBXD* sequence was calculated by comparing Δ*FBXD* sequence to the MRCA transcript sequence using the method of Goldman and Yang [34].
(TIF)

Figure S5 **The distributions of LTSP, STSP and** ***FBX*** **pseudogene loci along the chromosomes of** ***A. thaliana*** **.**
(TIF)

Figure S6 **Microarray data correlations of** ***A. thaliana FBX*** **genes from cluster b in** **Figure 8A** **, and 28 internal non-plant genes from NASCArrays.** The Pearson's correlation coefficients were calculated as described in Figure 8. The dendrogram on the top of the panel showed the hierarchical clustering based on the dissimilarities of Pearson's correlation coefficients. The bar codes indicate the distributions of LTS *FBX* genes (red color), STS *FBX* genes (blue color), and the 28 non-plant internal control genes (green color).
(TIF)

File S1 **The non-redundant peptide sequences from the 13,565 FBXDs in the FBXD_Query collection.** The FBXD peptide sequences were collected from three resources: (i) PFAM and SMART, (ii) FBXD peptide sequences of FBX proteins predicted from the 18 plant proteomes, and (iii) FBXD peptide sequences of FBX proteins from the species outside the 18 plant proteomes which were collected from UniProtKB, UniRef, and/or the NCBI reference sequence database RefSeq.
(TXT)

File S2 **The non-redundant protein sequences from the 13,325 reference** ***FBX*** **genes in the FBX_Ref collection.** The FBX protein sequences were collected from two resources: (i) FBX protein sequences predicted from the 18 plant species, and (ii) FBX protein sequences from the species outside the 18 plant species, which were collected from UniProtKB, UniRef, and/or the NCBI reference sequence databases RefSeq.
(TXT)

File S3 **The comparison between the re-annotated transcript sequence and the original annotated transcript sequence of each** ***FBX*** **gene from** ***A. thaliana*** **using bl2seq.** The re-annotated transcript sequences were obtained using CTT and similarity-based annotation pipeline as described in the Materials and Methods.
(TXT)

File S4 **The comparison between the re-annotated transcript sequence and the original annotated transcript sequence of each** ***FBX*** **gene from** ***O. sativa*** **using bl2seq.** The re-annotated transcript sequences were obtained using CTT and the similarity-based annotation pipeline as described in the Materials and Methods.
(TXT)

File S5 **The protein sequences derived from all 10,811** ***FBX*** **genes obtained from the 18 plant species.** The header for each FBX protein sequence contains two items. The first is a new ID used in this work and the second is a previous ID used in the original annotation of each species. “New_annotated” indicates the *FBX* genes newly annotated in this work.
(TXT)

File S6 **The coding sequences of all 10,811** ***FBX*** **genes obtained from the 18 plant species.**
(TXT)

File S7 **The list of tandem and segmental duplicated** ***FBX*** **genes and pseudogenes in each of the 18 plant species.**
(TXT)

File S8 **The coding sequences of 13,325 reference** ***FBX*** **genes.** The coding sequence for each *FBX* gene was retrieved from NCBI mRNA database, EMBL coding sequence database, and/or the annotation pipeline from the 18 plant species described here.
(TXT)

File S9 ***Ka/Ks*** **and** ***Ks*** **values of** ***FBX*** **genes from the 18 plant species.** The *Ka/Ks* value for each full-length sequence was calculated by comparing it to the MRCA transcript sequence using the method of Goldman and Yang [34].
(TXT)
