# Supplementary material for: Phylogenetic Comparison of F-Box (FBX) Gene Superfamily within the Plant Kingdom Reveals Divergent Evolutionary Histories Indicative of Genomic Drift
Source: PLoS One. 2011 Jan 28;6(1):e16219. doi: 10.1371/journal.pone.0016219 (PMC3030570; doi:10.1371/journal.pone.0016219)
Supplement: Table S1 — The database resources of 18 plant genome annotations used in this work. (DOC) [file pone.0016219.s001.doc]

**Table S1.** The database resources of 18 plant genome annotations used in this work.

| **Organism** | **Common name** | **Life Habit*** | **Version** | **Source** | **Release year** |
| --- | --- | --- | --- | --- | --- |
| *Arabidopsis lyrata* | Lyre-leaved rock cress | HP | JGI release 1.0 | http://genome.jgi-psf.org/Araly1/Araly1.download.ftp.html | 2008 |
| *Arabidopsis thaliana* | Mouse-ear cress | HA | TAIR9 | ftp://ftp.arabidopsis.org/home/tair/Sequences/ | 2009 |
| *Brachypodium distachyon* | Purple false brome | HA | JGI v1.0 8x assembly of strain Bd21 |  | 2009 |
| *Carica papaya* | Papaya | WP | ASGPB release of 2008 | ftp://asgpb.mhpcc.hawaii.edu/papaya/ | 2008 |
| Chlamydomonas reinhardtii | Green algae | - | Augustus u9 annotation of JGI v4 | http://www.phytozome.net/ | 2010 |
| Cucumis sativus | Cucumber | HA | Roche 454-XLR assembly and JGI v1 | http://www.phytozome.net/ | 2010 |
| *Glycine max* | Soybean | HA | JGI Glyma1.0 | http://www.phytozome.net/ | 2009 |
| *Manihot esculenta* | Cassava | WP | JGI/Roche v1.1 assembly and annotation | http://www.phytozome.net/ | 2009 |
| *Medicago truncatula* | Barrel medic | HA | Medicago Genome Sequence Consortium Mt3.0 | http://www.phytozome.net/ | 2009 |
| *Mimulus guttatus* | Monkey flower | HP | JGI 7x assembly of strain IM62, annotation v1.0 | http://www.phytozome.net/ | 2010 |
| *Oryza sativa* | Rice | HA | MSU Release 6.1 | ftp://ftp.plantbiology.msu.edu/pub/data/Eukaryotic_Projects/o_sativa/annotation_dbs/ | 2009 |
| *Physcomitrella patens* | Moss | - | JGI v1.1 assembly and annotation | http://www.phytozome.net/ | 2009 |
| *Populus trichocarpa* | Poplar | WP | JGI v2.0 | http://www.phytozome.net/ | 2010 |
| *Ricinus communis* | Castor bean | HA | TIGR/JCVI release v0.1 | http://castorbean.jcvi.org/castorbean_downloads.shtml | 2008 |
| *Selaginella moellendorffii* | Spikemoss | - | JGI v1.0 assembly and annotation | http://genome.jgi-psf.org/Selmo1/Selmo1.download.ftp.html | 2007 |
| *Sorghum bicolor* | Sweet Sorghum | HA | MIPS/PASA Version 1.0 | http://www.phytozome.net/ | 2008 |
| *Vitis vinifera* | Grape | WP | International Grape Genome Program Sept 2007 release | http://www.vitaceae.org/index.php/Genome_Sequencing | 2007 |
| *Zea mays* | Maize/Corn | HA | Zmb73 release 4a.53 | http://ftp.maizesequence.org/current/ | 2009 |

* HA, herbaceous annual plant; HP, herbaceous perennial plant; WP, woody perennial plant.
