# Supplementary material for: Phylogenetic Comparison of F-Box (FBX) Gene Superfamily within the Plant Kingdom Reveals Divergent Evolutionary Histories Indicative of Genomic Drift
Source: PLoS One. 2011 Jan 28;6(1):e16219. doi: 10.1371/journal.pone.0016219 (PMC3030570; doi:10.1371/journal.pone.0016219)
Supplement: Table S2 — Summary of different FBX gene annotation steps in the 18 plant species. (DOC) [file pone.0016219.s002.doc]

**Table S2.** Summary of different *FBX* gene annotation steps in the 18 plant species.

| **Species** | **Blastp-HMMER prediction** | | | **tBlastn-CTT-GENEWISE-HMMER prediction** | | | | | | **Final predictions** |
| --- | --- | --- | --- | --- | --- | --- | --- | --- | --- | --- |
|  | First Blastp | SecondBlastp | Sum | New hits | Closing target trimming | 2 homolog annotation | Unique loci | HMMER_new *FBX*_genes | Sum |  |
| *Al* | 861 | 1 | 862 (63.9) | 947 | 715 | 643 | 583 | 488 | 488 (36.1) | 1350 |
| *At* | 707 | 3 | 710 (79.2) | 570 | 305 | 273 | 254 | 187 | 187 (20.8) | 897 |
| *Bd* | 493 | 4 | 497 (49.8) | 1148 | 754 | 687 | 651 | 501 | 501 (50.2) | 998 |
| *Cp* | 151 | 0 | 151 (76.3) | 231 | 131 | 119 | 109 | 47 | 47 (23.7) | 198 |
| *Cr* | 73 | 1 | 74 (84.1) | 537 | 131 | 110 | 110 | 14 | 14 (15.9) | 88 |
| *Cs* | 167 | 1 | 168 (81.2) | 196 | 68 | 60 | 58 | 39 | 39 (18.8) | 207 |
| *Gm* | 455 | 2 | 457 (65.1) | 639 | 430 | 393 | 366 | 245 | 245 (34.9) | 702 |
| *Me* | 292 | 4 | 296 (91.6) | 163 | 79 | 62 | 55 | 27 | 27 ( 8.4) | 323 |
| *Mg* | 444 | 0 | 444 (49.2) | 843 | 618 | 552 | 518 | 459 | 459 (50.8) | 903 |
| *Mt* | 948 | 29 | 977 (85.1) | 559 | 383 | 338 | 308 | 171 | 171 (14.9) | 1148 |
| *Os* | 797 | 2 | 799 (82.3) | 586 | 364 | 331 | 314 | 172 | 172 (17.7) | 971 |
| *Pp* | 229 | 3 | 232 (89.9) | 204 | 49 | 35 | 35 | 26 | 26 (10.1) | 258 |
| *Pt* | 373 | 0 | 373 (87.8) | 367 | 183 | 161 | 143 | 52 | 52 (12.2) | 425 |
| *Rc* | 219 | 1 | 220 (88.0) | 194 | 81 | 67 | 58 | 30 | 30 (12.0) | 250 |
| *Sb* | 664 | 3 | 667 (81.6) | 515 | 313 | 268 | 249 | 150 | 150 (18.4) | 817 |
| *Sm* | 233 | 6 | 239 (43.9) | 990 | 542 | 428 | 404 | 305 | 305 (56.1) | 544 |
| *Vv* | 178 | 2 | 180 (57.1) | 365 | 213 | 197 | 191 | 135 | 135 (42.9) | 315 |
| *Zm* | 286 | 2 | 288 (69.1) | 376 | 269 | 249 | 242 | 129 | 129 (30.9) | 417 |
| Total | 7570 | 64 | 7634 (70.6) | 9430 | 5628 | 4973 | 4648 | 3177 | 3177 (29.4) | 10811 |

*The percent of total *FBX* genes in each group is listed in the parenthesis.
