# Supplementary material for: Phylogenetic Comparison of F-Box (FBX) Gene Superfamily within the Plant Kingdom Reveals Divergent Evolutionary Histories Indicative of Genomic Drift
Source: PLoS One. 2011 Jan 28;6(1):e16219. doi: 10.1371/journal.pone.0016219 (PMC3030570; doi:10.1371/journal.pone.0016219)
Supplement: Table S3 — Functionally characterized FBX genes from A. thaliana . (DOC) [file pone.0016219.s003.doc]

**Table S3.** Functionally characterized *FBX* genes from *A. thaliana*.

| **Number** | **New_id** | **AGI_ID** | **Gene_name** | **E-value** | **OG_taxono-mic_scale** | **Briefly_functional_description** | **References** |
| --- | --- | --- | --- | --- | --- | --- | --- |
| 1 | At_F0001 | AT1G21760 | FBP7 | 6.6E-14 | 18 | Required for protein synthesis during temperature stress | [1] |
| 2 | At_F0416 | AT2G25490 | EBF1 | 4.7E-07 | 18 | Controlling the stability of EIN3 and EIL1 in ethylene signaling pathway | [2,3,4,5] |
| 3 | At_F0442 | AT5G25350 | EBF2 | 8.0E-07 | 18 | Controlling the stability of EIN3 and EIL1 in ethylene signaling pathway | [2,3,4,5] |
| 4 | At_F0418 | AT5G21040 | FBX2 | 4.8E-07 | 18 | Negatively regulating phosphate starvation response | [6] |
| 5 | At_F0318 | AT4G33210 | SLOMO | 3.0E-08 | 18 | Regulating auxin homeostasis in the shoot meristem | [7] |
| 6 | At_F0302 | AT4G07400 | VFB3 | 2.1E-08 | 17 | Controlling plant growth and development (lateral root formation). | [8] |
| 7 | At_F0443 | AT1G47056 | VFB1 | 8.2E-07 | 17 | Controlling plant growth and development (lateral root formation). | [8] |
| 8 | At_F0570 | AT4G03190 | AFB1 | 6.1E-05 | 17 | Auxin receptor, controlling the stability of AUX/IAA proteins | [9,10] |
| 9 | At_F0586 | AT3G62980 | TIR1 | 1.4E-04 | 17 | Auxin receptor, controlling the stability of AUX/IAA proteins | [9,10,11,12] |
| 10 | At_F0626 | AT3G26810 | AFB2 | 8.1E-04 | 17 | Auxin receptor, controlling the stability of AUX/IAA proteins | [9,10] |
| 11 | At_F0628 | AT1G12820 | AFB3 | 9.6E-04 | 17 | Auxin receptor, controlling the stability of AUX/IAA proteins | [9,10] |
| 12 | At_F0016 | AT3G61590 | HAWIIAN SKIRT | 9.3E-12 | 16 | Regulating organ fusion. The loss of function caused the fusion of sepals | [13] |
| 13 | At_F0267 | AT1G30950 | UFO | 1.2E-08 | 16 | Controlling floral development | [14,15,16,17] |
| 14 | At_F0338 | AT3G60350 | ARABIDILLO2 | 6.1E-08 | 16 | Controlling lateral root branching | [18] |
| 15 | At_F0451 | AT2G44900 | ARABIDILLO1 | 1.0E-06 | 16 | Controlling lateral root branching | [18] |
| 16 | At_F0485 | AT1G76900 | ATTLP1 | 3.0E-06 | 16 | The same subfamily of ATTLP9, which is potentially related to ABA signaling | [19] |
| 17 | At_F0548 | AT1G25280 | ATTLP10 | 2.8E-05 | 16 | The same subfamily of ATTLP9, which is potentially related to ABA signaling | [19] |
| 18 | At_F0589 | AT2G47900 | ATTLP3 | 1.7E-04 | 16 | The same subfamily of ATTLP9, which is potentially related to ABA signaling | [19] |
| 19 | At_F0234 | AT4G24210 | SLY1 | 6.0E-09 | 15 | GA signal perception through targeting the degradation of DELLA proteins | [20,21,22] |
| 20 | At_F0297 | AT5G48170 | SNE/SLY2 | 1.8E-08 | 15 | GA signal perception through targeting the degradation of DELLA proteins | [23] |
| 21 | At_F0474 | AT5G57360 | ZEITLUPE/ZTL | 1.8E-06 | 15 | Light receptor, controlling the stability of TOC1 and PR5 | [24,25] |
| 22 | At_F0542 | AT2G18915 | LKP2 | 2.0E-05 | 15 | Light receptor, mildly controlling the stability of CDF1, TOC1 and PR5 | [24,26] |
| 23 | At_F0585 | AT1G68050 | FKF1 | 1.4E-04 | 15 | Light receptor, controlling the stability of CDF1, TOC1 and PR5 | [24,27] |
| 24 | At_F0508 | AT1G21410 | SKP2A | 7.5E-06 | 15 | Positively regulating cell division through degrading the E2FC/DPB transcription repressor | [28,29] |
| 25 | At_F0516 | AT1G77000 | SKP2B | 8.7E-06 | 15 | Regulating cell cycle | [30] |
| 26 | At_F0629 | AT1G47270 | ATTLP6 | 1.1E-03 | 15 | The same subfamily of ATTLP9, which is potentially related to ABA signaling | [19] |
| 27 | At_F0684 | AT2G18280 | ATTLP2 | 8.5E-02 | 15 | The same subfamily of ATTLP9, which is potentially related to ABA signaling | [19] |

**Table S3** (continued)

| **Number** | **New_id** | **AGI_ID** | **Gene_name** | **E-value** | **OG_taxono-mic_scale** | **Briefly_functional_description** | **References** |
| --- | --- | --- | --- | --- | --- | --- | --- |
| 28 | At_F0676 | AT2G42620 | MAX2/ORE9 | 4.6E-02 | 15 | Related to shoot branching, a putative strigolectone receptor | [31,32] |
| 29 | At_F0563 | AT3G54650 | FBL17 | 4.8E-05 | 14 | Controlling germline proliferation through targeting the degradation of cyclin-dependent kinase inhibitors KRP6/7 | [33,34] |
| 30 | At_F0569 | AT4G02440 | EID1 | 5.9E-05 | 14 | Negative regulator in phytochrome A-specific light signaling | [35,36] |
| 31 | At_F0653 | AT1G53320 | ATTLP7 | 4.2E-03 | 14 | The same subfamily of ATTLP9, which is potentially related to ABA signaling | [19] |
| 32 | At_F0662 | AT5G49980 | AFB5 | 1.4E-02 | 14 | Auxin receptor, controlling the stability of AUX/IAA proteins | [10,37] |
| 33 | At_F0669 | AT4G24390 | AFB4 | 2.5E-02 | 14 | Auxin receptor, controlling the stability of AUX/IAA proteins | [10] |
| 34 | At_F0705 | AT2G39940 | COI1 | 7.3E-01 | 14 | JA-Ile receptor, controlling the stability of JAZ proteins | [38,39,40] |
| 35 | At_F0402 | AT2G24540 | AFR | 3.1E-07 | 13 | Attenuated far-red light response regulator, potentially controlling the turnover of a repressor of phyA signaling | [41] |
| 36 | At_F0397 | AT4G12560 | CPR30 | 2.8E-07 | 10 | Negative regulator of the defense response in *Arabidopsis* | [42] |
| 37 | At_F0225 | AT3G50080 | VFB2 | 4.6E-09 | 7 | Controlling plant growth and development (lateral root formation) | [8] |
| 38 | At_F0304 | AT5G67250 | VFB4 | 2.1E-08 | 7 | Controlling plant growth and development (lateral root formation) | [8] |
| 39 | At_F0640 | AT1G43640 | ATTLP5 | 1.6E-03 | 5 | The same subfamily of ATTLP9, which is potentially related to ABA signaling | [19] |
| 40 | At_F0125 | AT3G18910 | ETP2 | 6.3E-10 | 2 | Controlling the stability of EIN2 in ethylene signaling pathway | [43] |
| 41 | At_F0127 | AT2G17310 | SON1 | 6.7E-10 | 2 | Related to pathogen defense | [44] |
| 42 | At_F0158 | AT3G18980 | ETP1 | 1.2E-09 | 2 | Controlling the stability of EIN2 in ethylene signaling pathway | [43] |
| 43 | At_F0421 | AT3G06380 | ATTLP9 | 5.0E-07 | 2 | Potentially related to ABA signaling | [19] |
| 44 | At_F0620 | AT5G18680 | ATTLP11 | 4.5E-04 | 2 | The same subfamily of ATTLP9, which is potentially related to ABA signaling | [19] |
| 45 | At_F0247 | AT2G31470 | DOR | 8.2E-09 | 2 | Negative regulator of drought resistance | [45] |
| 46 | At_F0459 | AT3G22650 | CEG | 1.2E-06 | 0 | Negatively regulating lateral root development | [46] |
| 47 | At_F0670 | AT1G61940 | ATTLP4 | 2.7E-02 | 0 | The same subfamily of ATTLP9, which is potentially related to ABA signaling | [19] |

**References for Table S3:**

1. Calderon-Villalobos LI, Nill C, Marrocco K, Kretsch T, Schwechheimer C (2007) The evolutionarily conserved *Arabidopsis thaliana* F-Box protein AtFBP7 is required for efficient translation during temperature stress. Gene 392: 106-116.

2. Gagne JM, Smalle J, Gingerich DJ, Walker JM, Yoo SD, et al. (2004) *Arabidopsis* EIN3-binding F-Box 1 and 2 form ubiquitin-protein ligases that repress ethylene action and promote growth by directing EIN3 degradation. Proc Natl Acad Sci USA 101: 6803-6808.

3. Guo H, Ecker JR (2003) Plant responses to ethylene gas are mediated by SCF^EBF1/EBF2^-dependent proteolysis of EIN3 transcription factor. Cell 115: 667-677.

4. Potuschak T, Lechner E, Parmentier Y, Yanagisawa S, Grava S, et al. (2003) EIN3-dependent regulation of plant ethylene hormone signaling by two *Arabidopsis* F-Box proteins: EBF1 and EBF2. Cell 115: 679-689.

5. Binder BM, Walker JM, Gagne JM, Emborg TJ, Hemmann G, et al. (2007) The *Arabidopsis* EIN3 binding F-Box proteins EBF1 and EBF2 have distinct but overlapping roles in ethylene signaling. Plant Cell 19: 509-523.

6. Chen ZH, Jenkins GI, Nimmo HG (2008) Identification of an F-Box protein that negatively regulates P(i) starvation responses. Plant Cell Physiol 49: 1902-1906.

7. Lohmann D, Stacey N, Breuninger H, Jikumaru Y, Muller D, et al. (2010) SLOW MOTION is required for within-plant auxin homeostasis and normal timing of lateral organ initiation at the shoot meristem in *Arabidopsis*. Plant Cell 22: 335-348.

8. Schwager KM, Calderon-Villalobos LI, Dohmann EM, Willige BC, Knierer S, et al. (2007) Characterization of the *VIER F-BOX PROTEINE* genes from *Arabidopsis* reveals their importance for plant growth and development. Plant Cell 19: 1163-1178.

9. Dharmasiri N, Dharmasiri S, Weijers D, Lechner E, Yamada M, et al. (2005) Plant development is regulated by a family of auxin receptor F-Box proteins. Dev Cell 9: 109-119.

10. Parry G, Calderon-Villalobos LI, Prigge M, Peret B, Dharmasiri S, et al. (2009) Complex regulation of the TIR1/AFB family of auxin receptors. Proc Natl Acad Sci USA 106: 22540-22545.

11. Dharmasiri N, Dharmasiri S, Estelle M (2005) The F-Box protein TIR1 is an auxin receptor. Nature 435: 441-445.

12. Tan X, Calderon-Villalobos LI, Sharon M, Zheng C, Robinson CV, et al. (2007) Mechanism of auxin perception by the TIR1 ubiquitin ligase. Nature 446: 640-645.

13. Gonzalez-Carranza ZH, Rompa U, Peters JL, Bhatt AM, Wagstaff C, et al. (2007) *HAWAIIAN SKIRT*: an F-Box gene that regulates organ fusion and growth in *Arabidopsis*. Plant Physiol 144: 1370-1382.

14. Durfee T, Roe JL, Sessions RA, Inouye C, Serikawa K, et al. (2003) The F-Box-containing protein UFO and AGAMOUS participate in antagonistic pathways governing early petal development in *Arabidopsis*. Proc Natl Acad Sci USA 100: 8571-8576.

15. Levin JZ, Meyerowitz EM (1995) *UFO*: an *Arabidopsis* gene involved in both floral meristem and floral organ development. Plant Cell 7: 529-548.

16. Samach A, Klenz JE, Kohalmi SE, Risseeuw E, Haughn GW, et al. (1999) The *UNUSUAL FLORAL ORGANS* gene of *Arabidopsis thaliana* is an F-Box protein required for normal patterning and growth in the floral meristem. Plant J 20: 433-445.

17. Zhao D, Yang M, Solava J, Ma H (1999) The *ASK1* gene regulates development and interacts with the *UFO* gene to control floral organ identity in *Arabidopsis*. Dev Genet 25: 209-223.

18. Coates JC, Laplaze L, Haseloff J (2006) Armadillo-related proteins promote lateral root development in *Arabidopsis*. Proc Natl Acad Sci USA 103: 1621-1626.

19. Lai CP, Lee CL, Chen PH, Wu SH, Yang CC, et al. (2004) Molecular analyses of the *Arabidopsis* TUBBY-like protein gene family. Plant Physiol 134: 1586-1597.

20. Dill A, Thomas SG, Hu J, Steber CM, Sun TP (2004) The *Arabidopsis* F-Box protein SLEEPY1 targets gibberellin signaling repressors for gibberellin-induced degradation. Plant Cell 16: 1392-1405.

21. Fu X, Richards DE, Fleck B, Xie D, Burton N, et al. (2004) The *Arabidopsis* mutant sleepy1^gar2-1^ protein promotes plant growth by increasing the affinity of the SCF^SLY1^ E3 ubiquitin ligase for DELLA protein substrates. Plant Cell 16: 1406-1418.

22. McGinnis KM, Thomas SG, Soule JD, Strader LC, Zale JM, et al. (2003) The *Arabidopsis* *SLEEPY1* gene encodes a putative F-Box subunit of an SCF E3 ubiquitin ligase. Plant Cell 15: 1120-1130.

23. Strader LC, Ritchie S, Soule JD, McGinnis KM, Steber CM (2004) Recessive-interfering mutations in the gibberellin signaling gene *SLEEPY1* are rescued by overexpression of its homologue, *SNEEZY*. Proc Natl Acad Sci USA 101: 12771-12776.

24. Baudry A, Ito S, Song YH, Strait AA, Kiba T, et al. (2010) F-Box proteins FKF1 and LKP2 act in concert with ZEITLUPE to control *Arabidopsis* clock progression. Plant cell 22: 606-622.

25. Somers DE, Schultz TF, Milnamow M, Kay SA (2000) *ZEITLUPE* encodes a novel clock-associated PAS protein from *Arabidopsis*. Cell 101: 319-329.

26. Schultz TF, Kiyosue T, Yanovsky M, Wada M, Kay SA (2001) A role for LKP2 in the circadian clock of *Arabidopsis*. Plant Cell 13: 2659-2670.

27. Nelson DC, Lasswell J, Rogg LE, Cohen MA, Bartel B (2000) FKF1, a clock-controlled gene that regulates the transition to flowering in *Arabidopsis*. Cell 101: 331-340.

28. del Pozo JC, Diaz-Trivino S, Cisneros N, Gutierrez C (2006) The balance between cell division and endoreplication depends on E2FC-DPB, transcription factors regulated by the ubiquitin-SCF^SKP2A^ pathway in *Arabidopsis*. Plant Cell 18: 2224-2235.

29. Jurado S, Diaz-Trivino S, Abraham Z, Manzano C, Gutierrez C, et al. (2008) SKP2A, an F-Box protein that regulates cell division, is degraded via the ubiquitin pathway. Plant J 53: 828-841.

30. Ren H, Santner A, del Pozo JC, Murray JA, Estelle M (2008) Degradation of the cyclin-dependent kinase inhibitor KRP1 is regulated by two different ubiquitin E3 ligases. Plant J 53: 705-716.

31. Stirnberg P, Furner IJ, Ottoline Leyser HM (2007) MAX2 participates in an SCF complex which acts locally at the node to suppress shoot branching. Plant J 50: 80-94.

32. Woo HR, Chung KM, Park JH, Oh SA, Ahn T, et al. (2001) ORE9, an F-Box protein that regulates leaf senescence in *Arabidopsis*. Plant Cell 13: 1779-1790.

33. Gusti A, Baumberger N, Nowack M, Pusch S, Eisler H, et al. (2009) The *Arabidopsis thaliana* F-Box protein FBL17 is essential for progression through the second mitosis during pollen development. PLoS ONE 4: e4780.

34. Kim HJ, Oh SA, Brownfield L, Hong SH, Ryu H, et al. (2008) Control of plant germline proliferation by SCF^FBL17^ degradation of cell cycle inhibitors. Nature 455: 1134-1137.

35. Dieterle M, Zhou YC, Schafer E, Funk M, Kretsch T (2001) EID1, an F-Box protein involved in phytochrome A-specific light signaling. Genes Dev 15: 939-944.

36. Marrocco K, Zhou Y, Bury E, Dieterle M, Funk M, et al. (2006) Functional analysis of EID1, an F-Box protein involved in phytochrome A-dependent light signal transduction. Plant J 45: 423-438.

37. Walsh TA, Neal R, Merlo AO, Honma M, Hicks GR, et al. (2006) Mutations in an auxin receptor homolog AFB5 and in SGT1b confer resistance to synthetic picolinate auxins and not to 2,4-dichlorophenoxyacetic acid or indole-3-acetic acid in *Arabidopsis*. Plant Physiol 142: 542-552.

38. Chini A, Fonseca S, Fernandez G, Adie B, Chico JM, et al. (2007) The JAZ family of repressors is the missing link in jasmonate signalling. Nature 448: 666-671.

39. Sheard LB, Tan X, Mao H, Withers J, Ben-Nissan G, et al. (2010) Jasmonate perception by inositol-phosphate-potentiated COI1-JAZ co-receptor. Nature 468: 400-405.

40. Thines B, Katsir L, Melotto M, Niu Y, Mandaokar A, et al. (2007) JAZ repressor proteins are targets of the SCF^COI1^ complex during jasmonate signalling. Nature 448: 661-665.

41. Harmon FG, Kay SA (2003) The F-Box protein AFR is a positive regulator of phytochrome A-mediated light signaling. Curr Biol 13: 2091-2096.

42. Gou M, Su N, Zheng J, Huai J, Wu G, et al. (2009) An F-Box gene, *CPR30*, functions as a negative regulator of the defense response in *Arabidopsis*. Plant J 60: 757-770.

43. Qiao H, Chang KN, Yazaki J, Ecker JR (2009) Interplay between ethylene, ETP1/ETP2 F-Box proteins, and degradation of EIN2 triggers ethylene responses in *Arabidopsis*. Genes Dev 23: 512-521.

44. Kim HS, Delaney TP (2002) *Arabidopsis* SON1 is an F-Box protein that regulates a novel induced defense response independent of both salicylic acid and systemic acquired resistance. Plant Cell 14: 1469-1482.

45. Zhang Y, Xu W, Li Z, Deng XW, Wu W, et al. (2008) F-Box protein DOR functions as a novel inhibitory factor for abscisic acid-induced stomatal closure under drought stress in *Arabidopsis*. Plant Physiol 148: 2121-2133.

46. Dong L, Wang L, Zhang Y, Deng X, Xue Y (2006) An auxin-inducible F-Box protein CEGENDUO negatively regulates auxin-mediated lateral root formation in *Arabidopsis*. Plant Mol Biol 60: 599-615.
