# Supplementary material for: Phylogenetic Comparison of F-Box (FBX) Gene Superfamily within the Plant Kingdom Reveals Divergent Evolutionary Histories Indicative of Genomic Drift
Source: PLoS One. 2011 Jan 28;6(1):e16219. doi: 10.1371/journal.pone.0016219 (PMC3030570; doi:10.1371/journal.pone.0016219)
Supplement: Table S4 — Biochemical evidence in the literature confirming the interaction between A. thaliana FBX proteins and one or more SKP1/ASK proteins. (DOC) [file pone.0016219.s004.doc]

***Table S4.*** *Biochemical evidence in the literature confirming the interaction between A. thaliana FBX proteins and one or more SKP1/ASK proteins.*

| - **Number** | - **New_ID** | - **AGI_ID Published** | - **ASKs Interacted** | - **Method^a^** | - **Function Known** | - **OG Taxa** | - **E-value** | - **References** |
| --- | --- | --- | --- | --- | --- | --- | --- | --- |
| - 1 | - At_F0225 | - AT3G50080 | - ASK2 | - Y2H | - VFB2 | - 7 | 4.6E-09 | - [1] |
| - 2 | - At_F0304 | - AT5G67250 | - ASK1_2_more | - Y2H | - VFB4 | - 7 | 2.1E-08 | - [2] |
| - 3 | - At_F0583 | - AT1G78100 | - ASK1_2_more | - Y2H |  | - 8 | 1.2E-04 | - [2] |
| - 4 | - At_F0269 | - AT1G80110 | - ASK1_2 | - Y2H |  | - 9 | 1.2E-08 | - [3] |
| - 5 | - At_F0276 | - AT2G02360 | - ASK_2_MORE | - Y2H |  | - 9 | 1.4E-08 | - [4] |
| - 6 | - At_F0634 | - AT1G31350 | - ASK1 | - Y2H |  | - 9 | 1.3E-03 | - [3] |
| - 7 | - At_F0397 | - AT4G12560 | - ASK1_2_more | - Y2H, BIFC | - CPR30 | - 10 | 2.8E-07 | - [5,6] |
| - 8 | - At_F0623 | - AT2G17030b | - ASK1 | - Y2H |  | - 10 | 5.9E-04 | - [3] |
| - 9 | - At_F0102 | - AT5G49610 | - ASK1_2_MORE | - Y2H |  | - 11 | 3.5E-10 | - [4,5] |
| - 10 | - At_F0581 | - AT4G02740a | - ASK2 | - Y2H |  | - 11 | 1.1E-04 | - [3] |
| - 11 | - At_F0115 | - AT1G76920 | - ASK1_2_more | - Y2H |  | - 12 | 4.7E-10 | - [2,3] |
| - 12 | - At_F0214 | - AT4G05460 | - ASK2 | - Y2H |  | - 13 | 3.5E-09 | - [3] |
| - 13 | - Atpeg0040 | - AT4G05460 | - ASK2 | - Y2H |  | - 13 | 3.5E-09 | - [3] |
| - 14 | - At_F0522 | - AT3G62230 | - ASK4 | - Y2H |  | - 13 | 1.0E-05 | - [2] |
| - 15 | - At_F0402 | - AT2G24540 | - ASK1 | - Y2H | - AFR | - 13 | 3.1E-07 | - [3,7] |
| - 16 | - At_F0641 | - AT3G26000 | - ASK1_2 | - Y2H |  | - 13 | 2.1E-03 | - [3] |
| - 17 | - At_F0477 | - AT2G21950 | - ASK1 | - Y2H |  | - 14 | 2.1E-06 | - [3] |
| - 18 | - At_F0559 | - AT4G21510 | - ASK1_2 | - Y2H |  | - 14 | 3.8E-05 | - [3] |
| - 19 | - At_F0563 | - AT3G54650 | - ASK1_2_more | - Y2H, CoIP | - FBL17 | - 14 | 4.8E-05 | - [8,9] |
| - 20 | - At_F0569 | - AT4G02440 | - ASK1_2 | - Y2H, PD | - EID1 | - 14 | 5.9E-05 | - [3,10] |
| - 21 | - At_F0662 | - AT5G49980 | - ASK1_2 | - Y2H, CoIP | - AFB5 | - 14 | 1.4E-02 | - [11] |
| - 22 | - At_F0705 | - AT2G39940 | - ASK1_2_MORE | - Y2H, CoIP | - COI1 | - 14 | 7.3E-01 | - [4,12,13,14] |
| - 23 | - At_F0234 | - AT4G24210 | - ASK1_2_MORE | - Y2H, CoIP | - SLY1 | - 15 | 6.0E-09 | - [15] |
| - 24 | - At_F0283 | - AT1G23780 | - ASK1_2 | - Y2H |  | - 15 | 1.5E-08 | - [3] |
| - 25 | - At_F0297 | - AT5G48170 | - ASK1_2_MORE |  | - SLY2 | - 15 | 1.8E-08 | - [15] |
| - 26 | - At_F0468 | - AT3G61060 | - ASK1_2_more | - Y2H |  | - 15 | 1.5E-06 | - [2,3,12] |
| - 27 | - At_F0474 | - AT5G57360 | - ASK1 | - Y2H, PD | - ZEITLUPE - /ZTL | - 15 | 1.8E-06 | - [16] |
| - 28 | - At_F0542 | - AT2G18915 | - ASK1_2_MORE | - Y2H | - LKP2 | - 15 | 2.0E-05 | - [17] |
| - 29 | - At_F0585 | - AT1G68050 | - ASK1_2_MORE | - Y2H | - FKF | - 15 | 1.4E-04 | - [4,12,17] |
| - 30 | - At_F0508 | - AT1G21410 | - ask1_2_more | - Y2H, CoIP | - SKP2A | - 15 | 7.5E-06 | - [18] |
| - 31 | - At_F0678 | - AT2G02870 | - ASK1_2 | - Y2H |  | - 15 | 5.7E-02 | - [3] |
| - 32 | - At_F0676 | - AT2G42620 | - ASK1 | - Y2H, PD | - MAX2/ORE9 | - 15 | 4.6E-02 | - [19,20] |
| - 33 | - At_F0016 | - AT3G61590 | - ASK1_2_MORE | - Y2H | - Hawiian Skirt | - 16 | 9.3E-12 | - [4,12] |
| - 34 | - At_F0360 | - AT2G02230 | - ASK1_2 | - Y2H |  | - 16 | 1.3E-07 | - [3] |
| - 35 | - Atpeg0078 | - AT2G02230 | - ASK1_2 | - Y2H |  | - 16 | 1.3E-07 | - [3] |
| - 36 | - At_F0267 | - AT1G30950 | - ASK1 | - Y2H | - UFO | - 16 | 1.2E-08 | - [2] |
| - 37 | - At_F0338 | - AT3G60350 | - ASK2_7 | - Y2H | - Arabidillo2 | - 16 | 6.1E-08 | - [21] |
| - 38 | - At_F0451 | - AT2G44900 | - ASK2_7 | - Y2H | - Arabidillo2 | - 16 | 1.0E-06 | - [21] |
| - 39 | - At_F0548 | - AT1G25280 | - ASK1 | - Y2H | - ATTLP10 | - 16 | 2.8E-05 | - [3] |
| - 40 | - At_F0505 | - AT4G08980 | - ASK2 | - Y2H |  | - 16 | 6.6E-06 | - [3] |

**Table S4** (continued)

| - **Number** | - **New_ID** | - **AGI_ID Published** | - **ASKs interacted** | - **Method^a^** | - **Function Known** | - **OG Taxa** | - **E-value** | - **References** |
| --- | --- | --- | --- | --- | --- | --- | --- | --- |
| - 41 | - At_F0541 | - AT5G39250 | - ASK1 | - Y2H |  | - 16 | 1.9E-05 | - [12] |
| - 42 | - At_F0557 | - AT3G63220 | - ASK1 | - Y2H |  | - 16 | 3.4E-05 | - [3] |
| - 43 | - At_F0687 | - AT1G67340 | - ASK1_2_more | - Y2H |  | - 16 | 1.2E-01 | - [2] |
| - 44 | - At_F0211 | - AT5G45360 | - ASK1_2 | - Y2H |  | - 17 | 3.3E-09 | - [3] |
| - 45 | - At_F0302 | - AT4G07400 | - ASK2 | - Y2H | - VFB3 | - 17 | 2.1E-08 | - [1] |
| - 46 | - At_F0443 | - AT1G47056 | - ASK2 | - Y2H | - VFB1 | - 17 | 8.2E-07 | - [1] |
| - 47 | - At_F0570 | - AT4G03190 | - ASK1_2 | - Y2H, CoIP | - AFB1 | - 17 | 6.1E-05 | - [22] |
| - 48 | - At_F0586 | - AT3G62980 | - ASK1_2 | - Y2H, CoIP, 3D | - TIR1 | - 17 | 1.4E-04 | - [22] |
| - 49 | - At_F0626 | - AT3G26810 | - ASK1_2 | - Y2H, CoIP | - AFB2 | - 17 | 8.1E-04 | - [22] |
| - 50 | - At_F0628 | - AT1G12820 | - ASK1_2 | - Y2H, CoIP | - AFB3 | - 17 | 9.6E-04 | - [22] |
| - 51 | - At_F0664 | - AT1G06110 | - ASK1_2_more | - Y2H |  | - 17 | 1.7E-02 | - [2,3] |
| - 52 | - At_F0710 | - AT2G01620 | - ASK1 | - Y2H |  | - 17 | 8.9E-01 | - [3] |
| 53 | At_F0001 | AT1G21760 | ASK1_2_more | Y2H | FBP7 | 18 | 6.6E-14 | [2,3,23] |
| 54 | At_F0416 | AT2G25490 | ASK1_2_more | Y2H, PD | EBF1 | 18 | 4.7E-07 | [2,4,12,24,25,26] |
| 55 | At_F0442 | AT5G25350 | ASK1_2 | Y2H, PD | EBF2 | 18 | 8.0E-07 | [24,25,26] |
| 56 | At_F0510 | AT1G55000 | ASK1_2_more | Y2H |  | 18 | 7.6E-06 | - [2] |
| 57 | At_F0147 | AT4G19940 | ASK14_16 | Y2H |  | 0 | 9.8E-10 | [5] |
| 58 | At_F0459 | AT3G22650 | ASK1_2_MORE | Y2H | CEG | 0 | 1.2E-06 | [5] |
| 59 | At_F0024 | At_F0024 | ASK1_2_more | Y2H |  | 2 | 2.2E-11 | - [2] |
| 60 | At_F0086 | At_F0086 | ASK11 | Y2H |  | 2 | 2.5E-10 | - [2,4] |
| 61 | At_F0132 | At_F0132 | ASK13_14 | Y2H |  | 2 | 7.2E-10 | - [4] |
| 62 | At_F0247 | At_F0247 | ASK14 | Y2H |  | 2 | 8.2E-09 | [5] |
| 63 | At_F0320 | At_F0320 | ASK4 | Y2H |  | 2 | 3.3E-08 | - [2] |
| 64 | At_F0391 | At_F0391 | ASK4 | Y2H |  | 2 | 2.6E-07 | - [2] |
| 65 | At_F0421 | At_F0421 | ASK1 | Y2H | ATTLP9 | 2 | 5.0E-07 | [27] |
| 66 | At_F0428 | At_F0428 | ASK16 | Y2H |  | 2 | 5.7E-07 | - [2] |
| 67 | At_F0449 | At_F0449 | ASK1_2_11_13 | Y2H |  | 2 | 9.2E-07 | - [2] |
| 68 | At_F0467 | At_F0467 | ASK1_2 | Y2H |  | 2 | 1.5E-06 | [3] |
| 69 | At_F0531 | At_F0531 | ASK1 | Y2H |  | 2 | 1.6E-05 | [3] |
| 70 | At_F0680 | At_F0680 | ASK2_9_14 | Y2H |  | 2 | 5.7E-02 | [5] |
| 71 | At_F0704 | At_F0704 | ASK4 | Y2H |  | 2 | 6.1E-01 | - [2] |
| 72 | At_F0627 | At_F0627 | ASK1_2 | Y2H |  | 3 | 8.6E-04 | [3] |

^a^BiFC: bimolecular fluorescence complementation ; Y2H: Yeast-two hybrid; CoIP: Co-immunoprecipitation; PD: In-vitro pull down;

**References for Table S4:**

1. Schwager KM, Calderon-Villalobos LI, Dohmann EM, Willige BC, Knierer S, et al. (2007) Characterization of the *VIER F-BOX PROTEINE* genes from *Arabidopsis* reveals their importance for plant growth and development. Plant Cell 19: 1163-1178.

2. Gagne JM, Downes BP, Shiu SH, Durski AM, Vierstra RD (2002) The F-Box subunit of the SCF E3 complex is encoded by a diverse superfamily of genes in *Arabidopsis*. Proc Natl Acad Sci USA 99: 11519-11524.

3. Risseeuw EP, Daskalchuk TE, Banks TW, Liu E, Cotelesage J, et al. (2003) Protein interaction analysis of SCF ubiquitin E3 ligase subunits from *Arabidopsis*. Plant J 34: 753-767.

4. Takahashi N, Kuroda H, Kuromori T, Hirayama T, Seki M, et al. (2004) Expression and interaction analysis of *Arabidopsis* *Skp1*-related genes. Plant Cell Physiol 45: 83-91.

5. Wang L, Dong L, Zhang Y, Wu W, Deng X, et al. (2004) Genome-wide analysis of *S-Locus F-Box-Like* genes in *Arabidopsis thaliana*. Plant Mol Biol 56: 929-945.

6. Gou M, Su N, Zheng J, Huai J, Wu G, et al. (2009) An *F-Box* gene, *CPR30*, functions as a negative regulator of the defense response in *Arabidopsis*. Plant J 60: 757-770.

7. Harmon FG, Kay SA (2003) The F-Box protein AFR is a positive regulator of phytochrome A-mediated light signaling. Curr Biol 13: 2091-2096.

8. Gusti A, Baumberger N, Nowack M, Pusch S, Eisler H, et al. (2009) The *Arabidopsis thaliana* F-Box protein FBL17 is essential for progression through the second mitosis during pollen development. PLoS ONE 4: e4780.

9. Kim HJ, Oh SA, Brownfield L, Hong SH, Ryu H, et al. (2008) Control of plant germline proliferation by SCF^FBL17^ degradation of cell cycle inhibitors. Nature 455: 1134-1137.

10. Dieterle M, Zhou YC, Schafer E, Funk M, Kretsch T (2001) EID1, an F-Box protein involved in phytochrome A-specific light signaling. Genes Dev 15: 939-944.

11. Gray WM, del Pozo JC, Walker L, Hobbie L, Risseeuw E, et al. (1999) Identification of an SCF ubiquitin-ligase complex required for auxin response in *Arabidopsis thaliana*. Genes Dev 13: 1678-1691.

12. Kuroda H, Takahashi N, Shimada H, Seki M, Shinozaki K, et al. (2002) Classification and expression analysis of *Arabidopsis* F-Box-containing protein genes. Plant Cell Physiol 43: 1073-1085.

13. Sheard LB, Tan X, Mao H, Withers J, Ben-Nissan G, et al. (2010) Jasmonate perception by inositol-phosphate-potentiated COI1-JAZ co-receptor. Nature 468: 400-405.

14. Xu L, Liu F, Lechner E, Genschik P, Crosby WL, et al. (2002) The SCF^COI1^ ubiquitin-ligase complexes are required for jasmonate response in *Arabidopsis*. Plant Cell 14: 1919-1935.

15. Fu X, Richards DE, Fleck B, Xie D, Burton N, et al. (2004) The *Arabidopsis* mutant sleepy1^gar2-1^ protein promotes plant growth by increasing the affinity of the SCF^SLY1^ E3 ubiquitin ligase for DELLA protein substrates. Plant Cell 16: 1406-1418.

16. Han L, Mason M, Risseeuw EP, Crosby WL, Somers DE (2004) Formation of an SCF^ZTL^ complex is required for proper regulation of circadian timing. Plant J 40: 291-301.

17. Yasuhara M, Mitsui S, Hirano H, Takanabe R, Tokioka Y, et al. (2004) Identification of ASK and clock-associated proteins as molecular partners of LKP2 (LOV kelch protein 2) in *Arabidopsis*. J Exp Bot 55: 2015-2027.

18. Jurado S, Diaz-Trivino S, Abraham Z, Manzano C, Gutierrez C, et al. (2008) SKP2A, an F-Box protein that regulates cell division, is degraded via the ubiquitin pathway. Plant J 53: 828-841.

19. Stirnberg P, Furner IJ, Ottoline Leyser HM (2007) MAX2 participates in an SCF complex which acts locally at the node to suppress shoot branching. Plant J 50: 80-94.

20. Woo HR, Chung KM, Park JH, Oh SA, Ahn T, et al. (2001) ORE9, an F-Box protein that regulates leaf senescence in *Arabidopsis*. Plant Cell 13: 1779-1790.

21. Coates JC, Laplaze L, Haseloff J (2006) Armadillo-related proteins promote lateral root development in *Arabidopsis*. Proc Natl Acad Sci USA 103: 1621-1626.

22. Dharmasiri N, Dharmasiri S, Weijers D, Lechner E, Yamada M, et al. (2005) Plant development is regulated by a family of auxin receptor F-Box proteins. Dev Cell 9: 109-119.

23. Calderon-Villalobos LI, Nill C, Marrocco K, Kretsch T, Schwechheimer C (2007) The evolutionarily conserved *Arabidopsis thaliana* F-Box protein AtFBP7 is required for efficient translation during temperature stress. Gene 392: 106-116.

24. Gagne JM, Smalle J, Gingerich DJ, Walker JM, Yoo SD, et al. (2004) *Arabidopsis* EIN3-binding F-Box 1 and 2 form ubiquitin-protein ligases that repress ethylene action and promote growth by directing EIN3 degradation. Proc Natl Acad Sci USA 101: 6803-6808.

25. Guo H, Ecker JR (2003) Plant responses to ethylene gas are mediated by SCF^EBF1/EBF2^-dependent proteolysis of EIN3 transcription factor. Cell 115: 667-677.

26. Potuschak T, Lechner E, Parmentier Y, Yanagisawa S, Grava S, et al. (2003) EIN3-dependent regulation of plant ethylene hormone signaling by two *Arabidopsis* F-Box proteins: EBF1 and EBF2. Cell 115: 679-689.

27. Lai CP, Lee CL, Chen PH, Wu SH, Yang CC, et al. (2004) Molecular analyses of the *Arabidopsis* TUBBY-like protein gene family. Plant Physiol 134: 1586-1597.
