# Supplementary material for: Phylogenetic Comparison of F-Box (FBX) Gene Superfamily within the Plant Kingdom Reveals Divergent Evolutionary Histories Indicative of Genomic Drift
Source: PLoS One. 2011 Jan 28;6(1):e16219. doi: 10.1371/journal.pone.0016219 (PMC3030570; doi:10.1371/journal.pone.0016219)
Supplement: Table S5 — The enrichment of various domain (dmn) combinations in the putative substrate recruitment module of FBX proteins. p indicates the enrichment probability of FBX proteins with a specific domain/domain combination as compared to that for the average number of FBX proteins. In total, 3,269 domains/domain combinations (including no prediction group) were identified from 10,811 FBX proteins. (DOC) [file pone.0016219.s005.doc]

**Table S5.** The enrichment of various domain (dmn) combinations in the putative substrate recruitment module of FBX proteins.

| dmn | #FBX proteins w/ dmn | #average FBX proteins w/ an assigned dmn | #FBX proteins w/o dmn | #average FBX proteins w/o an assigned dmn | Fisher's exact test (*p*) |
| --- | --- | --- | --- | --- | --- |
| No prediction | 3589 | 3 | 7222 | 10808 | < 2.2e-16 |
| Rare | 3878 | 3 | 6933 | 10808 |  |
| FBA_CLAN | 855 | 3 | 9956 | 10808 | < 2.2e-16 |
| kelch_CLAN | 578 | 3 | 10233 | 10808 | < 2.2e-16 |
| DUF295 | 370 | 3 | 10441 | 10808 | < 2.2e-16 |
| LRR_CLAN+FBD | 312 | 3 | 10499 | 10808 | < 2.2e-16 |
| FBD | 285 | 3 | 10526 | 10808 | < 2.2e-16 |
| LRR_CLAN | 253 | 3 | 10558 | 10808 | < 2.2e-16 |
| Tubby_c_CLAN+DUF3527 | 117 | 3 | 10694 | 10808 | < 2.2e-16 |
| PRANC | 76 | 3 | 10735 | 10808 | < 2.2e-16 |
| TPR_1_CLAN | 61 | 3 | 10750 | 10808 | 2.2E-15 |
| FBA_CLAN+kelch_CLAN | 60 | 3 | 10751 | 10808 | 4.2E-15 |
| Beta_propeller_CLAN | 47 | 3 | 10764 | 10808 | 1.8E-11 |
| kelch_CLAN+PAS | 41 | 3 | 10770 | 10808 | 7.8E-10 |
| TPR_1_CLAN+zf-MYND | 28 | 3 | 10783 | 10808 | 2.3E-06 |
| FBA_CLAN+DUF1618 | 20 | 3 | 10791 | 10808 | 2.4E-04 |
| LysM | 18 | 3 | 10793 | 10808 | 7.4E-04 |
| AAA_CLAN | 17 | 3 | 10794 | 10808 | 1.3E-03 |
| Elongin_A | 17 | 3 | 10794 | 10808 | 1.3E-03 |
| kelch_CLAN+Beta_propeller_CLAN | 16 | 3 | 10795 | 10808 | 2.2E-03 |
| Cupin_clan | 16 | 3 | 10795 | 10808 | 2.2E-03 |
| DUF1618 | 16 | 3 | 10795 | 10808 | 2.2E-03 |
| Actin_ATPase_CLA | 15 | 3 | 10796 | 10808 | 3.8E-03 |
| SMI1_KNR4 | 15 | 3 | 10796 | 10808 | 3.8E-03 |
| kelch_CLAN+Glyoxal_oxid_N | 14 | 3 | 10797 | 10808 | 6.3E-03 |
| SMI1_KNR4+DUF525 | 14 | 3 | 10797 | 10808 | 6.3E-03 |
| FIST_C | 14 | 3 | 10797 | 10808 | 6.3E-03 |
| FBA_CLAN+Rod_C | 13 | 3 | 10798 | 10808 | 1.1E-02 |
| Flavi_capsid | 13 | 3 | 10798 | 10808 | 1.1E-02 |
| Beta_propeller_CLAN+DUF295 | 12 | 3 | 10799 | 10808 | 1.8E-02 |
| FBA_CLAN+PRANC | 11 | 3 | 10800 | 10808 | 2.9E-02 |
| AAA_CLAN+zf-CW | 10 | 3 | 10801 | 10808 | 4.6E-02 |
