# Supplementary material for: Phylogenetic Comparison of F-Box (FBX) Gene Superfamily within the Plant Kingdom Reveals Divergent Evolutionary Histories Indicative of Genomic Drift
Source: PLoS One. 2011 Jan 28;6(1):e16219. doi: 10.1371/journal.pone.0016219 (PMC3030570; doi:10.1371/journal.pone.0016219)
Supplement: Table S6 — The enrichment comparison of domain (dmn) combinations in the putative substrate recruitment module of the translated protein products from FBX pseudogenes and protein-coding genes. p (left) indicates the domain enrichment probability in the translated protein products from FBX pseudogenes. p (right) indicates the domain enrichment probability in the translated protein products from protein-coding genes. (DOC) [file pone.0016219.s006.doc]

**Table S6.** The enrichment comparison of domain (dmn) combinations in the putative substrate recruitment module of the translated protein products from *FBX* pseudogenes and protein-coding genes.

| dmn | # *FBX* pseudogenes w/ dmn | # protein-coding genes w/ dmn | # *FBX* pseudogenes w/o dmn | # protein-coding genes w/o dmn | Fisher's exact test | |
| --- | --- | --- | --- | --- | --- | --- |
|  |  |  |  |  | *p* (left) | *p* (right) |
| No prediction* | 866 | 2723 | 1270 | 5952 | 1.1E-15 | 1.0E+00 |
| Rare | 794 | 3084 | 1342 | 5591 | 8.5E-02 | 9.2E-01 |
| FBA_CLAN | 176 | 679 | 1960 | 7996 | 2.8E-01 | 7.5E-01 |
| kelch_CLAN** | 34 | 544 | 2102 | 8131 | 1.0E+00 | < 2.2e-16 |
| DUF295** | 50 | 320 | 2086 | 8355 | 1.0E+00 | 9.0E-04 |
| LRR_CLAN+FBD** | 41 | 271 | 2095 | 8404 | 1.0E+00 | 1.2E-03 |
| FBD | 54 | 231 | 2082 | 8444 | 6.6E-01 | 4.0E-01 |
| LRR_CLAN | 49 | 204 | 2087 | 8471 | 5.9E-01 | 4.8E-01 |
| Tubby_c_CLAN+DUF3527** | 4 | 113 | 2132 | 8562 | 1.0E+00 | 1.9E-07 |
| PRANC | 13 | 63 | 2123 | 8612 | 7.6E-01 | 3.4E-01 |
| TPR_1_CLAN** | 6 | 55 | 2130 | 8620 | 9.9E-01 | 3.0E-02 |
| FBA_CLAN+kelch_CLAN | 8 | 52 | 2128 | 8623 | 9.3E-01 | 1.4E-01 |
| Beta_propeller_CLAN | 6 | 41 | 2130 | 8634 | 9.2E-01 | 1.5E-01 |
| kelch_CLAN+PAS** | 0 | 41 | 2136 | 8634 | 1.0E+00 | 1.2E-04 |
| TPR_1_CLAN+zf-MYND** | 0 | 28 | 2136 | 8647 | 1.0E+00 | 2.1E-03 |
| FBA_CLAN+DUF1618 | 2 | 18 | 2134 | 8657 | 9.3E-01 | 2.1E-01 |
| LysM** | 0 | 18 | 2136 | 8657 | 1.0E+00 | 1.9E-02 |
| AAA_CLAN | 3 | 14 | 2133 | 8661 | 6.8E-01 | 5.6E-01 |
| Elongin_A | 3 | 14 | 2133 | 8661 | 6.8E-01 | 5.6E-01 |
| kelch_CLAN+Beta_propeller_CLAN** | 0 | 16 | 2136 | 8659 | 1.0E+00 | 2.9E-02 |
| Cupin_clan | 2 | 14 | 2134 | 8661 | 8.5E-01 | 3.6E-01 |
| DUF1618 | 1 | 15 | 2135 | 8660 | 9.7E-01 | 1.5E-01 |
| Actin_ATPase_CLA | 3 | 12 | 2133 | 8663 | 5.9E-01 | 6.6E-01 |
| SMI1_KNR4 | 5 | 10 | 2131 | 8665 | 1.6E-01 | 9.4E-01 |
| kelch_CLAN+Glyoxal_oxid_N | 1 | 13 | 2135 | 8662 | 9.5E-01 | 2.0E-01 |
| SMI1_KNR4+DUF525** | 0 | 14 | 2136 | 8661 | 1.0E+00 | 4.6E-02 |
| FIST_C | 3 | 11 | 2133 | 8664 | 5.4E-01 | 7.1E-01 |
| FBA_CLAN+Rod_C | 2 | 11 | 2134 | 8664 | 7.6E-01 | 5.1E-01 |
| Flavi_capsid | 2 | 11 | 2134 | 8664 | 7.6E-01 | 5.1E-01 |
| Beta_propeller_CLAN+DUF295 | 1 | 11 | 2135 | 8664 | 9.3E-01 | 2.8E-01 |
| FBA_CLAN+PRANC | 2 | 9 | 2134 | 8666 | 6.7E-01 | 6.3E-01 |
| AAA_CLAN+zf-CW | 2 | 8 | 2134 | 8667 | 6.2E-01 | 6.9E-01 |
| Ste50p-SAM | 3 | 7 | 2133 | 8668 | 3.1E-01 | 8.8E-01 |

The single asterisk indicates the significant enrichment in pseudogenes and the double asterisks indicate the significant enrichment in protein-coding genes (*p*< 0.05). *P* (left) indicates the domain enrichment probability in the translated protein products from FBX pseudogenes. *P* (right) indicates the domain enrichment probability in the translated protein products from protein-coding genes.
