# Supplementary material for: Phylogenetic Comparison of F-Box (FBX) Gene Superfamily within the Plant Kingdom Reveals Divergent Evolutionary Histories Indicative of Genomic Drift
Source: PLoS One. 2011 Jan 28;6(1):e16219. doi: 10.1371/journal.pone.0016219 (PMC3030570; doi:10.1371/journal.pone.0016219)
Supplement: Table S8 — Wilcoxon rank sum test (one tailed) of Ka/Ks values of the LTS protein-coding FBX genes (LTSP), STS protein-coding FBX genes (STSP) and FBX pseudogenes (ψ in each plant species. (DOC) [file pone.0016219.s008.doc]

**Table S8.** Wilcoxon rank sum test (one tailed) of *Ka/Ks* values of the LTS protein-coding *FBX* genes (LTSP), STS protein-coding *FBX* genes (STSP) and *FBX* pseudogenes ( in each plant species.

| **Species** | **Goldman and Yang's method** | | | **Nei and Gojobori's method** | | |
| --- | --- | --- | --- | --- | --- | --- |
|  | LTSP < STSP | LTSP < ψ | STSP < ψ | LTSP < STSP | LTSP < ψ | STSP < ψ |
| *Al* | < 2.2E-16* | < 2.2E-16* | 2.5E-04* | < 2.2E-16* | < 2.2E-16* | 1.1E-04* |
| *At* | < 2.2E-16* | < 2.2E-16* | 1.9E-01 | < 2.2E-16* | < 2.2E-16* | 2.0E-01 |
| *Bd* | < 2.2E-16* | < 2.2E-16* | 1.3E-11* | < 2.2E-16* | < 2.2E-16* | 3.0E-12* |
| *Cp* | 1.4E-09* | 3.6E-08* | 7.2E-01 | 7.4E-10* | 6.9E-08* | 7.8E-01 |
| *Cr* | 9.1E-03 | 5.0E-01 | 9.4E-01 | 2.3E-02 | 8.0E-01 | 9.7E-01 |
| *Cs* | 9.5E-10* | 2.3E-04* | 3.0E-01 | 2.1E-10* | 3.7E-04* | 4.6E-01 |
| *Gm* | < 2.2E-16* | < 2.2E-16* | 1.5E-05* | < 2.2E-16* | < 2.2E-16* | 2.3E-04* |
| *Me* | < 2.2E-16* | 2.2E-05* | 9.4E-01 | < 2.2E-16* | 1.4E-04* | 8.9E-01 |
| *Mg* | 1.9E-05* | 1.2E-09* | 1.0E-03 | 1.1E-04* | 1.4E-08* | 1.4E-03 |
| *Mt* | 2.1E-09* | 1.6E-15* | 4.3E-04* | 4.7E-10* | 1.1E-15* | 7.5E-04* |
| *Os* | < 2.2E-16* | < 2.2E-16* | 1.0E-02 | < 2.2E-16* | < 2.2E-16* | 8.0E-03 |
| *Pp* | 6.4E-09* | 1.7E-03 | 3.9E-01 | 5.5E-07* | 1.8E-03 | 3.8E-01 |
| *Pt* | < 2.2E-16* | 2.5E-12* | 2.1E-02 | < 2.2E-16* | 9.5E-13* | 1.8E-02 |
| *Rc* | 2.7E-16* | 1.3E-11* | 2.5E-01 | 4.6E-14* | 1.3E-10* | 3.4E-01 |
| *Sb* | < 2.2E-16* | < 2.2E-16* | 1.5E-03 | < 2.2E-16* | < 2.2E-16* | 1.5E-03 |
| *Sm* | < 2.2E-16* | < 2.2E-16* | 6.9E-04* | < 2.2E-16* | < 2.2E-16* | 9.6E-04* |
| *Vv* | 2.7E-06* | 5.1E-08* | 9.7E-03 | 1.3E-06* | 2.2E-07* | 4.2E-02 |
| *Zm* | < 2.2E-16* | < 2.2E-16* | 2.1E-05* | < 2.2E-16* | < 2.2E-16* | 3.1E-05* |

**p* <0.001.
