# Supplementary material for: Phylogenetic Comparison of F-Box (FBX) Gene Superfamily within the Plant Kingdom Reveals Divergent Evolutionary Histories Indicative of Genomic Drift
Source: PLoS One. 2011 Jan 28;6(1):e16219. doi: 10.1371/journal.pone.0016219 (PMC3030570; doi:10.1371/journal.pone.0016219)
Supplement: Table S9 — Wilcoxon rank sum test (one tailed) of Ka/Ks values from FBXD and Δ FBXD identified within the LTS protein-coding FBX gene (LTSP), STS protein-coding FBX gene (STSP), and FBX pseudogene (ψ groups in each of the 18 plant species. (DOC) [file pone.0016219.s009.doc]

**Table S9.** Wilcoxon rank sum test (one tailed) of *Ka/Ks* values from *FBXD* and Δ*FBXD* identified within the LTS protein-coding *FBX* gene (LTSP), STS protein-coding *FBX* gene (STSP), and *FBX* pseudogene ( groups in each of the 18 plant species.

| **Species** | ***FBXD* (Goldman and Yang's method)** | | | **Δ*FBXD* (Goldman and Yang's method)** | | |
| --- | --- | --- | --- | --- | --- | --- |
|  | LTSP < STSP | LTSP < ψ | STSP < ψ | LTSP < STSP | LTSP < ψ | STSP < ψ |
| *Al* | < 2.2E-16* | < 2.2E-16* | 1.9E-01 | < 2.2E-16* | < 2.2E-16* | 1.3E-01 |
| *At* | < 2.2E-16* | < 2.2E-16* | 2.8E-01 | < 2.2E-16* | < 2.2E-16* | 9.9E-01 |
| *Bd* | 7.7E-05* | 4.7E-04* | 5.7E-01 | 8.4E-12* | < 2.2E-16* | < 2.2e-16* |
| *Cp* | 9.8E-06* | 3.0E-08* | 4.2E-02 | 4.0E-09* | 2.1E-06* | 9.7E-01 |
| *Cr* | 4.2E-01 | 5.5E-01 | 7.7E-01 | 8.6E-03 | 2.4E-01 | 9.3E-01 |
| *Cs* | 5.3E-09* | 2.3E-01 | 1.0E+00 | 8.0E-07* | 4.9E-04* | 1.2E-01 |
| *Gm* | 2.2E-13* | 2.8E-14* | 4.7E-02 | < 2.2E-16* | < 2.2E-16* | 2.5E-01 |
| *Me* | 3.4E-10* | 7.6E-02 | 9.0E-01 | < 2.2E-16* | 2.7E-05* | 9.3E-01 |
| *Mg* | 8.6E-10* | 1.6E-05* | 7.4E-01 | 1.1E-02 | 1.8E-05* | 2.0E-03 |
| *Mt* | 5.3E-08* | 1.2E-05* | 4.7E-01 | 3.5E-13* | 2.5E-13* | 1.0E-01 |
| *Os* | 2.1E-04* | 6.8E-06* | 2.7E-02 | 1.3E-11* | < 2.2E-16* | 1.1E-07* |
| *Pp* | 1.2E-06* | 7.3E-02 | 8.1E-01 | 1.6E-06* | 5.7E-04* | 1.1E-01 |
| *Pt* | 8.3E-08* | 3.9E-09* | 7.3E-04* | < 2.2E-16* | 8.3E-10* | 2.2E-01 |
| *Rc* | 3.1E-08* | 8.0E-04* | 8.8E-01 | 4.7E-16* | 1.4E-11* | 1.8E-01 |
| *Sb* | 3.0E-05* | 2.4E-03 | 6.4E-01 | < 2.2E-16* | < 2.2E-16* | 1.4E-03 |
| *Sm* | 4.3E-07* | 2.7E-08* | 4.8E-03 | < 2.2E-16* | < 2.2E-16* | 4.7E-07* |
| *Vv* | 5.5E-05* | 1.5E-01 | 9.4E-01 | 1.6E-09* | 1.5E-08* | 1.9E-01 |
| *Zm* | 4.1E-02 | 1.6E-02 | 1.8E-01 | < 2.2E-16* | < 2.2E-16* | 2.1E-04* |

**p* <0.001.
