# Supplementary material for: Phylogenetic Comparison of F-Box (FBX) Gene Superfamily within the Plant Kingdom Reveals Divergent Evolutionary Histories Indicative of Genomic Drift
Source: PLoS One. 2011 Jan 28;6(1):e16219. doi: 10.1371/journal.pone.0016219 (PMC3030570; doi:10.1371/journal.pone.0016219)
Supplement: Table S10 — Enrichment in various microarray datasets and functional prediction of expressed FBX genes from LTS and STS groups of A. thaliana . (DOC) [file pone.0016219.s010.doc]

**Table S10.** Enrichment in various microarray datasets and functional prediction of expressed *FBX* genes from LTS and STS groups of

*A. thaliana.*

| **(A) Both LTS and STS genes are enriched in the same experiment (*p*<0.05).** | | | | | |
| --- | --- | --- | --- | --- | --- |
| **Group** | **Slide_name** | **# genes** | ***p*-value*** | **Exp_ID**** | **Category** |
| LTS | Finch-Savage_1-22_SD2_Rep2_ATH1 | ||||||||||||||||||||||||||||||||| 67/82 | 8.4E-03 | 69 | development_seed_dormancy |
| STS | Finch-Savage_1-45_PDLN_Rep1_ATH1 | ||||||||||||||||||||||||||||||||| 66/177 | 1.8E-11 | 69 | development_seed_dormancy |
| LTS | Holman_1-21_ABA-IM_Rep3_ATH1 | |||||||||||||||||||||||||||||| 61/88 | 4.5E-02 | 339 | development_seed_dormancy |
| STS | Holman_1-21_ABA-IM_Rep3_ATH1 | |||||||||||||||||||||||| 49/194 | 5.3E-07 | 339 | development_seed_dormancy |
| LTS | Carrera Begua_1-33_cts1-24hr-dormancy_Rep3_ATH1 | |||||||||||||||||||||||||||||||| 65/84 | 1.5E-02 | 61 | development_shoot_dormancy |
| STS | Carrera Begua_1-33_cts1-24hr-dormancy_Rep3_ATH1 | |||||||||||||||||||||||||| 53190 | 5.5E-08 | 61 | development_shoot_dormancy |
| LTS | Dubos_A-2-wtc_Rep1 | ||||||||||||||||||||||||||||||||||||| 75/74 | 4.6E-04 | 92 | development_dormancy |
| STS | Dubos_A-6-5kc_Rep2 | ||||||||||||| 27/216 | 1.5E-02 | 92 | development_dormancy |
| LTS | RIKEN-PRESTON0B | ||||||||||||||||||||||||||||||| 63/86 | 2.7E-02 | 195 | development_seed_germination |
| STS | RIKEN-PRESTON0A | ||||||||||||| 27/216 | 1.5E-02 | 195 | development_seed_germination |
| LTS | Tatematsu_1-8_cho1-dry-seeds_Rep2_ATH1 | |||||||||||||||||||||||||||||||| 64/85 | 2.1E-02 | 497 | development_seed_germination |
| STS | Tatematsu_1-1_WT-Col-dry-seeds_Rep1_ATH1 | ||||||||||||| 26/217 | 2.2E-02 | 497 | development_seed_germination |
| LTS | Tatematsu_3-27_Cvi-seeds-1hr-imbibition_Rep3_ATH1 | ||||||||||||||||||||||||||||||||||| 71/78 | 2.2E-03 | 499 | development_seed_germination |
| STS | Tatematsu_3-27_Cvi-seeds-1hr-imbibition_Rep3_ATH1 | ||||||||||||||| 31/212 | 3.3E-03 | 499 | development_seed_germination |
| LTS | Lindsey_1-14_torpedo-root_Rep1_ATH1 | |||||||||||||||||||||||||||||||||||| 72/77 | 1.5E-03 | 55 | development_embryo |
| STS | Lindsey_1-14_torpedo-root_Rep1_ATH1 | ||||||||||||||||||||||||||||||||||||||||||||||||||||||||||||| 123/120 | 1.7E-31 | 55*** | development_embryo |
| LTS | Fukuda_1-11_10A_Rep1_ATH1 | |||||||||||||||||||||||||||||||||| 69/80 | 4.4E-03 | 361 | development_cell wall |
| STS | Fukuda_1-11_10A_Rep1_ATH1 | |||||||||||||||| 33/210 | 1.4E-03 | 361 | development_cell wall |
| LTS | A-2-Brown-2day_high | ||||||||||||||||||||||||||||||||| 66/83 | 1.1E-02 | 80 | development_cell wall |
| STS | A-5-Brown-5day_low | ||||||||||||| 27/216 | 1.5E-02 | 80 | development_cell wall |
| LTS | RIKEN-NAKABAYASHI1B | |||||||||||||||||||||||||||||||| 64/85 | 2.1E-02 | 183 | hormone |
| STS | RIKEN-NAKABAYASHI1A | |||||||||||| 24/219 | 4.3E-02 | 183 | hormone |
| LTS | Sakakibara_5-19_LL-1ox_Rep1_ATH1 | |||||||||||||||||||||||||||||||||||||||||||||| 92/57 | 6.9E-08 | 420 | circadian |
| STS | Sakakibara_5-19_LL-1ox_Rep1_ATH1 | |||||||||||||||| 32/211 | 2.2E-03 | 420 | circadian |
| LTS | Gifford_1-30_Peri-Continuous-KNO3_Rep3_ATH1 | |||||||||||||||||||||||||||||||||| 69/80 | 4.4E-03 | 490 | nutrient_nitrogen |
| STS | Gifford_1-89_Whole-Roots-Incubated-Transitory-KNO3_Rep2_ATH1 | ||||||||||||||||||||||||||||||||||||||||||||||||||||||||| 114/129 | 8.0E-28 | 490 | nutrient_nitrogen |
| LTS | Swidzinski_1-6_Heat_Rep3_ATH1 | |||||||||||||||||||||||||||||||||||| 72/77 | 1.5E-03 | 30 | pathogen |
| STS | Swidzinski_1-4_Heat_Rep1_ATH1 | ||||||||||||||| 30/213 | 4.9E-03 | 30 | pathogen |
| LTS | Broadley_2-5_caerulescens_Rep1_ATH1 | |||||||||||||||||||||||||||||||| 64/85 | 2.1E-02 | 104 | stress_mineral pehnotype |
| STS | Broadley_2-5_caerulescens_Rep1_ATH1 | ||||||||||||||||||||||||||||| 58/185 | 2.8E-09 | 104 | stress_mineral pehnotype |
| LTS | Newbury_1-11_Halleri-highZn-leaves(HLH)_Rep2_ATH1 | ||||||||||||||||||||||||||||||||||||||| 78/71 | 1.3E-04 | 85 | stress_Zn |
| STS | Newbury_1-11_Halleri-highZn-leaves(HLH)_Rep2_ATH1 | ||||||||||||||||||||||||||||| 58/185 | 2.8E-09 | 85 | stress_Zn |
| LTS | Hampton_2-2_Col-0_Rep2_ATH1 | ||||||||||||||||||||||||||||||||| 66/83 | 1.1E-02 | 384 | stress_Cs |
| STS | Hampton_2-2_Col-0_Rep2_ATH1 | ||||||||||||||||||||||||||||||||||||||||||||||||||||||||||| 118/125 | 2.0E-29 | 384 | stress_Cs |
| LTS | Guy_1-38_60min-40C_Rep2_ATH1 | ||||||||||||||||||||||||||||||||||| 71/78 | 2.2E-03 | 404 | stress_cold |
| STS | Guy_1-38_60min-40C_Rep2_ATH1 | ||||||||||||||||||||||||||||||||||||||||||||||||| 98/145 | 8.9E-22 | 404 | stress_cold |
| LTS | Average | ||||||||||||||||||||||| 46/103 |  |  |  |
| STS | Average | |||||| 13/230 |  |  |  |

| **(B) LTS and STS genes are not enriched in the same experiment (*p*<0.05).** | | | | | |
| --- | --- | --- | --- | --- | --- |
| **Group** | **Slide_name** | **# genes** | ***p*-value*** | **Exp_ID**** | **Category** |
| LTS | Edwards_2-13_Far-Red-50hr_Rep1_ATH1 | |||||||||||||||||||||||||||||||| 64/85 | 2.1E-02 | 196 | circadian |
| LTS | Jaffe_1-3_Wt-Col_Rep2_ATH1 | ||||||||||||||||||||||||||||||| 62/87 | 3.5E-02 | 477 | development |
| LTS | Aldridge_1-4_Prolonged-induction_Rep2_ATH1 | |||||||||||||||||||||||||||||| 61/88 | 4.5E-02 | 341 | development |
| LTS | Lopez-Juez_1-1_Shoot-apex-dark_Rep1_ATH1 | |||||||||||||||||||||||||||||||| 64/85 | 2.1E-02 | 426 | development |
| LTS | Li_1-6_prl1_Rep2_ATH1 | ||||||||||||||||||||||||||||||| 62/87 | 3.5E-02 | 419 | development |
| STS | Murray_2-10_T19-APH_Rep1_ATH1 | ||||||||||||||||| 34/209 | 9.4E-04 | 360 | development_cell cyle |
| LTS | Campb-327-MYB61-noSuc-Rep2 | |||||||||||||||||||||||||||||| 61/88 | 4.5E-02 | 14 | development_cell wall |
| LTS | Eland_2-1_A1-eland-ch1 | |||||||||||||||||||||||||||||||| 64/85 | 2.1E-02 | 117 | development_cell wall |
| LTS | Turner_A-8-Turne-WT-Base2_SLD | |||||||||||||||||||||||||||||| 61/88 | 4.5E-02 | 54 | development_cell wall |
| STS | A1-WILLA-CON | |||||||||||| 24/219 | 4.3E-02 | 27 | development_cell wall |
| STS | Murray_3-1_D1-GROWTH_Rep1_ATH1 | |||||||||||||||||| 36/207 | 3.8E-04 | 381 | development_cell wall |
| LTS | ATGE_26_B | |||||||||||||||||||||||||||||||| 64/85 | 2.1E-02 | 150 | development_leaf |
| LTS | Vizcay-Barrena_1-3_Ler-young_Rep1_ATH1 | ||||||||||||||||||||||||||||||| 62/87 | 3.5E-02 | 23 | development_reproduction |
| STS | Honys_2-6_Col-TF2_Rep2_ATH1 | |||||||||||||||| 32/211 | 2.2E-03 | 412 | development_reproduction |
| STS | Honys_UNM2_SLD | ||||||||||||||||||||||||||||||||| 67/176 | 9.2E-12 | 48*** | development_reproduction_pollen |
| STS | Twell_1-2_pollen_Rep1_ATH1 | |||||||||||| 25/218 | 3.1E-02 | 6 | development_reproduction_pollen |
| LTS | ATGE_98_B | |||||||||||||||||||||||||||||| 61/88 | 4.5E-02 | 151 | development_root |
| LTS | Gan_1-1_wildtype-nitrate-minus(WNM)_Rep1_ATH1 | ||||||||||||||||||||||||||||||| 62/87 | 3.5E-02 | 103 | development_root |
| LTS | ATGE_27_B | |||||||||||||||||||||||||||||| 61/88 | 4.5E-02 | 153 | development_shoot |
| LTS | Ward_A2-WARD-ax1_SLD | |||||||||||||||||||||||||||||| 61/88 | 4.5E-02 | 45 | development_shoot |
| LTS | Tatematsu_2-1_axillary-buds-before-decapitation_Rep1_ATH1 | |||||||||||||||||||||||||||||| 61/88 | 4.5E-02 | 498 | development_shoot |
| LTS | Josse_1-9_WT-24hr_Rep3_ATH1 | |||||||||||||||||||||||||||||||| 65/84 | 1.5E-02 | 505 | hormone respnose |
| LTS | AtGen_D-1_1-DL_REP1_ATH1 | ||||||||||||||||||||||||||||||||||| 71/78 | 2.2E-03 | 124 | light respnose |
| LTS | McCormac_2-4_gun-mutant-Dpretreated_Rep1_ATH1 | |||||||||||||||||||||||||||||||| 64/85 | 2.1E-02 | 89 | light response |
| STS | Broadley_1-2_A2-Bo-P-phosphate-starved_Rep1_ATH1 | |||||||||||||||||||||||||||||| 61/182 | 4.4E-10 | 121*** | nutrient_phosphate |
| LTS | Schroeder_1-6_JS43-control-96h_Rep1_ATH1 | |||||||||||||||||||||||||||||||| 64/85 | 2.1E-02 | 354 | nutrient_potassium |
| STS | Weise_1-2_Col-WT_Rep2_ATH1 | ||||||||||||||||||| 39/204 | 9.5E-05 | 411 | nutrient_starch |
| LTS | Torres_2-37_W0.1_Rep1_ATH1 | |||||||||||||||||||||||||||||| 61/88 | 4.5E-02 | 345 | pathogen |
| LTS | Underwood_1-15_Cor-5x10e7-10h_Rep3_ATH1 | ||||||||||||||||||||||||||||||||| 66/83 | 1.1E-02 | 340 | pathogen |
| LTS | Ulker_2-4_WRKY-KO-30-Pst-DC3000_Rep1_ATH1 | |||||||||||||||||||||||||||||| 61/88 | 4.5E-02 | 398 | pathogen |
| LTS | Marco_2-28_Nd-DeltaPopP2-12H_Rep2_ATH1 | ||||||||||||||||||||||||||||||||| 67/82 | 8.4E-03 | 447 | pathogen |
| STS | Diamond_A-1-Diamo-met_SLD | |||||||||||||| 29/214 | 7.3E-03 | 77 | pathogen |
| STS | Garton_1-2_sfr3-cold_Rep1_ATH1 | |||||||||||| 24/219 | 4.3E-02 | 383 | stress_cold |
| LTS | Hammond_3-11_Potassium-starved-root_Rep2_ATH1 | |||||||||||||||||||||||||||||||| 64/85 | 2.1E-02 | 105 | stress_Cs |
| LTS | AtGen_6-9821_Heatstress(3h)+1hrecovery-Roots-4.0h_Rep1 | |||||||||||||||||||||||||||||||| 65/84 | 1.5E-02 | 146 | stress_heat response |
| LTS | Scrase-Field_1-4_camta1-2-Col-0_Rep2_ATH1 | ||||||||||||||||||||||||||||||| 62/87 | 3.5E-02 | 127 | stress_knock out |
| LTS | AtGen_6-2422_Osmoticstress-Roots-6.0h_Rep2 | ||||||||||||||||||||||||||||||||| 66/83 | 1.1E-02 | 139 | stress_osmotic response |
| LTS | AtGen_6-6621_Oxidativestress-Roots-24.0h_Rep1 | ||||||||||||||||||||||||||||||| 63/86 | 2.7E-02 | 143 | stress_oxidative response |
| LTS | AtGen_6-8423_Woundingstress-Roots-6.0h_Rep1 | ||||||||||||||||||||||||||||||| 62/87 | 3.5E-02 | 145 | stress_wounding response |
| STS | Pieterse_2-5_ISR-0h_Rep1_ATH1 | |||||||||||||||||||||||| 48/195 | 9.2E-07 | 463 | stress_wounding response |

**p*-value is calculated by comparing the expressed/non-expressed gene numbers in each dataset with the average expressed/non-expressed gene numbers of the corresponding group using Fisher's exact test. The numerator shows the expressed gene number and the denominator shows the non-expressed gene number in each slide. The bar codes display the numbers of expressed genes. **The title of each experiment is attached below. ***The three experiments showed one dataset with a higher enrichment of expressed STS genes than that of expressed LTS genes (see Table 3)

**Exp_ID Titl of experiments**

6 Baseline_experiment_pollen Caution! This experiment has not been fully annotated, although all data is available

14 Control of lignification

23 Analysis of anther development by identifying downstream genes controlled by MS1

27 Assembly of the cell wall pectic matrix

30 Genes Involved in Plant Programmed Cell Death (PCD)

45 Identification of genes differentially expressed in dormant versus active axillary shoot apical mersitems.

48 Transcriptome analysis of Arabidopsis microgametogenesis

54 Role of COV in vascular patterning

55 Transcriptional profiling of laser-capture micro-dissected embryonic tissues

61 Carrera Bergua: Functional genomics of shoot meristem dormancy.

69 A genomic approach to understanding seed dormancy

77 Identification of Core Genes Regulating Plant Programmed Cell Death (PCD)

80 The trans-differentiation of cultured Arabidopsis cells

85 Molecular bases of zinc tolerance and accumulation by Arabidopsis halleri

89 Seedling transcriptome affected by a far-red light preconditioning treatment to block chloroplast development.

92 The mechanisms involved in the interplay between dormancy and secondary growth in Arabidopsis

103 Identification of genes involved in nutritional regulation of root architecture

104 Global gene expression in two species of Brassicaceae: Thlaspi caerulescens and Thlaspi arvense (cross-species study)

105 Differential gene expression patterns in potassium-starved and Caesium-treated plants

117 Identification of genes involved in secondary cell wall development in the hypocotyls of short day grown Arabidopsis

121 Changes in Gene Expression in Brassica oleracea Shoots during Phosphate Starvation (Cross-species study)

124 AtGenExpress: Light treatments

127 Comparative transcriptome analysis of wild type and two knockout alleles of At-CAMTA

139 AtGenExpress: Stress Treatments (Osmotic stress)

143 AtGenExpress: Stress Treatments (Oxidative stress)

145 AtGenExpress: Stress Treatments (Wounding stress)

146 AtGenExpress: Stress Treatments (Heat stress)

150 AtGenExpress: Developmental series (leaves)

151 AtGenExpress: Developmental series (roots)

153 AtGenExpress: Developmental series (shoots and stems)

183 AtGenExpress: Effect of ABA during seed imbibition

195 AtGenExpress: Expression profiling of early germinating seeds

196 Circadian gene expression under different light treatments

339 Using transcriptome analysis to define the developmental state of fn10, a mutant in Arabidopsis thaliana with an increased level of dormancy.

340 Genome-wide transcriptional analysis of the compatible Arabidopsis thaliana-Pseudomonas syringae pv. tomato DC3000 interaction

341 Nuclear gene expression in response to chloroplast division inhibition

345 AvrPtoB effector function

354 Response to potassium starvation in roots

360 Genome-wide cell cycle studies

361 In vitro tracheary element transdifferentiation of Col-0 suspension cells.

381 Growth of suspension-cultured cells

383 Cold acclimation in the sfr3-1 mutant

384 Cesium Toxicity in Arabidopsis

398 Group II-A WRKY transcription factors and early leaf senescence

404 Cold Acclimation Time Course

411 Transcript inhibition from limited starch degradation

412 Gametophytic transcription factors

419 Signaling from an Altered Cell Wall to the Nucleus Mediates Sugar-responsive Growth and Development in A. thaliana

420 prr mutants and 35S::PRR transgenics under constant light conditions

426 Photoreceptor control of shoot meristem activity and leaf initiation

447 Interaction Arabidopsis thaliana vs. Ralstonia solanacearum

463 Priming for enhanced MeJA-responsive gene expression in Arabidopsis plants expressing P. fluorescens WCS417r-mediated ISR

477 Expression comparison for white light grown Wt vs knockout arabidopsis

490 Cell-specific nitrogen responses in the Arabidopsis root

497 Transcriptome analysis of cho1 mutant during and after seed germination

498 Transcriptome analysis during axillary shoot outgrowth

499 Transcriptome analysis of Col and Cvi accession seeds immediately after imbibition

505 GA-dependant SPT targets
