# Supplementary figures and images for: Phylogenetic Comparison of F-Box (FBX) Gene Superfamily within the Plant Kingdom Reveals Divergent Evolutionary Histories Indicative of Genomic Drift
Source: PLoS One. 2011 Jan 28;6(1):e16219. doi: 10.1371/journal.pone.0016219 (PMC3030570; doi:10.1371/journal.pone.0016219)

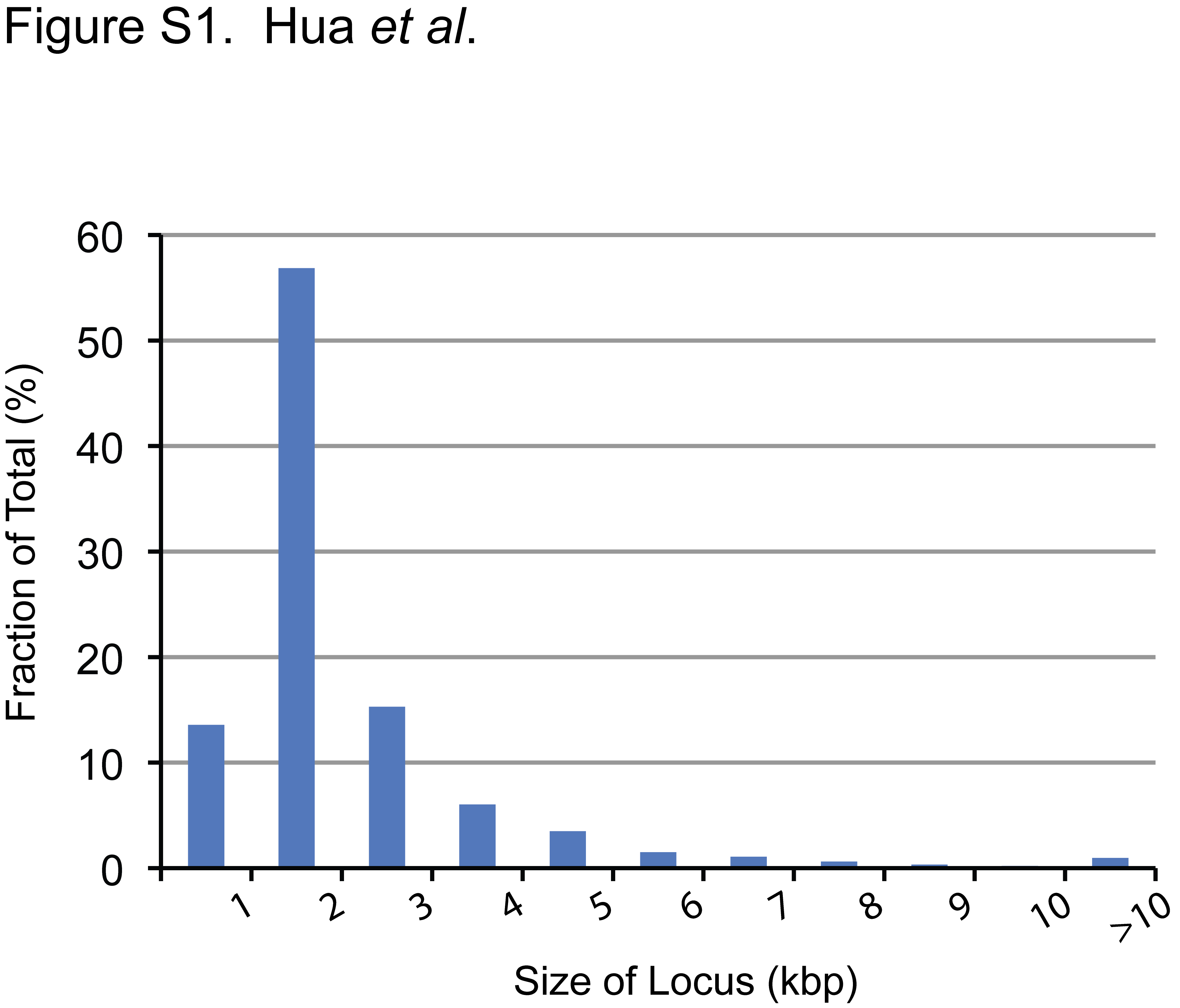

Supplement: Figure S1 — Size distributions in nucleotides of the complete set of FBX genes predicted from all 18 plant species (see Table S1 for list and abbreviation for each species). (TIF) [file pone.0016219.s011.tif]

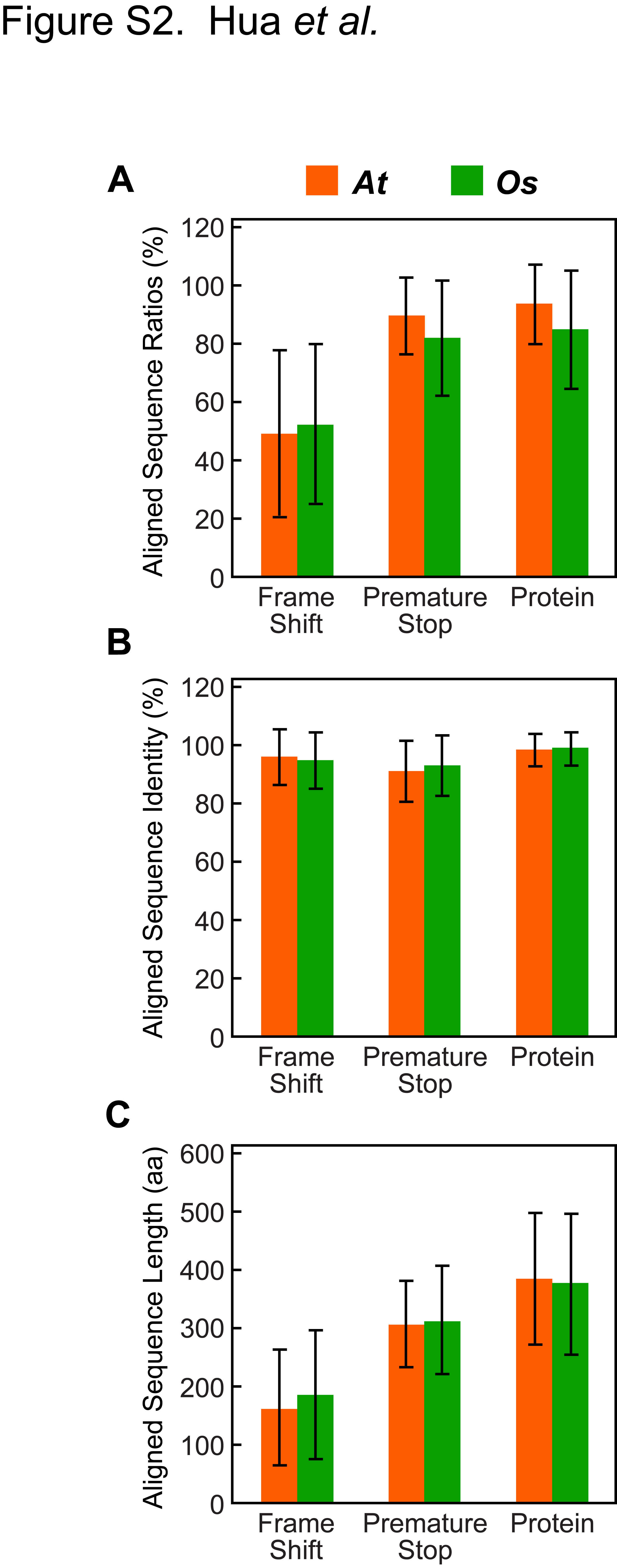

Supplement: Figure S2 — Evaluations of the CTT and the similarity-based FBX gene annotation protocols. A. thaliana (At). O. sativa (Os). “Frame shift” indicates FBX genes containing one or more reading-frame shifts. “Premature Stop” indicates FBX genes containing premature stop codon(s). “Protein” indicates FBX protein-coding genes. (TIF) [file pone.0016219.s012.tif]

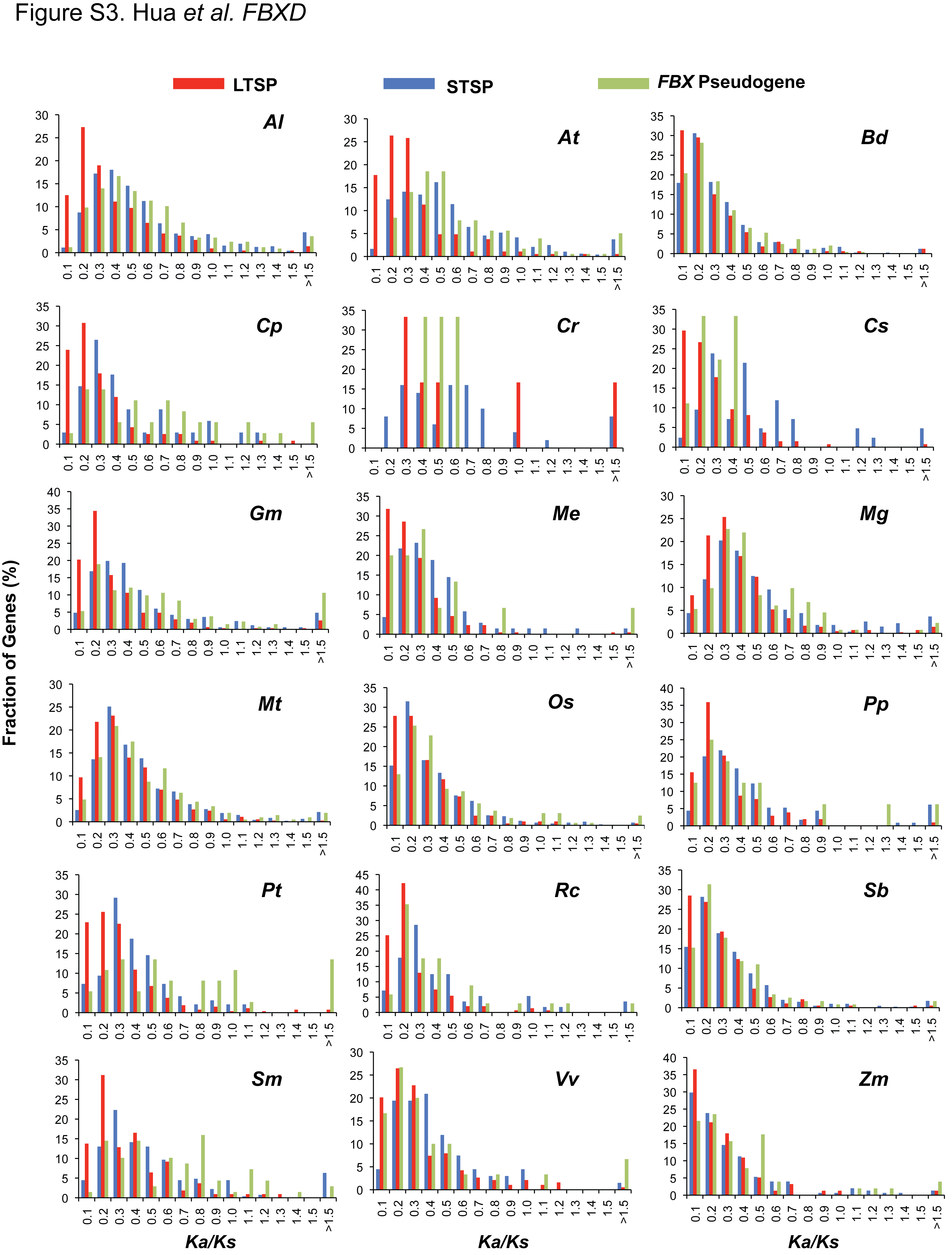

Supplement: Figure S3 — The distributions of FBXD Ka/Ks values of FBX genes from the LTSP, STSP and FBX pseudogene groups in each of 18 plant species. The Ka/Ks value for each FBXD sequence was calculated by comparing FBXD sequence to the MRCA transcript sequence using the method of Goldman and Yang [34]. (TIF) [file pone.0016219.s013.tif]

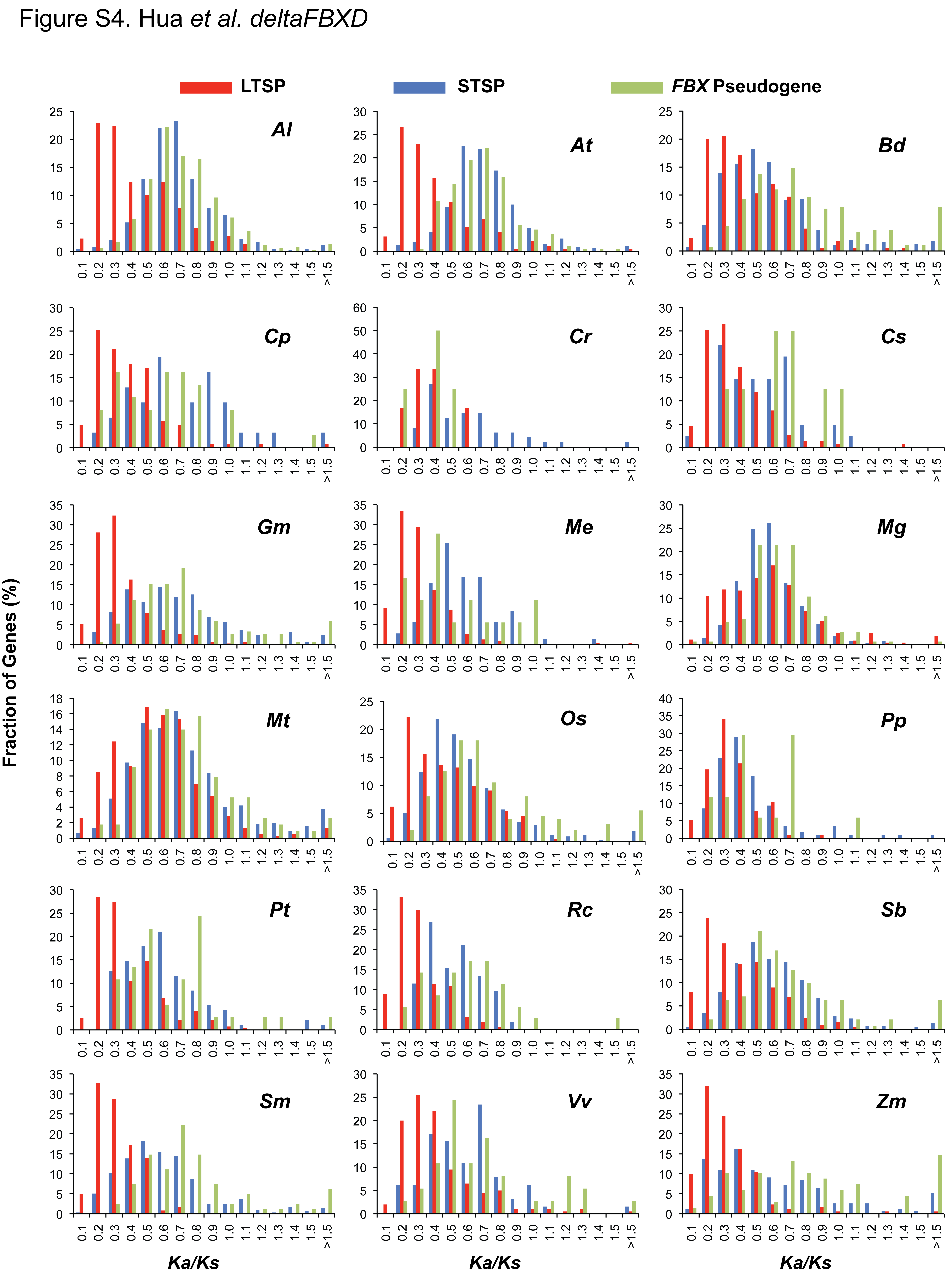

Supplement: Figure S4 — The distributions of Δ FBXD Ka/Ks values of FBX genes from the LTSP, STSP and FBX pseudogene groups in each of 18 plant species. The Ka/Ks value for each ΔFBXD sequence was calculated by comparing ΔFBXD sequence to the MRCA transcript sequence using the method of Goldman and Yang [34]. (TIF) [file pone.0016219.s014.tif]

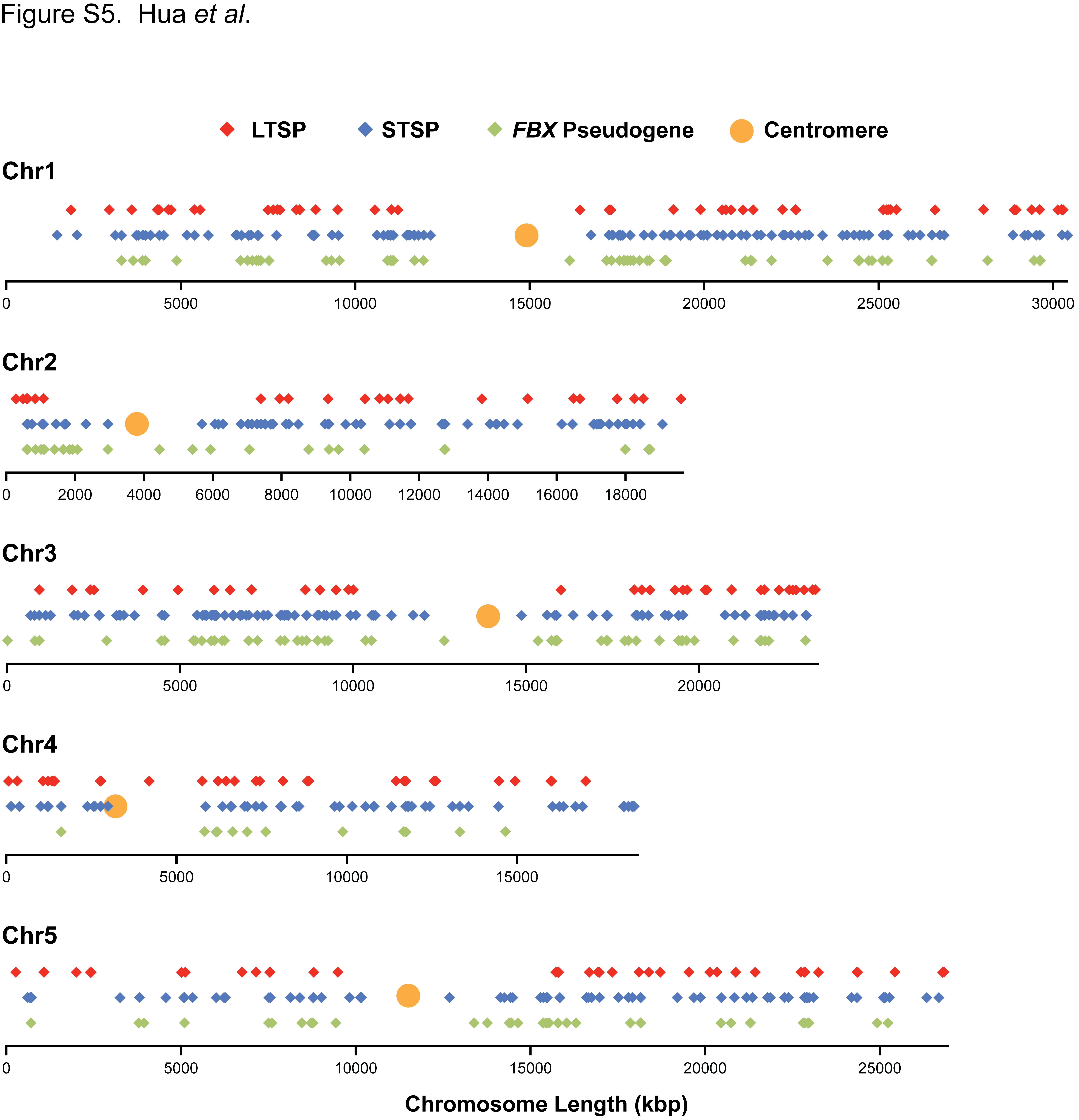

Supplement: Figure S5 — The distributions of LTSP, STSP and FBX pseudogene loci along the chromosomes of A. thaliana . (TIF) [file pone.0016219.s015.tif]

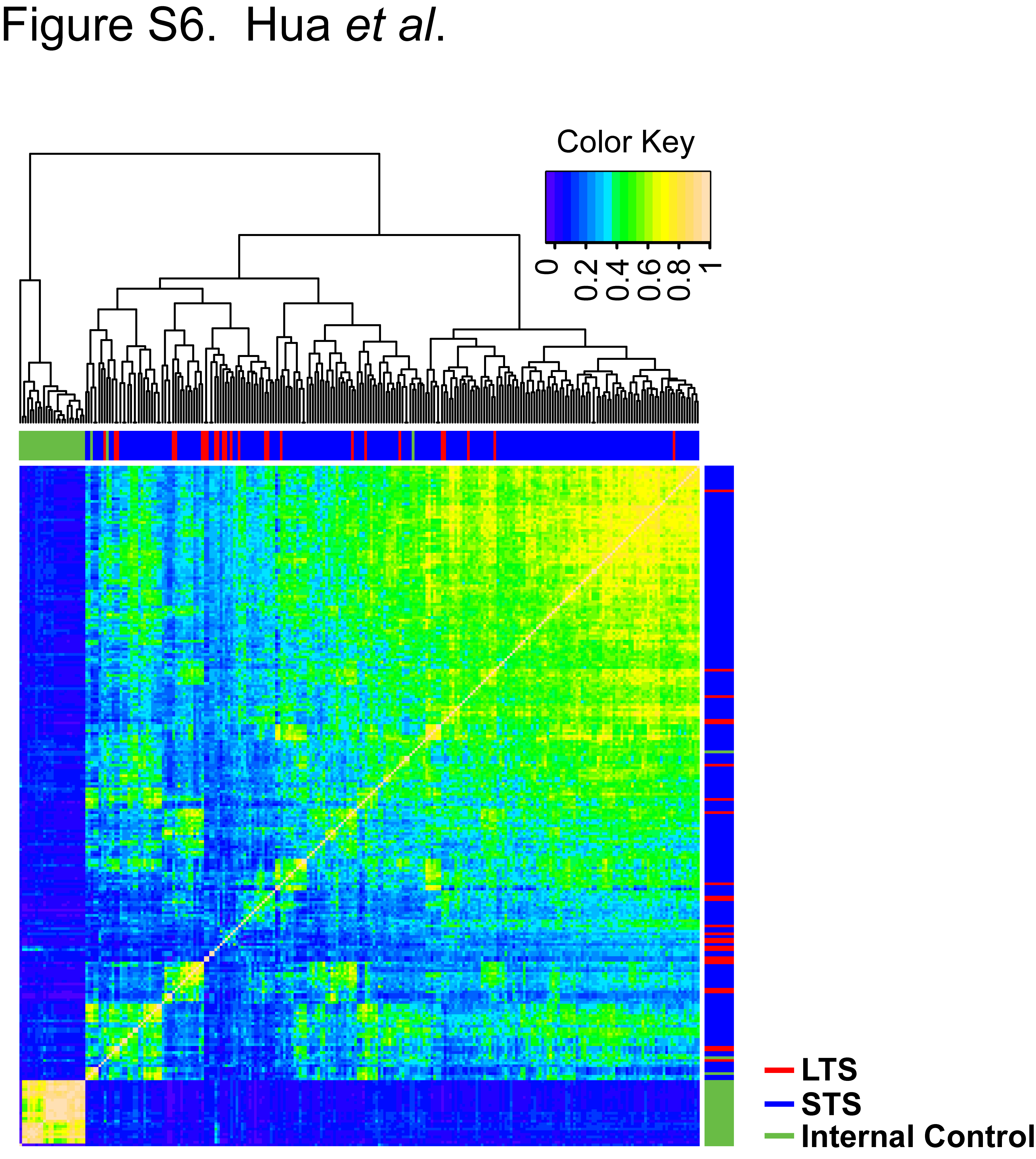

Supplement: Figure S6 — Microarray data correlations of A. thaliana FBX genes from cluster b in Figure 8A , and 28 internal non-plant genes from NASCArrays. The Pearson's correlation coefficients were calculated as described in Figure 8. The dendrogram on the top of the panel showed the hierarchical clustering based on the dissimilarities of Pearson's correlation coefficients. The bar codes indicate the distributions of LTS FBX genes (red color), STS FBX genes (blue color), and the 28 non-plant internal control genes (green color). (TIF) [file pone.0016219.s016.tif]
