# Supplementary material for: Phylogenetic Comparison of F-Box (FBX) Gene Superfamily within the Plant Kingdom Reveals Divergent Evolutionary Histories Indicative of Genomic Drift
Source: PLoS One. 2011 Jan 28;6(1):e16219. doi: 10.1371/journal.pone.0016219 (PMC3030570; doi:10.1371/journal.pone.0016219)
Supplement: Supplementary file 11 [file pone.0016219.s007.doc]

## Sheet: Sheet1

Table S7.  Fisher's exact test of the enrichment or depletion of domain (dmn) combinations in the putative substrate recruitment module 
of FBX proteins in each plant species.   
Species	#FBX in the species	dmn	#FBX w/ dmn in the species	#FBX w/dmn in all 18 species	#FBX w/o dmn in the species	#FBX w/o dmn in all 18 species	p (left)	p (right)	%
Al**	1350	No Prediction	243	3589	1107	7222	1	< 2.2e-16	18
At**	897	No Prediction	172	3589	725	7222	1	< 2.2e-16	19.175027870680044
Bd*	998	No Prediction	492	3589	506	7222	< 2.2e-16	1	49.298597194388776
Cp	198	No Prediction	66	3589	132	7222	0.5111	0.5495	33.33333333333333
Cr*	88	No Prediction	44	3589	44	7222	0.000861	0.9996	50
Cs	207	No Prediction	61	3589	146	7222	0.8864	0.1456	29.468599033816425
Gm*	702	No Prediction	261	3589	441	7222	0.01731	0.9859	37.17948717948718
Me	323	No Prediction	111	3589	212	7222	0.3503	0.6927	34.36532507739938
Mg**	903	No Prediction	249	3589	654	7222	0.9998	0.0002655	27.57475083056478
Mt**	1148	No Prediction	334	3589	814	7222	0.998	0.002515	29.094076655052266
Os*	971	No Prediction	365	3589	606	7222	0.003247	0.9974	37.59011328527291
Pp	258	No Prediction	89	3589	169	7222	0.3532	0.6949	34.49612403100775
Pt	425	No Prediction	140	3589	285	7222	0.5625	0.4792	32.94117647058823
Rc	250	No Prediction	74	3589	176	7222	0.8975	0.1294	29.599999999999998
Sb*	817	No Prediction	357	3589	460	7222	0.000000001184	1	43.696450428396574
Sm*	544	No Prediction	247	3589	297	7222	0.000000005636	1	45.404411764705884
Vv	315	No Prediction	109	3589	206	7222	0.3205	0.7213	34.6031746031746
Zm*	417	No Prediction	175	3589	242	7222	0.0001513	0.9999	41.96642685851319
Al*	1350	Rare	522	3878	828	6933	0.02387	0.9793	38.666666666666664
At*	897	Rare	347	3878	550	6933	0.04995	0.957	38.684503901895205
Bd**	998	Rare	290	3878	708	6933	1	0.000007432	29.058116232464933
Cp	198	Rare	67	3878	131	6933	0.746	0.3046	33.83838383838384
Cr	88	Rare	39	3878	49	6933	0.06402	0.959	44.31818181818182
Cs	207	Rare	85	3878	122	6933	0.07187	0.9458	41.06280193236715
Gm**	702	Rare	265	3878	437	6933	0.1674	0.852	37.749287749287745
Me**	323	Rare	92	3878	231	6933	0.9977	0.003354	28.482972136222912
Mg*	903	Rare	350	3878	553	6933	0.045	0.9614	38.759689922480625
Mt*	1148	Rare	558	3878	590	6933	< 2.2e-16	1	48.606271777003485
Os**	971	Rare	290	3878	681	6933	1	0.00008666	29.866117404737384
Pp	258	Rare	92	3878	166	6933	0.5517	0.5006	35.65891472868217
Pt	425	Rare	154	3878	271	6933	0.4576	0.583	36.23529411764706
Rc	250	Rare	84	3878	166	6933	0.7896	0.2514	33.6
Sb**	817	Rare	217	3878	600	6933	1	0.00000002636	26.560587515299876
Sm	544	Rare	196	3878	348	6933	0.4867	0.5497	36.029411764705884
Vv	315	Rare	118	3878	197	6933	0.3002	0.7396	37.46031746031746
Zm**	417	Rare	112	3878	305	6933	1	0.00007416	26.85851318944844
Al*	1350	FBA_CLAN	226	855	1124	9956	< 2.2e-16	1	16.74074074074074
At*	897	FBA_CLAN	139	855	758	9956	0.000000000000525	1	15.496098104793758
Bd**	998	FBA_CLAN	39	855	959	9956	1	0.0000005359	3.9078156312625247
Cp	198	FBA_CLAN	11	855	187	9956	0.9177	0.1368	5.555555555555555
Cr**	88	FBA_CLAN	0	855	88	9956	1	0.0007323	0
Cs	207	FBA_CLAN	10	855	197	9956	0.9683	0.05985	4.830917874396135
Gm	702	FBA_CLAN	41	855	661	9956	0.983	0.02478	5.84045584045584
Me	323	FBA_CLAN	27	855	296	9956	0.4139	0.6639	8.359133126934983
Mg*	903	FBA_CLAN	102	855	801	9956	0.0003867	0.9997	11.295681063122924
Mt	1148	FBA_CLAN	84	855	1064	9956	0.7766	0.2601	7.317073170731707
Os**	971	FBA_CLAN	50	855	921	9956	0.9995	0.0007528	5.149330587023687
Pp**	258	FBA_CLAN	4	855	254	9956	1	0.000008262	1.550387596899225
Pt	425	FBA_CLAN	33	855	392	9956	0.57	0.5031	7.764705882352942
Rc	250	FBA_CLAN	22	855	228	9956	0.3358	0.7431	8.799999999999999
Sb**	817	FBA_CLAN	42	855	775	9956	0.999	0.001728	5.140758873929008
Sm**	544	FBA_CLAN	1	855	543	9956	1	< 2.2e-16	0.1838235294117647
Vv**	315	FBA_CLAN	11	855	304	9956	0.9995	0.0012	3.492063492063492
Zm**	417	FBA_CLAN	13	855	404	9956	1	0.00005104	3.117505995203837
Al*	1350	kelch_CLAN	89	578	1261	10233	0.03582	0.9727	6.592592592592593
At	897	kelch_CLAN	59	578	838	10233	0.07149	0.9463	6.577480490523968
Bd**	998	kelch_CLAN	18	578	980	10233	1	0.00000002919	1.8036072144288577
Cp	198	kelch_CLAN	13	578	185	10233	0.2661	0.822	6.565656565656567
Cr**	88	kelch_CLAN	0	578	88	10233	1	0.008108	0
Cs	207	kelch_CLAN	16	578	191	10233	0.09333	0.945	7.729468599033816
Gm	702	kelch_CLAN	44	578	658	10233	0.1679	0.8702	6.267806267806268
Me*	323	kelch_CLAN	26	578	297	10233	0.02841	0.9832	8.04953560371517
Mg**	903	kelch_CLAN	27	578	876	10233	0.9996	0.0007057	2.990033222591362
Mt**	1148	kelch_CLAN	25	578	1123	10233	1	0.0000001997	2.177700348432056
Os**	971	kelch_CLAN	29	578	942	10233	0.9998	0.0004574	2.9866117404737382
Pp*	258	kelch_CLAN	47	578	211	10233	0.000000000000545	1	18.217054263565892
Pt	425	kelch_CLAN	28	578	397	10233	0.1578	0.8867	6.588235294117648
Rc	250	kelch_CLAN	19	578	231	10233	0.083	0.9494	7.6
Sb**	817	kelch_CLAN	26	578	791	10233	0.9984	0.002936	3.182374541003672
Sm*	544	kelch_CLAN	63	578	481	10233	0.00000003144	1	11.580882352941178
Vv	315	kelch_CLAN	20	578	295	10233	0.2508	0.8192	6.349206349206349
Zm	417	kelch_CLAN	29	578	388	10233	0.09744	0.9333	6.954436450839328
Al	1350	DUF295	41	370	1309	10441	0.7917	0.2585	3.037037037037037
At	897	DUF295	28	370	869	10441	0.7111	0.3594	3.121516164994426
Bd	998	DUF295	39	370	959	10441	0.2347	0.8154	3.9078156312625247
Cp	198	DUF295	7	370	191	10441	0.5202	0.6323	3.535353535353535
Cr**	88	DUF295	0	370	88	10441	1	0.04728	0
Cs	207	DUF295	5	370	202	10441	0.8383	0.2881	2.4154589371980677
Gm**	702	DUF295	3	370	699	10441	1	0.0000001267	0.4273504273504274
Me	323	DUF295	7	370	316	10441	0.9253	0.1391	2.1671826625387
Mg**	903	DUF295	9	370	894	10441	1	0.000005366	0.9966777408637874
Mt**	1148	DUF295	13	370	1135	10441	1	0.000001967	1.132404181184669
Os*	971	DUF295	97	370	874	10441	< 2.2e-16	1	9.989701338825952
Pp**	258	DUF295	0	370	258	10441	1	0.0001396	0
Pt**	425	DUF295	7	370	418	10441	0.99	0.02355	1.647058823529412
Rc	250	DUF295	11	370	239	10441	0.2444	0.8452	4.3999999999999995
Sb*	817	DUF295	54	370	763	10441	0.00001329	1	6.609547123623011
Sm**	544	DUF295	0	370	544	10441	1	0.000000009479	0
Vv	315	DUF295	15	370	300	10441	0.1317	0.9193	4.761904761904762
Zm*	417	DUF295	34	370	383	10441	0.00000691	1	8.15347721822542
Al*	1350	LRR_CLAN+FBD	112	312	1238	10499	< 2.2e-16	1	8.296296296296296
At*	897	LRR_CLAN+FBD	76	312	821	10499	0.00000000000001135	1	8.4726867335563
Bd**	998	LRR_CLAN+FBD	16	312	982	10499	0.9957	0.008321	1.6032064128256511
Cp	198	LRR_CLAN+FBD	5	312	193	10499	0.6789	0.492	2.525252525252525
Cr	88	LRR_CLAN+FBD	0	312	88	10499	1	0.07682	0
Cs**	207	LRR_CLAN+FBD	1	312	206	10499	0.9976	0.01737	0.4830917874396135
Gm**	702	LRR_CLAN+FBD	5	312	697	10499	1	0.0000721	0.7122507122507122
Me**	323	LRR_CLAN+FBD	4	312	319	10499	0.9834	0.04509	1.238390092879257
Mg	903	LRR_CLAN+FBD	24	312	879	10499	0.6834	0.3954	2.6578073089700998
Mt	1148	LRR_CLAN+FBD	30	312	1118	10499	0.7276	0.3393	2.6132404181184667
Os**	971	LRR_CLAN+FBD	14	312	957	10499	0.9985	0.00333	1.4418125643666324
Pp**	258	LRR_CLAN+FBD	0	312	258	10499	1	0.0005728	0
Pt**	425	LRR_CLAN+FBD	4	312	421	10499	0.9981	0.006495	0.9411764705882352
Rc	250	LRR_CLAN+FBD	3	312	247	10499	0.9755	0.07065	1.2
Sb	817	LRR_CLAN+FBD	16	312	801	10499	0.957	0.07045	1.9583843329253363
Sm**	544	LRR_CLAN+FBD	0	312	544	10499	1	0.0000001789	0
Vv**	315	LRR_CLAN+FBD	1	312	314	10499	0.9999	0.001137	0.31746031746031744
Zm**	417	LRR_CLAN+FBD	1	312	416	10499	1	0.00008114	0.2398081534772182
Al**	1350	FBD	13	285	1337	10526	1	0.00002462	0.962962962962963
At**	897	FBD	10	285	887	10526	0.9993	0.001672	1.1148272017837235
Bd*	998	FBD	47	285	951	10526	0.0002986	0.9998	4.709418837675351
Cp	198	FBD	6	285	192	10526	0.4258	0.73	3.0303030303030303
Cr	88	FBD	0	285	88	10526	1	0.0962	0
Cs**	207	FBD	0	285	207	10526	1	0.004182	0
Gm**	702	FBD	6	285	696	10526	0.9997	0.0008622	0.8547008547008548
Me	323	FBD	7	285	316	10526	0.7481	0.384	2.1671826625387
Mg	903	FBD	21	285	882	10526	0.7436	0.3336	2.3255813953488373
Mt	1148	FBD	27	285	1121	10526	0.7448	0.3236	2.35191637630662
Os*	971	FBD	67	285	904	10526	0.00000000005054	1	6.90010298661174
Pp**	258	FBD	0	285	258	10526	1	0.001102	0
Pt	425	FBD	8	285	417	10526	0.8703	0.2161	1.8823529411764703
Rc	250	FBD	6	285	244	10526	0.6476	0.5122	2.4
Sb*	817	FBD	54	285	763	10526	0.000000009525	1	6.609547123623011
Sm**	544	FBD	0	285	544	10526	1	0.0000006985	0
Vv**	315	FBD	1	285	314	10526	0.9998	0.002322	0.31746031746031744
Zm	417	FBD	12	285	405	10526	0.4244	0.689	2.877697841726619
Al*	1350	LRR_CLAN	54	253	1296	10558	0.0003898	0.9998	4
At	897	LRR_CLAN	22	253	875	10558	0.4485	0.639	2.4526198439241917
Bd**	998	LRR_CLAN	14	253	984	10558	0.9835	0.03035	1.402805611222445
Cp	198	LRR_CLAN	3	253	195	10558	0.8435	0.3196	1.5151515151515151
Cr	88	LRR_CLAN	1	253	87	10558	0.8755	0.388	1.1363636363636365
Cs	207	LRR_CLAN	3	253	204	10558	0.864	0.287	1.4492753623188406
Gm**	702	LRR_CLAN	6	253	696	10558	0.9989	0.003464	0.8547008547008548
Me	323	LRR_CLAN	3	253	320	10558	0.9808	0.05743	0.9287925696594427
Mg*	903	LRR_CLAN	60	253	843	10558	0.00000000002671	1	6.64451827242525
Mt	1148	LRR_CLAN	27	253	1121	10558	0.5205	0.561	2.35191637630662
Os	971	LRR_CLAN	19	253	952	10558	0.8071	0.2633	1.956745623069001
Pp	258	LRR_CLAN	3	253	255	10558	0.9407	0.1479	1.1627906976744187
Pt	425	LRR_CLAN	8	253	417	10558	0.7764	0.3407	1.8823529411764703
Rc**	250	LRR_CLAN	1	253	249	10558	0.9972	0.0197	0.4
Sb	817	LRR_CLAN	13	253	804	10558	0.9399	0.0996	1.591187270501836
Sm**	544	LRR_CLAN	2	253	542	10558	1	0.000316	0.3676470588235294
Vv	315	LRR_CLAN	12	253	303	10558	0.07402	0.9615	3.8095238095238098
Zm**	417	LRR_CLAN	2	253	415	10558	0.9994	0.003534	0.4796163069544364
Al**	1350	Tubby_c_CLAN+DUF3527	7	117	1343	10694	0.988	0.02815	0.5185185185185185
At	897	Tubby_c_CLAN+DUF3527	7	117	890	10694	0.847	0.2569	0.7803790412486065
Bd	998	Tubby_c_CLAN+DUF3527	9	117	989	10694	0.748	0.3704	0.9018036072144289
Cp	198	Tubby_c_CLAN+DUF3527	4	117	194	10694	0.1736	0.9329	2.0202020202020203
Cr	88	Tubby_c_CLAN+DUF3527	0	117	88	10694	1	0.3853	0
Cs	207	Tubby_c_CLAN+DUF3527	4	117	203	10694	0.193	0.9223	1.932367149758454
Gm*	702	Tubby_c_CLAN+DUF3527	14	117	688	10694	0.02854	0.9865	1.9943019943019942
Me*	323	Tubby_c_CLAN+DUF3527	13	117	310	10694	0.0001002	1	4.024767801857585
Mg**	903	Tubby_c_CLAN+DUF3527	3	117	900	10694	0.9961	0.01445	0.33222591362126247
Mt	1148	Tubby_c_CLAN+DUF3527	7	117	1141	10694	0.96	0.08182	0.6097560975609756
Os	971	Tubby_c_CLAN+DUF3527	10	117	961	10694	0.6074	0.5221	1.0298661174047374
Pp	258	Tubby_c_CLAN+DUF3527	1	117	257	10694	0.939	0.2344	0.3875968992248062
Pt*	425	Tubby_c_CLAN+DUF3527	9	117	416	10694	0.0494	0.979	2.1176470588235294
Rc	250	Tubby_c_CLAN+DUF3527	4	117	246	10694	0.2926	0.861	1.6
Sb	817	Tubby_c_CLAN+DUF3527	9	117	808	10694	0.5296	0.6077	1.101591187270502
Sm	544	Tubby_c_CLAN+DUF3527	6	117	538	10694	0.5413	0.6241	1.1029411764705883
Vv	315	Tubby_c_CLAN+DUF3527	2	117	313	10694	0.855	0.3405	0.6349206349206349
Zm	417	Tubby_c_CLAN+DUF3527	8	117	409	10694	0.09363	0.957	1.9184652278177456
Al**	1350	PRANC	2	76	1348	10735	0.999	0.005834	0.14814814814814814
At**	897	PRANC	1	76	896	10735	0.9979	0.01573	0.11148272017837235
Bd	998	PRANC	4	76	994	10735	0.9152	0.1828	0.4008016032064128
Cp	198	PRANC	3	76	195	10735	0.1697	0.9462	1.5151515151515151
Cr	88	PRANC	1	76	87	10735	0.4655	0.8719	1.1363636363636365
Cs	207	PRANC	2	76	205	10735	0.4326	0.8192	0.966183574879227
Gm	702	PRANC	4	76	698	10735	0.727	0.4567	0.5698005698005698
Me	323	PRANC	2	76	321	10735	0.6659	0.6047	0.6191950464396285
Mg	903	PRANC	10	76	893	10735	0.1245	0.9344	1.1074197120708749
Mt	1148	PRANC	11	76	1137	10735	0.2106	0.8729	0.9581881533101044
Os**	971	PRANC	2	76	969	10735	0.9904	0.03888	0.20597322348094746
Pp*	258	PRANC	6	76	252	10735	0.01211	0.997	2.3255813953488373
Pt	425	PRANC	2	76	423	10735	0.8001	0.4295	0.4705882352941176
Rc*	250	PRANC	8	76	242	10735	0.0006033	0.9999	3.2
Sb	817	PRANC	2	76	815	10735	0.9768	0.08118	0.24479804161566704
Sm*	544	PRANC	10	76	534	10735	0.007859	0.9974	1.8382352941176472
Vv	315	PRANC	4	76	311	10735	0.1909	0.9239	1.2698412698412698
Zm	417	PRANC	2	76	415	10735	0.7917	0.442	0.4796163069544364
Al	1350	TPR_1_CLAN	3	61	1347	10750	0.9786	0.0647	0.2222222222222222
At	897	TPR_1_CLAN	4	61	893	10750	0.744	0.4367	0.4459308807134894
Bd	998	TPR_1_CLAN	5	61	993	10750	0.6658	0.5111	0.501002004008016
Cp	198	TPR_1_CLAN	2	61	196	10750	0.3136	0.8958	1.0101010101010102
Cr	88	TPR_1_CLAN	0	61	88	10750	1	0.609	0
Cs	207	TPR_1_CLAN	2	61	205	10750	0.3322	0.8852	0.966183574879227
Gm	702	TPR_1_CLAN	4	61	698	10750	0.5658	0.6363	0.5698005698005698
Me	323	TPR_1_CLAN	6	61	317	10750	0.01287	0.9968	1.8575851393188854
Mg	903	TPR_1_CLAN	5	61	898	10750	0.5828	0.6	0.5537098560354374
Mt**	1148	TPR_1_CLAN	1	61	1147	10750	0.9981	0.01436	0.08710801393728224
Os	971	TPR_1_CLAN	2	61	969	10750	0.9708	0.09876	0.20597322348094746
Pp	258	TPR_1_CLAN	4	61	254	10750	0.06475	0.9824	1.550387596899225
Pt	425	TPR_1_CLAN	4	61	421	10750	0.231	0.9011	0.9411764705882352
Rc	250	TPR_1_CLAN	3	61	247	10750	0.1759	0.9435	1.2
Sb	817	TPR_1_CLAN	2	61	815	10750	0.942	0.171	0.24479804161566704
Sm*	544	TPR_1_CLAN	9	61	535	10750	0.005969	0.9983	1.6544117647058825
Vv	315	TPR_1_CLAN	2	61	313	10750	0.5363	0.736	0.6349206349206349
Zm	417	TPR_1_CLAN	3	61	414	10750	0.4264	0.7864	0.7194244604316548
Al	1350	FBA_CLAN+kelch_CLAN	13	60	1337	10751	0.05695	0.9717	0.962962962962963
At	897	FBA_CLAN+kelch_CLAN	8	60	889	10751	0.1474	0.9261	0.8918617614269788
Bd	998	FBA_CLAN+kelch_CLAN	2	60	996	10751	0.972	0.09516	0.2004008016032064
Cp	198	FBA_CLAN+kelch_CLAN	0	60	198	10751	1	0.3356	0
Cr	88	FBA_CLAN+kelch_CLAN	0	60	88	10751	1	0.614	0
Cs*	207	FBA_CLAN+kelch_CLAN	6	60	201	10751	0.001455	0.9998	2.898550724637681
Gm	702	FBA_CLAN+kelch_CLAN	1	60	701	10751	0.9787	0.1062	0.14245014245014245
Me	323	FBA_CLAN+kelch_CLAN	1	60	322	10751	0.8348	0.4679	0.30959752321981426
Mg	903	FBA_CLAN+kelch_CLAN	4	60	899	10751	0.7374	0.4451	0.4429678848283499
Mt	1148	FBA_CLAN+kelch_CLAN	11	60	1137	10751	0.07487	0.9633	0.9581881533101044
Os	971	FBA_CLAN+kelch_CLAN	2	60	969	10751	0.9686	0.1048	0.20597322348094746
Pp	258	FBA_CLAN+kelch_CLAN	2	60	256	10751	0.4258	0.8245	0.7751937984496124
Pt	425	FBA_CLAN+kelch_CLAN	1	60	424	10751	0.9054	0.3226	0.2352941176470588
Rc	250	FBA_CLAN+kelch_CLAN	2	60	248	10751	0.4106	0.8353	0.8
Sb	817	FBA_CLAN+kelch_CLAN	3	60	814	10751	0.829	0.3452	0.36719706242350064
Sm	544	FBA_CLAN+kelch_CLAN	0	60	544	10751	1	0.05216	0
Vv	315	FBA_CLAN+kelch_CLAN	3	60	312	10751	0.264	0.8973	0.9523809523809524
Zm	417	FBA_CLAN+kelch_CLAN	1	60	416	10751	0.9012	0.3325	0.2398081534772182
Al	1350	Beta_propeller_CLAN	3	47	1347	10764	0.927	0.1791	0.2222222222222222
At	897	Beta_propeller_CLAN	1	47	896	10764	0.9784	0.1081	0.11148272017837235
Bd	998	Beta_propeller_CLAN	3	47	995	10764	0.8064	0.3802	0.30060120240480964
Cp	198	Beta_propeller_CLAN	2	47	196	10764	0.2202	0.9422	1.0101010101010102
Cr	88	Beta_propeller_CLAN	2	47	86	10764	0.05936	0.9928	2.272727272727273
Cs	207	Beta_propeller_CLAN	1	47	206	10764	0.5984	0.7723	0.4830917874396135
Gm	702	Beta_propeller_CLAN	2	47	700	10764	0.809	0.4185	0.2849002849002849
Me	323	Beta_propeller_CLAN	4	47	319	10764	0.05991	0.9843	1.238390092879257
Mg	903	Beta_propeller_CLAN	2	47	901	10764	0.9005	0.2604	0.22148394241417496
Mt	1148	Beta_propeller_CLAN	1	47	1147	10764	0.9922	0.0477	0.08710801393728224
Os	971	Beta_propeller_CLAN	2	47	969	10764	0.9206	0.2195	0.20597322348094746
Pp	258	Beta_propeller_CLAN	3	47	255	10764	0.1106	0.9713	1.1627906976744187
Pt*	425	Beta_propeller_CLAN	5	47	420	10764	0.0459	0.9868	1.1764705882352942
Rc	250	Beta_propeller_CLAN	2	47	248	10764	0.3042	0.9014	0.8
Sb	817	Beta_propeller_CLAN	5	47	812	10764	0.3011	0.8442	0.6119951040391677
Sm	544	Beta_propeller_CLAN	4	47	540	10764	0.2271	0.904	0.7352941176470588
Vv	315	Beta_propeller_CLAN	3	47	312	10764	0.1679	0.9475	0.9523809523809524
Zm	417	Beta_propeller_CLAN	2	47	415	10764	0.5481	0.7264	0.4796163069544364
Al	1350	kelch_CLAN+PAS	3	41	1347	10770	0.8806	0.2649	0.2222222222222222
At	897	kelch_CLAN+PAS	3	41	894	10770	0.6658	0.5614	0.33444816053511706
Bd	998	kelch_CLAN+PAS	3	41	995	10770	0.7309	0.4834	0.30060120240480964
Cp	198	kelch_CLAN+PAS	0	41	198	10770	1	0.4745	0
Cr	88	kelch_CLAN+PAS	0	41	88	10770	1	0.7168	0
Cs	207	kelch_CLAN+PAS	2	41	205	10770	0.1932	0.9534	0.966183574879227
Gm	702	kelch_CLAN+PAS	6	41	696	10770	0.06443	0.9773	0.8547008547008548
Me*	323	kelch_CLAN+PAS	4	41	319	10770	0.0408	0.9906	1.238390092879257
Mg	903	kelch_CLAN+PAS	3	41	900	10770	0.67	0.5566	0.33222591362126247
Mt	1148	kelch_CLAN+PAS	1	41	1147	10770	0.9857	0.07841	0.08710801393728224
Os	971	kelch_CLAN+PAS	3	41	968	10770	0.7146	0.5037	0.30895983522142123
Pp	258	kelch_CLAN+PAS	0	41	258	10770	1	0.3796	0
Pt	425	kelch_CLAN+PAS	2	41	423	10770	0.4878	0.7786	0.4705882352941176
Rc	250	kelch_CLAN+PAS	2	41	248	10770	0.2537	0.9273	0.8
Sb	817	kelch_CLAN+PAS	3	41	814	10770	0.6061	0.6261	0.36719706242350064
Sm	544	kelch_CLAN+PAS	0	41	544	10770	1	0.1331	0
Vv	315	kelch_CLAN+PAS	3	41	312	10770	0.1278	0.9648	0.9523809523809524
Zm	417	kelch_CLAN+PAS	3	41	414	10770	0.2234	0.9205	0.7194244604316548
Al	1350	TPR_1_CLAN+zf-MYND	1	28	1349	10783	0.9672	0.152	0.07407407407407407
At	897	TPR_1_CLAN+zf-MYND	1	28	896	10783	0.9012	0.3372	0.11148272017837235
Bd	998	TPR_1_CLAN+zf-MYND	1	28	997	10783	0.923	0.2837	0.1002004008016032
Cp	198	TPR_1_CLAN+zf-MYND	1	28	197	10783	0.4096	0.9048	0.5050505050505051
Cr	88	TPR_1_CLAN+zf-MYND	0	28	88	10783	1	0.7967	0
Cs*	207	TPR_1_CLAN+zf-MYND	3	28	204	10783	0.01996	0.9974	1.4492753623188406
Gm*	702	TPR_1_CLAN+zf-MYND	6	28	696	10783	0.01563	0.9962	0.8547008547008548
Me	323	TPR_1_CLAN+zf-MYND	1	28	322	10783	0.5746	0.7949	0.30959752321981426
Mg	903	TPR_1_CLAN+zf-MYND	1	28	902	10783	0.9026	0.3338	0.11074197120708748
Mt	1148	TPR_1_CLAN+zf-MYND	1	28	1147	10783	0.9466	0.2182	0.08710801393728224
Os	971	TPR_1_CLAN+zf-MYND	2	28	969	10783	0.7205	0.5456	0.20597322348094746
Pp	258	TPR_1_CLAN+zf-MYND	0	28	258	10783	1	0.5162	0
Pt	425	TPR_1_CLAN+zf-MYND	2	28	423	10783	0.3146	0.897	0.4705882352941176
Rc	250	TPR_1_CLAN+zf-MYND	1	28	249	10783	0.4851	0.861	0.4
Sb	817	TPR_1_CLAN+zf-MYND	2	28	815	10783	0.6331	0.6465	0.24479804161566704
Sm	544	TPR_1_CLAN+zf-MYND	2	28	542	10783	0.4248	0.8281	0.3676470588235294
Vv	315	TPR_1_CLAN+zf-MYND	1	28	314	10783	0.5657	0.8023	0.31746031746031744
Zm	417	TPR_1_CLAN+zf-MYND	2	28	415	10783	0.307	0.9013	0.4796163069544364
Al	1350	FBA_CLAN+DUF1618	2	20	1348	10791	0.7188	0.552	0.14814814814814814
At	897	FBA_CLAN+DUF1618	3	20	894	10791	0.2564	0.9054	0.33444816053511706
Bd	998	FBA_CLAN+DUF1618	0	20	998	10791	1	0.1708	0
Cp	198	FBA_CLAN+DUF1618	0	20	198	10791	1	0.6954	0
Cr	88	FBA_CLAN+DUF1618	0	20	88	10791	1	0.8502	0
Cs	207	FBA_CLAN+DUF1618	0	20	207	10791	1	0.6841	0
Gm	702	FBA_CLAN+DUF1618	1	20	701	10791	0.7335	0.6306	0.14245014245014245
Me	323	FBA_CLAN+DUF1618	0	20	323	10791	1	0.5547	0
Mg	903	FBA_CLAN+DUF1618	1	20	902	10791	0.8148	0.5107	0.11074197120708748
Mt	1148	FBA_CLAN+DUF1618	0	20	1148	10791	1	0.1326	0
Os	971	FBA_CLAN+DUF1618	4	20	967	10791	0.1306	0.9571	0.4119464469618949
Pp	258	FBA_CLAN+DUF1618	0	20	258	10791	1	0.6237	0
Pt	425	FBA_CLAN+DUF1618	1	20	424	10791	0.5554	0.8124	0.2352941176470588
Rc	250	FBA_CLAN+DUF1618	0	20	250	10791	1	0.6328	0
Sb	817	FBA_CLAN+DUF1618	4	20	813	10791	0.08372	0.9765	0.4895960832313341
Sm	544	FBA_CLAN+DUF1618	0	20	544	10791	1	0.3743	0
Vv	315	FBA_CLAN+DUF1618	1	20	314	10791	0.4532	0.882	0.31746031746031744
Zm	417	FBA_CLAN+DUF1618	3	20	414	10791	0.05194	0.9905	0.7194244604316548
Al	1350	LysM	1	18	1349	10793	0.8933	0.3604	0.07407407407407407
At	897	LysM	1	18	896	10793	0.7803	0.5665	0.11148272017837235
Bd	998	LysM	0	18	998	10793	1	0.2038	0
Cp	198	LysM	1	18	197	10793	0.2919	0.955	0.5050505050505051
Cr	88	LysM	0	18	88	10793	1	0.8641	0
Cs	207	LysM	1	18	206	10793	0.3028	0.9513	0.4830917874396135
Gm	702	LysM	2	18	700	10793	0.3469	0.881	0.2849002849002849
Me	323	LysM	1	18	322	10793	0.4287	0.8962	0.30959752321981426
Mg	903	LysM	2	18	901	10793	0.4633	0.8034	0.22148394241417496
Mt	1148	LysM	0	18	1148	10793	1	0.1624	0
Os	971	LysM	1	18	970	10793	0.8051	0.5279	0.10298661174047373
Pp	258	LysM	1	18	257	10793	0.3614	0.9286	0.3875968992248062
Pt	425	LysM	2	18	423	10793	0.1738	0.962	0.4705882352941176
Rc	250	LysM	1	18	249	10793	0.3526	0.9324	0.4
Sb	817	LysM	1	18	816	10793	0.7498	0.6102	0.12239902080783352
Sm	544	LysM	1	18	543	10793	0.6068	0.7697	0.1838235294117647
Vv	315	LysM	1	18	314	10793	0.4208	0.9004	0.31746031746031744
Zm	417	LysM	1	18	416	10793	0.5131	0.8443	0.2398081534772182
Al	1350	AAA_CLAN	1	17	1349	10794	0.88	0.3904	0.07407407407407407
At	897	AAA_CLAN	1	17	896	10794	0.7621	0.5938	0.11148272017837235
Bd	998	AAA_CLAN	4	17	994	10794	0.09572	0.9723	0.4008016032064128
Cp	198	AAA_CLAN	0	17	198	10794	1	0.7344	0
Cr	88	AAA_CLAN	0	17	88	10794	1	0.8712	0
Cs	207	AAA_CLAN	0	17	207	10794	1	0.7242	0
Gm	702	AAA_CLAN	0	17	702	10794	1	0.3429	0
Me	323	AAA_CLAN	1	17	322	10794	0.4116	0.9054	0.30959752321981426
Mg	903	AAA_CLAN	0	17	903	10794	1	0.2555	0
Mt	1148	AAA_CLAN	2	17	1146	10794	0.5567	0.727	0.17421602787456447
Os	971	AAA_CLAN	1	17	970	10794	0.7876	0.5563	0.10298661174047373
Pp	258	AAA_CLAN	0	17	258	10794	1	0.6695	0
Pt	425	AAA_CLAN	0	17	425	10794	1	0.5189	0
Rc	250	AAA_CLAN	0	17	250	10794	1	0.6778	0
Sb	817	AAA_CLAN	0	17	817	10794	1	0.2896	0
Sm	544	AAA_CLAN	1	17	543	10794	0.587	0.7876	0.1838235294117647
Vv	315	AAA_CLAN	2	17	313	10794	0.09965	0.9844	0.6349206349206349
Zm*	417	AAA_CLAN	4	17	413	10794	0.006792	0.9991	0.9592326139088728
Al	1350	Elongin_A	0	17	1350	10794	1	0.1351	0
At	897	Elongin_A	1	17	896	10794	0.7621	0.5938	0.11148272017837235
Bd	998	Elongin_A	2	17	996	10794	0.4857	0.7868	0.2004008016032064
Cp	198	Elongin_A	0	17	198	10794	1	0.7344	0
Cr	88	Elongin_A	0	17	88	10794	1	0.8712	0
Cs	207	Elongin_A	0	17	207	10794	1	0.7242	0
Gm	702	Elongin_A	4	17	698	10794	0.03577	0.9925	0.5698005698005698
Me	323	Elongin_A	1	17	322	10794	0.4116	0.9054	0.30959752321981426
Mg	903	Elongin_A	3	17	900	10794	0.1966	0.937	0.33222591362126247
Mt	1148	Elongin_A	2	17	1146	10794	0.5567	0.727	0.17421602787456447
Os	971	Elongin_A	1	17	970	10794	0.7876	0.5563	0.10298661174047373
Pp	258	Elongin_A	0	17	258	10794	1	0.6695	0
Pt	425	Elongin_A	1	17	424	10794	0.5007	0.8531	0.2352941176470588
Rc	250	Elongin_A	1	17	249	10794	0.3376	0.9386	0.4
Sb	817	Elongin_A	0	17	817	10794	1	0.2896	0
Sm	544	Elongin_A	0	17	544	10794	1	0.4338	0
Vv	315	Elongin_A	0	17	315	10794	1	0.6135	0
Zm	417	Elongin_A	1	17	416	10794	0.4943	0.8574	0.2398081534772182
Al	1350	kelch_CLAN+Beta_propeller_CLAN	1	16	1349	10795	0.8649	0.4223	0.07407407407407407
At	897	kelch_CLAN+Beta_propeller_CLAN	1	16	896	10795	0.7423	0.6217	0.11148272017837235
Bd	998	kelch_CLAN+Beta_propeller_CLAN	0	16	998	10795	1	0.2432	0
Cp*	198	kelch_CLAN+Beta_propeller_CLAN	2	16	196	10795	0.04077	0.9962	1.0101010101010102
Cr	88	kelch_CLAN+Beta_propeller_CLAN	0	16	88	10795	1	0.8783	0
Cs	207	kelch_CLAN+Beta_propeller_CLAN	0	16	207	10795	1	0.7381	0
Gm	702	kelch_CLAN+Beta_propeller_CLAN	1	16	701	10795	0.6571	0.722	0.14245014245014245
Me*	323	kelch_CLAN+Beta_propeller_CLAN	3	16	320	10795	0.0166	0.998	0.9287925696594427
Mg	903	kelch_CLAN+Beta_propeller_CLAN	1	16	902	10795	0.7445	0.6187	0.11074197120708748
Mt	1148	kelch_CLAN+Beta_propeller_CLAN	0	16	1148	10795	1	0.1987	0
Os	971	kelch_CLAN+Beta_propeller_CLAN	0	16	971	10795	1	0.2523	0
Pp	258	kelch_CLAN+Beta_propeller_CLAN	1	16	257	10795	0.3305	0.9415	0.3875968992248062
Pt*	425	kelch_CLAN+Beta_propeller_CLAN	3	16	422	10795	0.03319	0.995	0.7058823529411765
Rc	250	kelch_CLAN+Beta_propeller_CLAN	1	16	249	10795	0.3222	0.9446	0.4
Sb	817	kelch_CLAN+Beta_propeller_CLAN	0	16	817	10795	1	0.3115	0
Sm	544	kelch_CLAN+Beta_propeller_CLAN	0	16	544	10795	1	0.4556	0
Vv	315	kelch_CLAN+Beta_propeller_CLAN	2	16	313	10795	0.0908	0.9866	0.6349206349206349
Zm	417	kelch_CLAN+Beta_propeller_CLAN	0	16	417	10795	1	0.5456	0
Al	1350	Cupin_clan	1	16	1349	10795	0.8649	0.4223	0.07407407407407407
At	897	Cupin_clan	1	16	896	10795	0.7423	0.6217	0.11148272017837235
Bd	998	Cupin_clan	2	16	996	10795	0.457	0.8093	0.2004008016032064
Cp	198	Cupin_clan	1	16	197	10795	0.2656	0.9633	0.5050505050505051
Cr	88	Cupin_clan	0	16	88	10795	1	0.8783	0
Cs	207	Cupin_clan	1	16	206	10795	0.2758	0.9603	0.4830917874396135
Gm*	702	Cupin_clan	4	16	698	10795	0.03038	0.994	0.5698005698005698
Me	323	Cupin_clan	1	16	322	10795	0.394	0.9143	0.30959752321981426
Mg	903	Cupin_clan	1	16	902	10795	0.7445	0.6187	0.11074197120708748
Mt	1148	Cupin_clan	1	16	1147	10795	0.8204	0.5044	0.08710801393728224
Os	971	Cupin_clan	0	16	971	10795	1	0.2523	0
Pp	258	Cupin_clan	1	16	257	10795	0.3305	0.9415	0.3875968992248062
Pt	425	Cupin_clan	1	16	424	10795	0.4811	0.8663	0.2352941176470588
Rc	250	Cupin_clan	0	16	250	10795	1	0.6935	0
Sb	817	Cupin_clan	0	16	817	10795	1	0.3115	0
Sm	544	Cupin_clan	0	16	544	10795	1	0.4556	0
Vv	315	Cupin_clan	1	16	314	10795	0.3865	0.9178	0.31746031746031744
Zm	417	Cupin_clan	0	16	417	10795	1	0.5456	0
Al	1350	DUF1618	4	16	1346	10795	0.1749	0.9371	0.2962962962962963
At	897	DUF1618	1	16	896	10795	0.7423	0.6217	0.11148272017837235
Bd	998	DUF1618	1	16	997	10795	0.7773	0.5726	0.1002004008016032
Cp	198	DUF1618	0	16	198	10795	1	0.7478	0
Cr	88	DUF1618	0	16	88	10795	1	0.8783	0
Cs	207	DUF1618	0	16	207	10795	1	0.7381	0
Gm	702	DUF1618	1	16	701	10795	0.6571	0.722	0.14245014245014245
Me	323	DUF1618	0	16	323	10795	1	0.6242	0
Mg	903	DUF1618	3	16	900	10795	0.1766	0.9464	0.33222591362126247
Mt	1148	DUF1618	0	16	1148	10795	1	0.1987	0
Os	971	DUF1618	1	16	970	10795	0.7685	0.5855	0.10298661174047373
Pp	258	DUF1618	1	16	257	10795	0.3305	0.9415	0.3875968992248062
Pt	425	DUF1618	1	16	424	10795	0.4811	0.8663	0.2352941176470588
Rc	250	DUF1618	2	16	248	10795	0.06139	0.9928	0.8
Sb	817	DUF1618	0	16	817	10795	1	0.3115	0
Sm	544	DUF1618	0	16	544	10795	1	0.4556	0
Vv	315	DUF1618	0	16	315	10795	1	0.6314	0
Zm	417	DUF1618	1	16	416	10795	0.4747	0.8702	0.2398081534772182
Al	1350	Actin_ATPase_CLA	1	15	1349	10796	0.848	0.4561	0.07407407407407407
At	897	Actin_ATPase_CLA	0	15	897	10796	1	0.3023	0
Bd	998	Actin_ATPase_CLA	1	15	997	10796	0.7568	0.603	0.1002004008016032
Cp	198	Actin_ATPase_CLA	1	15	197	10796	0.2522	0.9673	0.5050505050505051
Cr	88	Actin_ATPase_CLA	0	15	88	10796	1	0.8854	0
Cs	207	Actin_ATPase_CLA	1	15	206	10796	0.2619	0.9645	0.4830917874396135
Gm	702	Actin_ATPase_CLA	1	15	701	10796	0.6348	0.7452	0.14245014245014245
Me	323	Actin_ATPase_CLA	1	15	322	10796	0.3758	0.923	0.30959752321981426
Mg	903	Actin_ATPase_CLA	1	15	902	10796	0.7232	0.6473	0.11074197120708748
Mt	1148	Actin_ATPase_CLA	2	15	1146	10796	0.4956	0.7804	0.17421602787456447
Os	971	Actin_ATPase_CLA	2	15	969	10796	0.4145	0.84	0.20597322348094746
Pp	258	Actin_ATPase_CLA	0	15	258	10796	1	0.7019	0
Pt	425	Actin_ATPase_CLA	2	15	423	10796	0.1337	0.9754	0.4705882352941176
Rc	250	Actin_ATPase_CLA	0	15	250	10796	1	0.7095	0
Sb	817	Actin_ATPase_CLA	1	15	816	10796	0.6885	0.6886	0.12239902080783352
Sm	544	Actin_ATPase_CLA	0	15	544	10796	1	0.4786	0
Vv	315	Actin_ATPase_CLA	0	15	315	10796	1	0.6498	0
Zm	417	Actin_ATPase_CLA	1	15	416	10796	0.4544	0.8827	0.2398081534772182
Al	1350	SMI1_KNR4	0	15	1350	10796	1	0.171	0
At	897	SMI1_KNR4	0	15	897	10796	1	0.3023	0
Bd	998	SMI1_KNR4	0	15	998	10796	1	0.2657	0
Cp	198	SMI1_KNR4	0	15	198	10796	1	0.7615	0
Cr	88	SMI1_KNR4	0	15	88	10796	1	0.8854	0
Cs	207	SMI1_KNR4	0	15	207	10796	1	0.7523	0
Gm*	702	SMI1_KNR4	13	15	689	10796	0.000000002304	1	1.8518518518518516
Me	323	SMI1_KNR4	0	15	323	10796	1	0.6428	0
Mg	903	SMI1_KNR4	0	15	903	10796	1	0.3	0
Mt	1148	SMI1_KNR4	0	15	1148	10796	1	0.2199	0
Os	971	SMI1_KNR4	0	15	971	10796	1	0.275	0
Pp	258	SMI1_KNR4	0	15	258	10796	1	0.7019	0
Pt	425	SMI1_KNR4	0	15	425	10796	1	0.5606	0
Rc	250	SMI1_KNR4	0	15	250	10796	1	0.7095	0
Sb	817	SMI1_KNR4	0	15	817	10796	1	0.3351	0
Sm	544	SMI1_KNR4	0	15	544	10796	1	0.4786	0
Vv	315	SMI1_KNR4	2	15	313	10796	0.0822	0.9886	0.6349206349206349
Zm	417	SMI1_KNR4	0	15	417	10796	1	0.5666	0
Al	1350	kelch_CLAN+Glyoxal_oxid_N	3	14	1347	10797	0.2906	0.8886	0.2222222222222222
At	897	kelch_CLAN+Glyoxal_oxid_N	3	14	894	10797	0.1366	0.9636	0.33444816053511706
Bd	998	kelch_CLAN+Glyoxal_oxid_N	1	14	997	10797	0.7343	0.6342	0.1002004008016032
Cp	198	kelch_CLAN+Glyoxal_oxid_N	0	14	198	10797	1	0.7755	0
Cr	88	kelch_CLAN+Glyoxal_oxid_N	0	14	88	10797	1	0.8926	0
Cs	207	kelch_CLAN+Glyoxal_oxid_N	0	14	207	10797	1	0.7667	0
Gm	702	kelch_CLAN+Glyoxal_oxid_N	0	14	702	10797	1	0.4142	0
Me	323	kelch_CLAN+Glyoxal_oxid_N	0	14	323	10797	1	0.662	0
Mg	903	kelch_CLAN+Glyoxal_oxid_N	0	14	903	10797	1	0.3251	0
Mt	1148	kelch_CLAN+Glyoxal_oxid_N	1	14	1147	10797	0.7801	0.5705	0.08710801393728224
Os	971	kelch_CLAN+Glyoxal_oxid_N	0	14	971	10797	1	0.2997	0
Pp	258	kelch_CLAN+Glyoxal_oxid_N	1	14	257	10797	0.2981	0.9535	0.3875968992248062
Pt	425	kelch_CLAN+Glyoxal_oxid_N	1	14	424	10797	0.4394	0.8916	0.2352941176470588
Rc	250	kelch_CLAN+Glyoxal_oxid_N	0	14	250	10797	1	0.726	0
Sb	817	kelch_CLAN+Glyoxal_oxid_N	2	14	815	10797	0.3114	0.9024	0.24479804161566704
Sm	544	kelch_CLAN+Glyoxal_oxid_N	0	14	544	10797	1	0.5027	0
Vv	315	kelch_CLAN+Glyoxal_oxid_N	0	14	315	10797	1	0.6688	0
Zm	417	kelch_CLAN+Glyoxal_oxid_N	2	14	415	10797	0.1173	0.9801	0.4796163069544364
Al	1350	SMI1_KNR4+DUF525	1	14	1349	10797	0.829	0.4917	0.07407407407407407
At	897	SMI1_KNR4+DUF525	1	14	896	10797	0.6977	0.679	0.11148272017837235
Bd	998	SMI1_KNR4+DUF525	1	14	997	10797	0.7343	0.6342	0.1002004008016032
Cp	198	SMI1_KNR4+DUF525	1	14	197	10797	0.2385	0.971	0.5050505050505051
Cr	88	SMI1_KNR4+DUF525	1	14	87	10797	0.1146	0.9937	1.1363636363636365
Cs	207	SMI1_KNR4+DUF525	1	14	206	10797	0.2477	0.9686	0.4830917874396135
Gm	702	SMI1_KNR4+DUF525	1	14	701	10797	0.611	0.7683	0.14245014245014245
Me	323	SMI1_KNR4+DUF525	1	14	322	10797	0.3572	0.9313	0.30959752321981426
Mg	903	SMI1_KNR4+DUF525	1	14	902	10797	0.7	0.6763	0.11074197120708748
Mt	1148	SMI1_KNR4+DUF525	0	14	1148	10797	1	0.2432	0
Os	971	SMI1_KNR4+DUF525	0	14	971	10797	1	0.2997	0
Pp	258	SMI1_KNR4+DUF525	0	14	258	10797	1	0.7187	0
Pt	425	SMI1_KNR4+DUF525	2	14	423	10797	0.1209	0.9792	0.4705882352941176
Rc	250	SMI1_KNR4+DUF525	0	14	250	10797	1	0.726	0
Sb	817	SMI1_KNR4+DUF525	1	14	816	10797	0.665	0.7154	0.12239902080783352
Sm	544	SMI1_KNR4+DUF525	2	14	542	10797	0.177	0.9616	0.3676470588235294
Vv	315	SMI1_KNR4+DUF525	0	14	315	10797	1	0.6688	0
Zm	417	SMI1_KNR4+DUF525	0	14	417	10797	1	0.5885	0
Al	1350	FIST_C	1	14	1349	10797	0.829	0.4917	0.07407407407407407
At	897	FIST_C	0	14	897	10797	1	0.3274	0
Bd	998	FIST_C	0	14	998	10797	1	0.2903	0
Cp	198	FIST_C	1	14	197	10797	0.2385	0.971	0.5050505050505051
Cr	88	FIST_C	0	14	88	10797	1	0.8926	0
Cs	207	FIST_C	0	14	207	10797	1	0.7667	0
Gm	702	FIST_C	2	14	700	10797	0.2548	0.9302	0.2849002849002849
Me*	323	FIST_C	4	14	319	10797	0.001541	0.9999	1.238390092879257
Mg	903	FIST_C	2	14	901	10797	0.3527	0.8794	0.22148394241417496
Mt	1148	FIST_C	0	14	1148	10797	1	0.2432	0
Os	971	FIST_C	0	14	971	10797	1	0.2997	0
Pp	258	FIST_C	0	14	258	10797	1	0.7187	0
Pt	425	FIST_C	2	14	423	10797	0.1209	0.9792	0.4705882352941176
Rc	250	FIST_C	1	14	249	10797	0.2905	0.956	0.4
Sb	817	FIST_C	0	14	817	10797	1	0.3604	0
Sm	544	FIST_C	0	14	544	10797	1	0.5027	0
Vv	315	FIST_C	1	14	314	10797	0.3502	0.9342	0.31746031746031744
Zm	417	FIST_C	0	14	417	10797	1	0.5885	0
Al	1350	FBA_CLAN+Rod_C	0	13	1350	10798	1	0.2164	0
At	897	FBA_CLAN+Rod_C	0	13	897	10798	1	0.3546	0
Bd	998	FBA_CLAN+Rod_C	0	13	998	10798	1	0.3171	0
Cp	198	FBA_CLAN+Rod_C	0	13	198	10798	1	0.7897	0
Cr	88	FBA_CLAN+Rod_C	0	13	88	10798	1	0.9	0
Cs	207	FBA_CLAN+Rod_C	0	13	207	10798	1	0.7814	0
Gm	702	FBA_CLAN+Rod_C	0	13	702	10798	1	0.4412	0
Me	323	FBA_CLAN+Rod_C	0	13	323	10798	1	0.6819	0
Mg*	903	FBA_CLAN+Rod_C	13	13	890	10798	0.0000000126	1	1.4396456256921373
Mt	1148	FBA_CLAN+Rod_C	0	13	1148	10798	1	0.2691	0
Os	971	FBA_CLAN+Rod_C	0	13	971	10798	1	0.3267	0
Pp	258	FBA_CLAN+Rod_C	0	13	258	10798	1	0.7358	0
Pt	425	FBA_CLAN+Rod_C	0	13	425	10798	1	0.6056	0
Rc	250	FBA_CLAN+Rod_C	0	13	250	10798	1	0.7428	0
Sb	817	FBA_CLAN+Rod_C	0	13	817	10798	1	0.3877	0
Sm	544	FBA_CLAN+Rod_C	0	13	544	10798	1	0.528	0
Vv	315	FBA_CLAN+Rod_C	0	13	315	10798	1	0.6883	0
Zm	417	FBA_CLAN+Rod_C	0	13	417	10798	1	0.6112	0
Al	1350	Flavi_capsid	0	13	1350	10798	1	0.2164	0
At	897	Flavi_capsid	1	13	896	10798	0.6726	0.7082	0.11148272017837235
Bd	998	Flavi_capsid	2	13	996	10798	0.3658	0.8723	0.2004008016032064
Cp	198	Flavi_capsid	0	13	198	10798	1	0.7897	0
Cr	88	Flavi_capsid	0	13	88	10798	1	0.9	0
Cs	207	Flavi_capsid	0	13	207	10798	1	0.7814	0
Gm	702	Flavi_capsid	1	13	701	10798	0.5858	0.7913	0.14245014245014245
Me	323	Flavi_capsid	0	13	323	10798	1	0.6819	0
Mg	903	Flavi_capsid	1	13	902	10798	0.675	0.7056	0.11074197120708748
Mt*	1148	Flavi_capsid	6	13	1142	10798	0.007001	0.9987	0.5226480836236934
Os	971	Flavi_capsid	1	13	970	10798	0.7003	0.6771	0.10298661174047373
Pp	258	Flavi_capsid	0	13	258	10798	1	0.7358	0
Pt	425	Flavi_capsid	0	13	425	10798	1	0.6056	0
Rc	250	Flavi_capsid	0	13	250	10798	1	0.7428	0
Sb	817	Flavi_capsid	1	13	816	10798	0.6396	0.7422	0.12239902080783352
Sm	544	Flavi_capsid	0	13	544	10798	1	0.528	0
Vv	315	Flavi_capsid	0	13	315	10798	1	0.6883	0
Zm	417	Flavi_capsid	0	13	417	10798	1	0.6112	0
Al	1350	Beta_propeller_CLAN+DUF295	0	12	1350	10799	1	0.2435	0
At	897	Beta_propeller_CLAN+DUF295	1	12	896	10799	0.6454	0.7375	0.11148272017837235
Bd	998	Beta_propeller_CLAN+DUF295	1	12	997	10799	0.6829	0.6981	0.1002004008016032
Cp	198	Beta_propeller_CLAN+DUF295	0	12	198	10799	1	0.8042	0
Cr	88	Beta_propeller_CLAN+DUF295	0	12	88	10799	1	0.9073	0
Cs	207	Beta_propeller_CLAN+DUF295	0	12	207	10799	1	0.7964	0
Gm	702	Beta_propeller_CLAN+DUF295	0	12	702	10799	1	0.4699	0
Me	323	Beta_propeller_CLAN+DUF295	1	12	322	10799	0.3181	0.947	0.30959752321981426
Mg	903	Beta_propeller_CLAN+DUF295	0	12	903	10799	1	0.3817	0
Mt	1148	Beta_propeller_CLAN+DUF295	0	12	1148	10799	1	0.2977	0
Os	971	Beta_propeller_CLAN+DUF295	3	12	968	10799	0.1207	0.97	0.30895983522142123
Pp	258	Beta_propeller_CLAN+DUF295	0	12	258	10799	1	0.7534	0
Pt	425	Beta_propeller_CLAN+DUF295	1	12	424	10799	0.3944	0.9154	0.2352941176470588
Rc	250	Beta_propeller_CLAN+DUF295	0	12	250	10799	1	0.76	0
Sb	817	Beta_propeller_CLAN+DUF295	1	12	816	10799	0.6123	0.769	0.12239902080783352
Sm	544	Beta_propeller_CLAN+DUF295	0	12	544	10799	1	0.5546	0
Vv	315	Beta_propeller_CLAN+DUF295	0	12	315	10799	1	0.7083	0
Zm*	417	Beta_propeller_CLAN+DUF295	4	12	413	10799	0.002392	0.9998	0.9592326139088728
Al	1350	FBA_CLAN+PRANC	2	11	1348	10800	0.4319	0.8317	0.14814814814814814
At*	897	FBA_CLAN+PRANC	4	11	893	10800	0.02361	0.996	0.4459308807134894
Bd	998	FBA_CLAN+PRANC	0	11	998	10800	1	0.3784	0
Cp	198	FBA_CLAN+PRANC	0	11	198	10800	1	0.819	0
Cr	88	FBA_CLAN+PRANC	0	11	88	10800	1	0.9146	0
Cs	207	FBA_CLAN+PRANC	0	11	207	10800	1	0.8116	0
Gm	702	FBA_CLAN+PRANC	0	11	702	10800	1	0.5004	0
Me	323	FBA_CLAN+PRANC	0	11	323	10800	1	0.7233	0
Mg	903	FBA_CLAN+PRANC	3	11	900	10800	0.08774	0.9812	0.33222591362126247
Mt	1148	FBA_CLAN+PRANC	2	11	1146	10800	0.359	0.878	0.17421602787456447
Os	971	FBA_CLAN+PRANC	0	11	971	10800	1	0.3881	0
Pp	258	FBA_CLAN+PRANC	0	11	258	10800	1	0.7714	0
Pt	425	FBA_CLAN+PRANC	0	11	425	10800	1	0.6542	0
Rc	250	FBA_CLAN+PRANC	0	11	250	10800	1	0.7776	0
Sb	817	FBA_CLAN+PRANC	0	11	817	10800	1	0.4486	0
Sm	544	FBA_CLAN+PRANC	0	11	544	10800	1	0.5826	0
Vv	315	FBA_CLAN+PRANC	0	11	315	10800	1	0.729	0
Zm	417	FBA_CLAN+PRANC	0	11	417	10800	1	0.6593	0
Al	1350	AAA_CLAN+zf-CW	0	10	1350	10801	1	0.3082	0
At	897	AAA_CLAN+zf-CW	0	10	897	10801	1	0.4505	0
Bd	998	AAA_CLAN+zf-CW	1	10	997	10801	0.6216	0.763	0.1002004008016032
Cp	198	AAA_CLAN+zf-CW	0	10	198	10801	1	0.834	0
Cr	88	AAA_CLAN+zf-CW	0	10	88	10801	1	0.9221	0
Cs	207	AAA_CLAN+zf-CW	1	10	206	10801	0.1884	0.9827	0.4830917874396135
Gm	702	AAA_CLAN+zf-CW	1	10	701	10801	0.4996	0.8582	0.14245014245014245
Me	323	AAA_CLAN+zf-CW	1	10	322	10801	0.2767	0.9612	0.30959752321981426
Mg	903	AAA_CLAN+zf-CW	0	10	903	10801	1	0.4482	0
Mt	1148	AAA_CLAN+zf-CW	0	10	1148	10801	1	0.3644	0
Os	971	AAA_CLAN+zf-CW	1	10	970	10801	0.6119	0.7719	0.10298661174047373
Pp	258	AAA_CLAN+zf-CW	1	10	257	10801	0.2286	0.974	0.3875968992248062
Pt	425	AAA_CLAN+zf-CW	1	10	424	10801	0.3458	0.9374	0.2352941176470588
Rc	250	AAA_CLAN+zf-CW	1	10	249	10801	0.2224	0.9755	0.4
Sb	817	AAA_CLAN+zf-CW	1	10	816	10801	0.5514	0.8218	0.12239902080783352
Sm	544	AAA_CLAN+zf-CW	0	10	544	10801	1	0.6119	0
Vv	315	AAA_CLAN+zf-CW	0	10	315	10801	1	0.7503	0
Zm	417	AAA_CLAN+zf-CW	1	10	416	10801	0.3407	0.9394	0.2398081534772182
Al	1350	Ste50p-SAM	0	10	1350	10801	1	0.3082	0
At	897	Ste50p-SAM	0	10	897	10801	1	0.4505	0
Bd	998	Ste50p-SAM	0	10	998	10801	1	0.4134	0
Cp	198	Ste50p-SAM	1	10	197	10801	0.181	0.984	0.5050505050505051
Cr	88	Ste50p-SAM	0	10	88	10801	1	0.9221	0
Cs	207	Ste50p-SAM	1	10	206	10801	0.1884	0.9827	0.4830917874396135
Gm	702	Ste50p-SAM	2	10	700	10801	0.1637	0.9672	0.2849002849002849
Me	323	Ste50p-SAM	0	10	323	10801	1	0.7449	0
Mg	903	Ste50p-SAM	1	10	902	10801	0.5864	0.794	0.11074197120708748
Mt	1148	Ste50p-SAM	1	10	1147	10801	0.6706	0.7144	0.08710801393728224
Os	971	Ste50p-SAM	2	10	969	10801	0.2598	0.93	0.20597322348094746
Pp	258	Ste50p-SAM	1	10	257	10801	0.2286	0.974	0.3875968992248062
Pt	425	Ste50p-SAM	0	10	425	10801	1	0.68	0
Rc	250	Ste50p-SAM	1	10	249	10801	0.2224	0.9755	0.4
Sb	817	Ste50p-SAM	0	10	817	10801	1	0.4825	0
Sm	544	Ste50p-SAM	0	10	544	10801	1	0.6119	0
Vv	315	Ste50p-SAM	0	10	315	10801	1	0.7503	0
Zm	417	Ste50p-SAM	0	10	417	10801	1	0.6848	0
Single asterisk indicates significant enrichment (p<0.05).  Double asterisks indicate significant depletion (p<0.05).
